# Supplementary material for: Top-down semantic predictions align phonetic neuronal dynamics in human superior temporal gyrus
Source: Nat Commun. 2026 Oct 3;17:11313. doi: 10.1038/s41467-026-77942-x (PMC13724739; doi:10.1038/s41467-026-77942-x)
Supplement: Supplementary file 1 [file 41467_2026_77942_MOESM1_ESM.pdf]

Supplementary Material: Top-down semantic predictions align phonetic neuronal dynamics in human superior temporal gyrus

Pavo Orepic[1], Wilson Truccolo[2,3], Eric Halgren[4], Sydney S. Cash[5], Anne-Lise Giraud[1,6], Timothée Proix[7*]

[1]Department of Basic Neurosciences, Faculty of Medicine, University of Geneva, Geneva, Switzerland.
[2]Department of Neuroscience, Brown University, Providence, Rhode Island, United States of America.
[3]Carney Institute for Brain Science, Brown University, Providence, Rhode Island, United States of America.
[4]Department of Neuroscience & Radiology, University of California San Diego, La Jolla, California, United States of America.
[5]Department of Neurology, Massachusetts General Hospital, Harvard Medical School, Boston, Massachusetts, United States of America.
[6]Institut Pasteur, Université Paris Cité, Hearing Institute, Paris, France.
[7]Institute of Neuroinformatics, University of Zurich and ETH Zurich, Zurich, Switzerland.

*Corresponding author(s). E-mail(s): timothee.proix.neuro@pm.me;

Phonetic and semantic mTRF features

Supplementary Table 1 | Vowels used in the mTRF models for the semantic categorization task. Duration is computed as the mean in milliseconds across all occurrences of the same phoneme.

	First formant		*Second formant*			*Task: participants 1 and 2*		*Speech: participant 1*		*Speech: participant 3*	
**Vowel**	**Low**	**High**	**Front**	**Middle**	**Back**	**Repetitions**	**Duration**	**Repetitions**	**Duration**	**Repetitions**	**Duration**
/ae/	1	0	1	0	0	64	87.8	34	142.9	90	117.4
/ʌ/	1	0	0	0	1	103	47.0	165	60.7	246	55.0
/ɪ/	0	1	1	0	0	61	56.1	116	77.6	205	84.1
/ɜ/	1	0	0	1	0	43	89.4	43	93.0	61	66.0
/uː/	0	1	0	0	1	31	81.8	30	114.0	50	129.5
/ɒ/	1	0	0	0	1	49	73.4	53	104.2	68	72.4
/ɛ/	1	0	1	0	0	44	66.4	63	94.6	121	118.0
/ɔ/	1	0	0	0	1	29	91.5	29	138.9	44	112.7
/i/	0	1	1	0	0	55	89.6	77	112.8	150	108.4
/u/	0	1	0	0	1	7	57.1	13	78.1	14	64.3
/ʊ/	0	1	0	0	1	8	33.8	7	102.9	23	78.3

Supplementary Table 2 | Consonants used in the mTRF models. Duration is computed as the mean in milliseconds across all occurrences of the same phoneme.

	Articulation place						*Articulation manner*					*Task: participants 1 and 2*		*Speech: participant 1*		*Speech: participant 3*	
**Consonant**	**Bilabial**	**Labiodental**	**Alveolar**	**Velar**	**Uvular**	**Glottal**	**Plosive**	**Nasal**	**Fricative**	**Approximant**	**Lateral approximant**	**Repetitions**	**Duration**	**Repetitions**	**Duration**	**Repetitions**	**Duration**
/ŋ/	0	0	0	1	0	0	0	1	0	0	0	19	52.8	26	65.4	51	82.1
/k/	0	0	0	1	0	0	1	0	0	0	0	135	41.0	43	41.4	105	67.0
/l/	0	0	1	0	0	0	0	0	0	0	1	123	47.9	76	73.9	117	58.4
/t/	0	0	1	0	0	0	1	0	0	0	0	120	53.0	83	46.0	223	101.9
/n/	0	0	1	0	0	0	0	1	0	0	0	89	46.2	123	118.0	223	125.1
/p/	1	0	0	0	0	0	1	0	0	0	0	75	36.7	22	43.2	61	67.7
/f/	0	1	0	0	0	0	0	0	1	0	0	45	36.7	26	33.2	42	120.8
/d/	0	0	1	0	0	0	1	0	0	0	0	51	34.0	39	40.5	109	66.9
/ʁ/	0	0	0	0	1	0	0	0	1	0	0	100	38.3	64	53.8	96	34.8
/ʃ/	0	0	1	0	0	0	0	0	1	0	0	22	70.7	25	106.5	24	110.4
/s/	0	0	1	0	0	0	0	0	1	0	0	104	65.0	108	112.8	151	102.0
/b/	1	0	0	0	0	0	1	0	0	0	0	79	22.0	13	39.2	64	40.2
/ʥ/	0	0	1	0	0	0	0	0	1	0	0	18	59.0	10	63.0	20	82.0
/m/	1	0	0	0	0	0	0	1	0	0	0	60	46.6	46	64.9	104	181.2
/g/	0	0	0	1	0	0	1	0	0	0	0	36	35.0	34	60.3	52	40.8
/v/	0	1	0	0	0	0	0	0	1	0	0	13	28.5	24	45.5	38	84.9
/h/	0	0	0	0	0	1	0	0	1	0	0	25	37.9	27	41.2	39	49.3
/z/	0	0	1	0	0	0	0	0	1	0	0	13	50.0	55	111.2	91	129.8
/ʨ/	0	0	1	0	0	0	0	0	1	0	0	31	67.8	70	41.4	136	54.5
/w/	0	0	0	1	0	0	0	0	0	1	0	22	35.8	70	58.5	97	41.5
/θ/	0	0	1	0	0	0	0	0	1	0	0	10	29.0	19	34.4	39	159.4

Supplementary Table 3 | Semantic features for the semantic categorization task (participants 1 and 2).

	Semantic features	Repetitions
Conceptual category	Object	200
	Animal	200
Perceptual category	Bigger than a foot	200
	Smaller than a foot	200
Semantic decision (participant 1)	Bigger than a foot	139
	Smaller than a foot	261
Semantic decision (participant 2)	Bigger than a foot	224
	Smaller than a foot	176

Supplementary Table 4 | Lexical semantics feature for natural speech perception - participants 1 and 3. Values represent average strength for each Lancaster norm for each participant across all words.

Lancaster sensorimotor norm	Participant 1 (664 words)	Participant 3 (1137 words)
Auditory	1.83	1.85
Gustatory	0.36	0.41
Haptic	1.13	1.17
Interoceptive	1.2	1.31
Olfactory	0.44	0.54
Visual	2.73	2.77
Foot, leg	1.03	1.04
Hand, arm	1.49	1.52
Head	2.37	2.50
Mouth	1.4	1.52
Torso	0.91	0.95

Raster plots, control analyses, mTRF models for phonetic groups, and PCA details

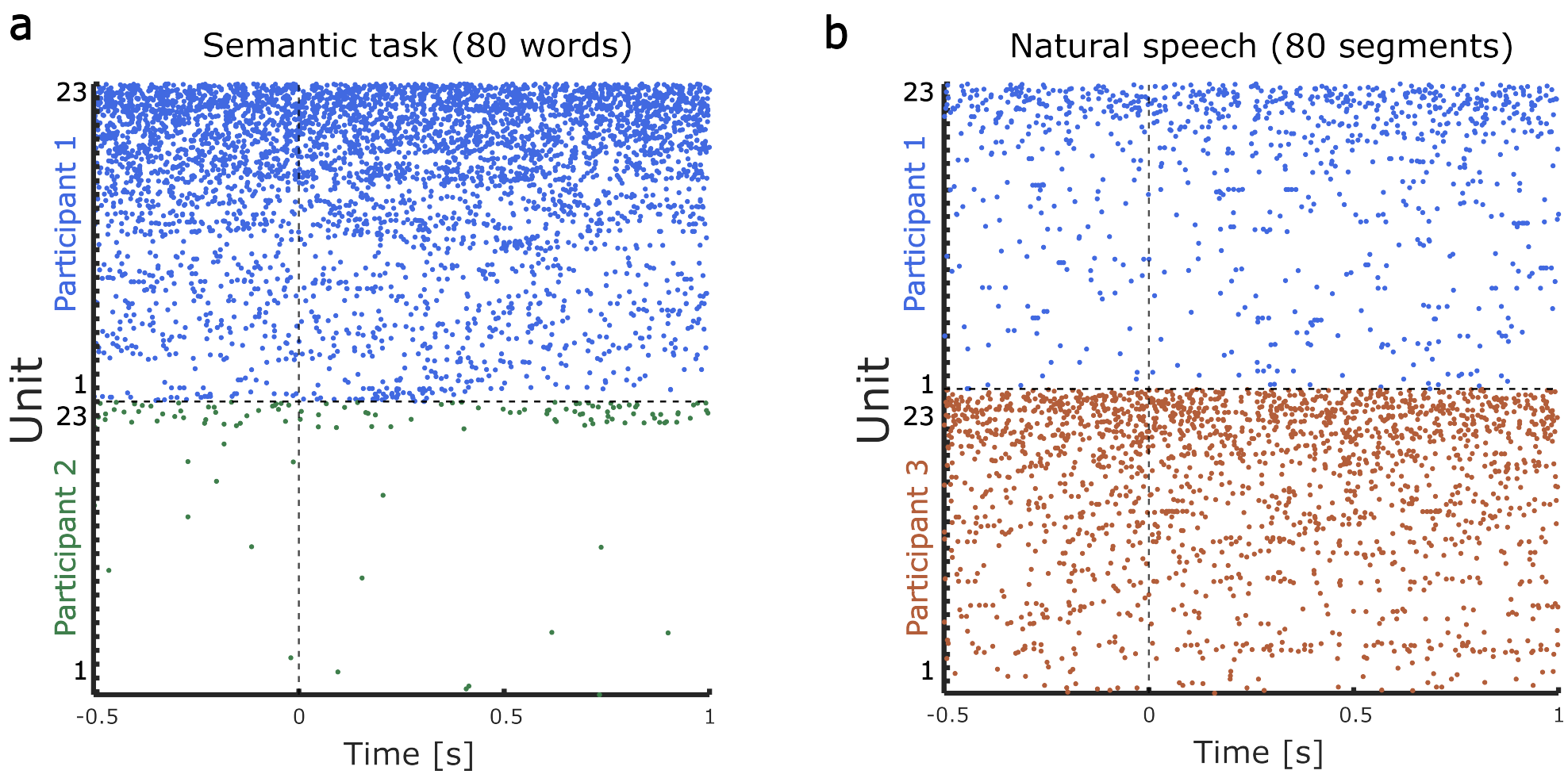

Supplementary Fig. 1 | Raster plots a. Raster plots for the semantic-task participants (1 and 2) for 23 most-spiking units and for 80 random words. Time 0 indicates word onset and all words were 0.5 seconds long. Colors indicate participants as in the main Fig. 1. Despite sparse spiking in participant 2, we still observed the same population effects as for participant 1 (main Fig. 2). **b.** Raster plots for the natural-speech participants (1 and 3) for 23-most spiking units and for 80 random speech segments of the same duration as in the semantic task (1.5 seconds). Time 0 indicates word onset during natural speech, but each word was of a different length, and there was speech before and after the word during the 1.5-second period. Colors indicate participants as in the main Fig. 3.

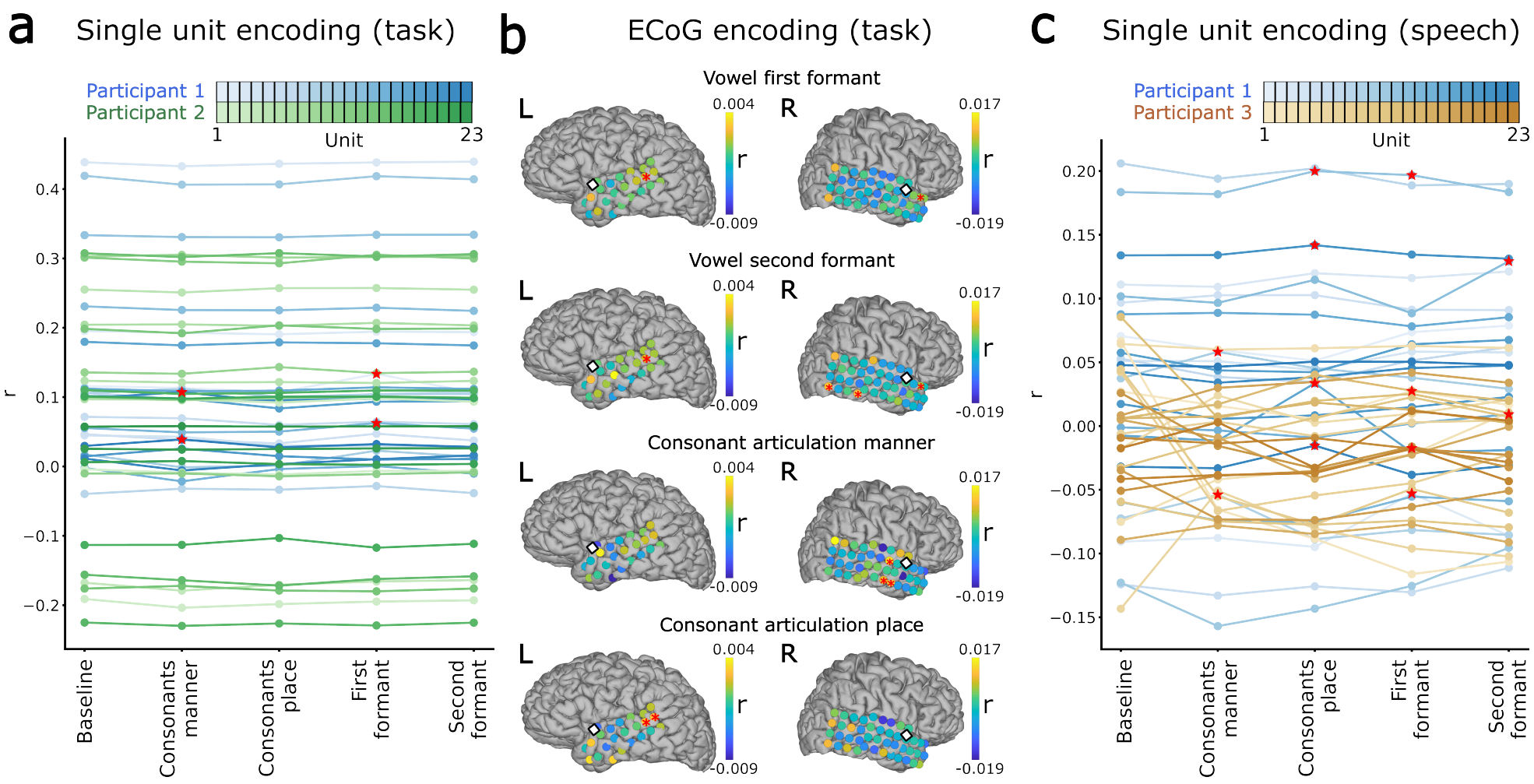

Supplementary Fig. 2 | Phonetic encoding at the single-unit and LFP level in the aSTG. **a.** Pearson correlation coefficient (r values) for models including each phonetic feature group fitted to each of the 23 most-spiking single units in the ensemble for the semantic-task participants 1 (blue) and 2 (green). For both participants, the lines for different units are color-coded based on firing rate, from lower (light) to higher (dark). Red stars indicate units for which r values of the fitted models are significantly higher ($p < 0.05$) than the chance level. **b** Encoding of vowel first formant, vowel second formant, consonant manner of articulation, and consonant place of articulation across ECoG channels in the two semantic-task participants. Colors indicate differences in r values compared to the baseline model. Red stars indicate significance as above. Squares indicate MEAs. **c.** Pearson correlation coefficient (r values) for models including each phonetic feature group fitted to each of the 23 most-spiking single units in the ensemble for the natural speech participants 1 (blue) and 3 (orange). For both participants, the lines for different units are color-coded based on firing rate, from lower (light) to higher (dark). Red stars indicate units for which r values of the fitted models are significantly higher ($p < 0.05$) than the chance level.

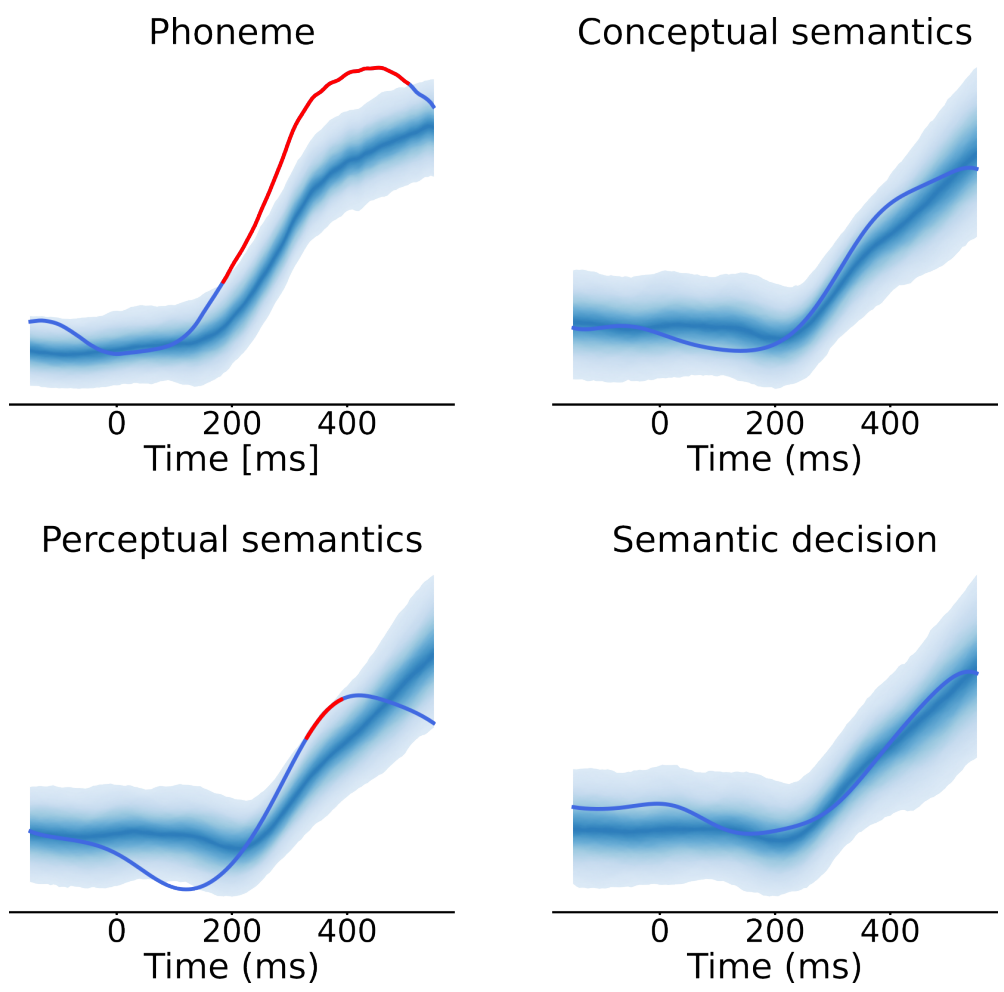

Supplementary Fig. 3 Phonetic and semantic kernels using all units in Participant 1. To test whether the mTRF results depended on selecting the 23 highest-spiking units, we repeated the main phonetic and semantic encoding analyses for Participant 1 using all recorded units. Specifically, we refit the same phonetic and semantic mTRF models used in Fig. 2a, but included the full set of units recorded from Participant 1 rather than restricting the analysis to the highest-spiking subset. The four panels show the phonetic and semantic kernels for the same model components shown in Fig. 2a. Lines indicate the mean kernel across units. The 95% confidence region of the surrogate (chance-level) distribution is shown with brighter shading for increasingly peripheral percentiles. The resulting kernels preserved the same overall temporal profiles as the kernels reported in the main text. This indicates that the main phonetic and semantic response patterns were not driven solely by the unit-selection criterion.

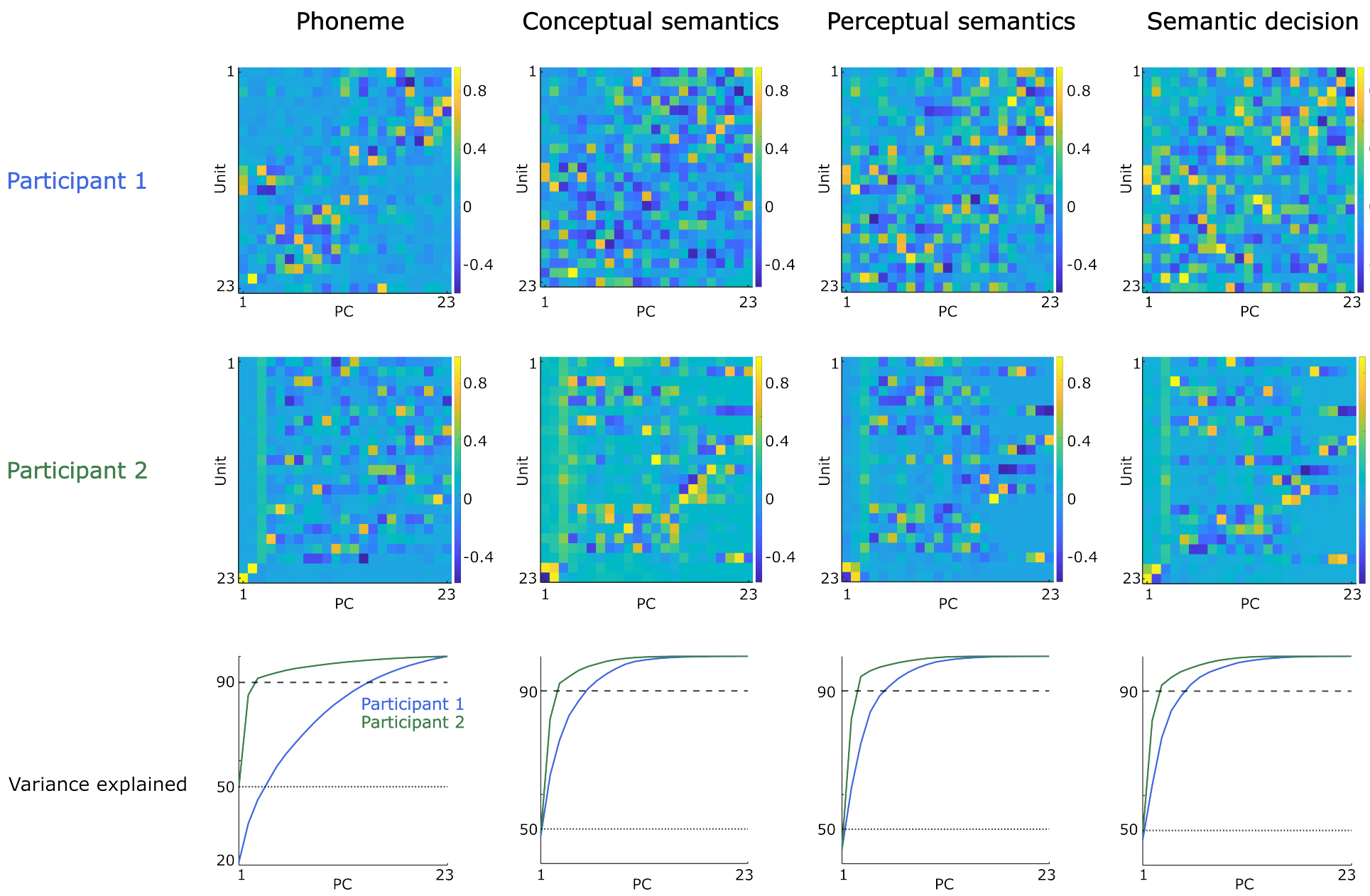

Supplementary Fig. 4 | Semantic categorization task PCA Principal component (PC) coefficients of isolated single units sorted by increasing firing rate for Participant 1 (first row) and Participant 2 (second row), and variance explained (third row) for phonetic and semantic kernels used in the semantic task. Each column indicates the PCs for different mTRF features from main Fig. 2a, respectively phoneme, conceptual semantics, perceptual semantics, and semantic decision. For Participant 1, several units are represented in the first PCs, indicating distributed encoding across the 23 units. For Participant 2, despite most variance being explained by the 2 most-spiking units, distributed encoding of all units showed population effects (main Fig 2c and 2d).

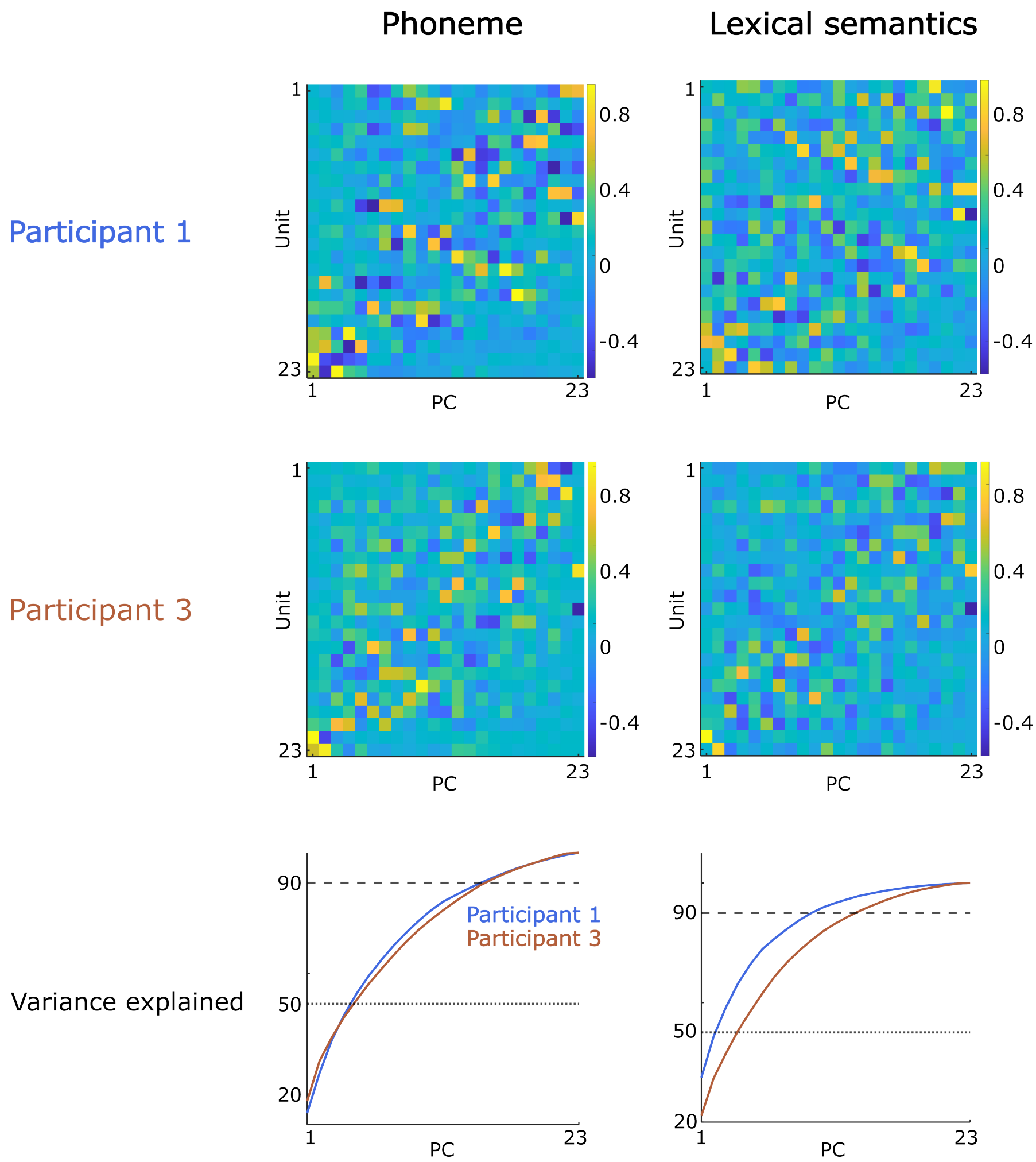

Supplementary Fig. 5 | **Natural speech PCA** Principal component (PC) coefficients of isolated single units sorted increasingly by firing rate for Participant 1 (first row) and Participant 3 (second row), and variance explained (third row) for phonetic and semantic kernels used in natural speech. Each column indicates the PCs for different mTRF features, respectively phoneme (main Fig. 3b) and lexical semantics (main Fig. 3d). Distributed encoding is apparent in both participants for both features, and reflected in population effects (main Fig 3c and 3d).

Clustering control analyses for the semantic task

To confirm our clustering results, we conducted several control analyses. We first confirmed that our results did not change when using PC spaces with more than two PC dimensions. Thus, we computed the clustering index for all four phonetic features (vowel first formant, vowel second formant, consonant manner of articulation, consonant place of articulation - Supplementary Figs. 6 and 7) and semantic features (perceptual, conceptual, decision - Supplementary Figs. 8 and 9) up to the sixth PC dimension. The results observed for two dimensions largely generalized when using more dimensions for the PC space.

For the vowel second formant group, we additionally ascertained that the lack of clustering was not caused by the fact that it contained three phonetic features (front, middle, and back position of the tongue) instead of two as for the first formant group (high, low). We replicated the lack of clustering effects by using two instead of three phonetic features (front and back position of the tongue, Supplementary Fig. 10).

To confirm the observed clustering of phonemes, we performed three alternative analyses: (i) linear discriminant analysis (LDA) classifier (Supplementary Fig. 11); (ii) for vowels, rank regression analysis (rank regression was performed to test whether vowels not only separate, but are also ordered by their first formant value in the PC space, Supplementary Fig. 12); (iii) k-means clustering (Supplementary Fig. 13). To reduce the number of plots, these alternative analyses are shown only for Participant 1, but the same was observed for Participant 2.

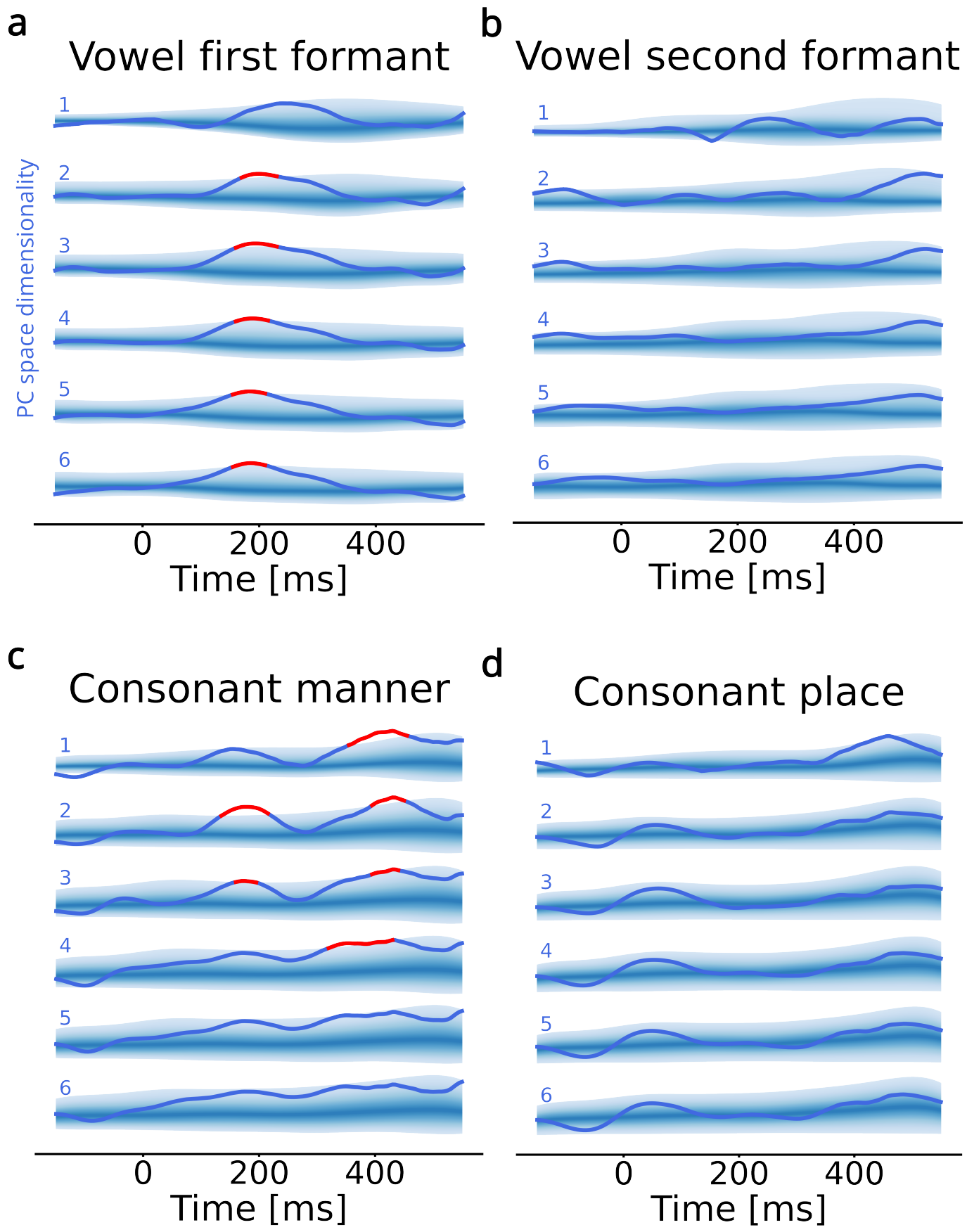

Supplementary Fig. 6 | Clustering index for phonetic features across dimensions during semantic categorization task in Participant 1. a. Clustering index for vowels grouped by first formant (high and low tongue position) up to six PC dimensions. The 95% confidence region of the surrogate (chance-level) distribution is shown with brighter shading for increasingly peripheral percentiles. Red segments indicate significant periods after multiple comparison correction (cluster-based test). Numbers indicate the dimensionality of the PC space. **b.** Clustering index for vowels grouped by second formant (front to back tongue position) up to six PC dimensions. Coloring and numbering as in a. **c.** Clustering index for consonants grouped by manner of articulation (plosive, nasal, fricative, approximant, and lateral approximant) up to six PC dimensions. Coloring and numbering as in a. **d.** Clustering index for consonants grouped by place of articulation (bilabial, labiodental, dental, alveolar, velar, uvular, and glottal) up to six PC dimensions. Coloring and numbering as in a.

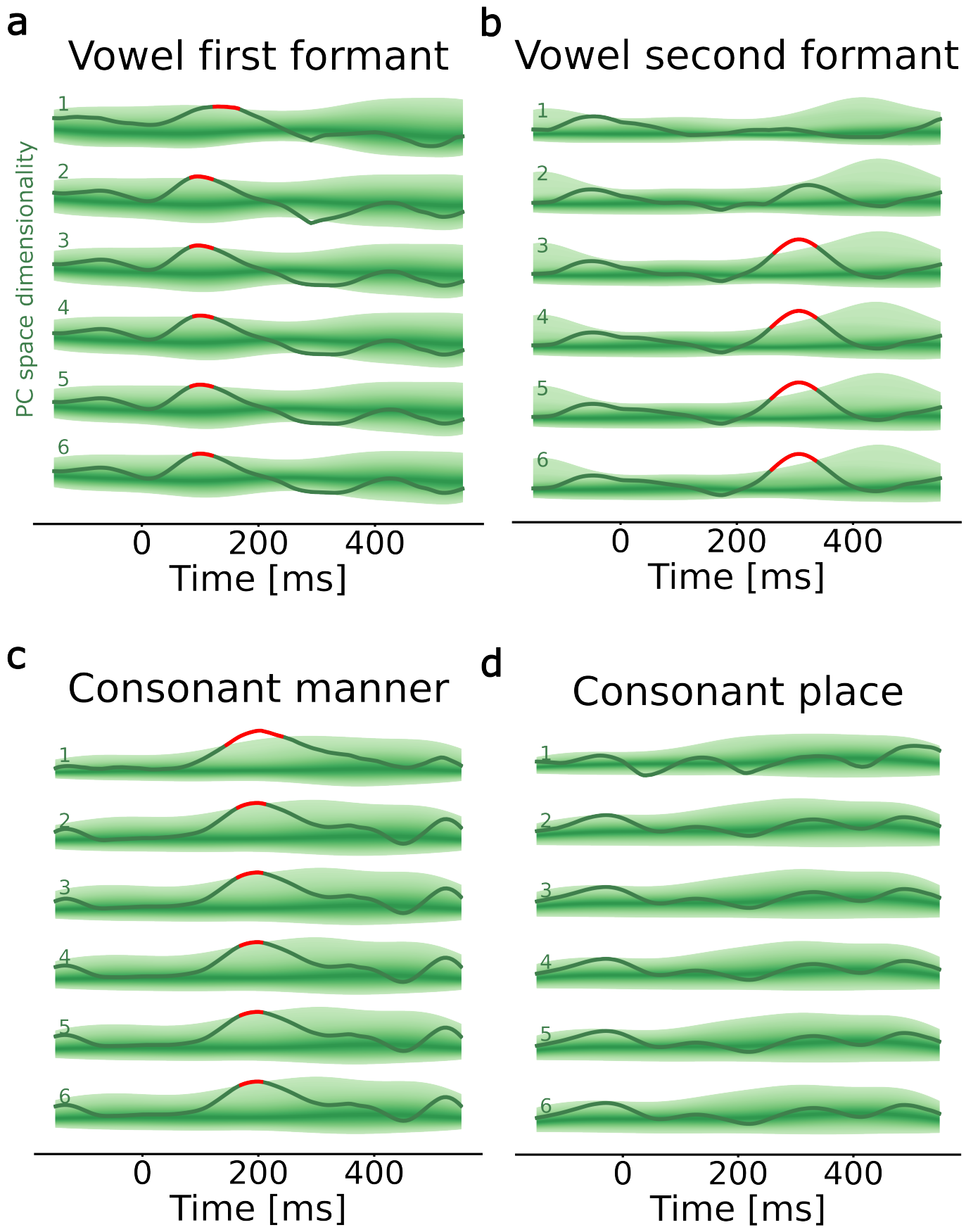

Supplementary Fig. 7 | Clustering index for phonetic features across dimensions during semantic categorization task in Participant 2. a. Clustering index for vowels grouped by first formant (high and low tongue position) up to six PC dimensions. The 95% confidence region of the surrogate (chance-level) distribution is shown with brighter shading for increasingly peripheral percentiles. Red segments indicate significant periods after multiple comparison correction (cluster-based test). Numbers indicate the dimensionality of the PC space. **b.** Clustering index for vowels grouped by second formant (front to back tongue position) up to six PC dimensions. Coloring and numbering as in a. **c.** Clustering index for consonants grouped by manner of articulation (plosive, nasal, fricative, approximant, and lateral approximant) up to six PC dimensions. Coloring and numbering as in a. **d.** Clustering index for consonants grouped by place of articulation (bilabial, labiodental, dental, alveolar, velar, uvular, and glottal) up to six PC dimensions. Coloring and numbering as in a.

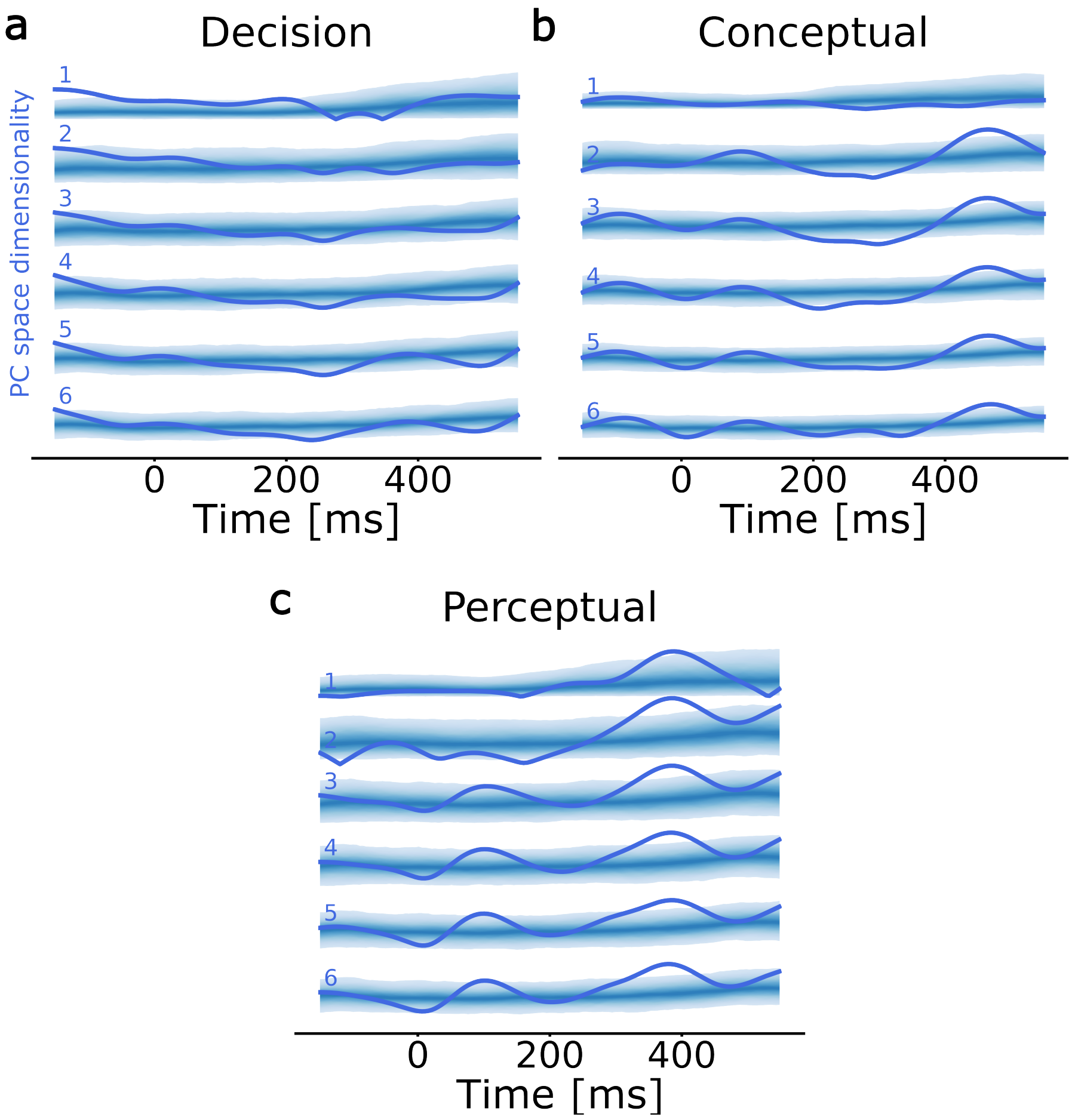

Supplementary Fig. 8 | Clustering index for semantic features across dimensions during semantic categorization task in Participant 1. a. Clustering index for semantic decision (response bigger vs. smaller) kernels. The 95% confidence region of the surrogate (chance-level) distribution is shown with brighter shading for increasingly peripheral percentiles. **b.** Clustering index for conceptual semantics (object vs. animal). Coloring and numbering as in a. **c.** Clustering index for perceptual semantics (bigger vs. smaller). Coloring and numbering as in a.

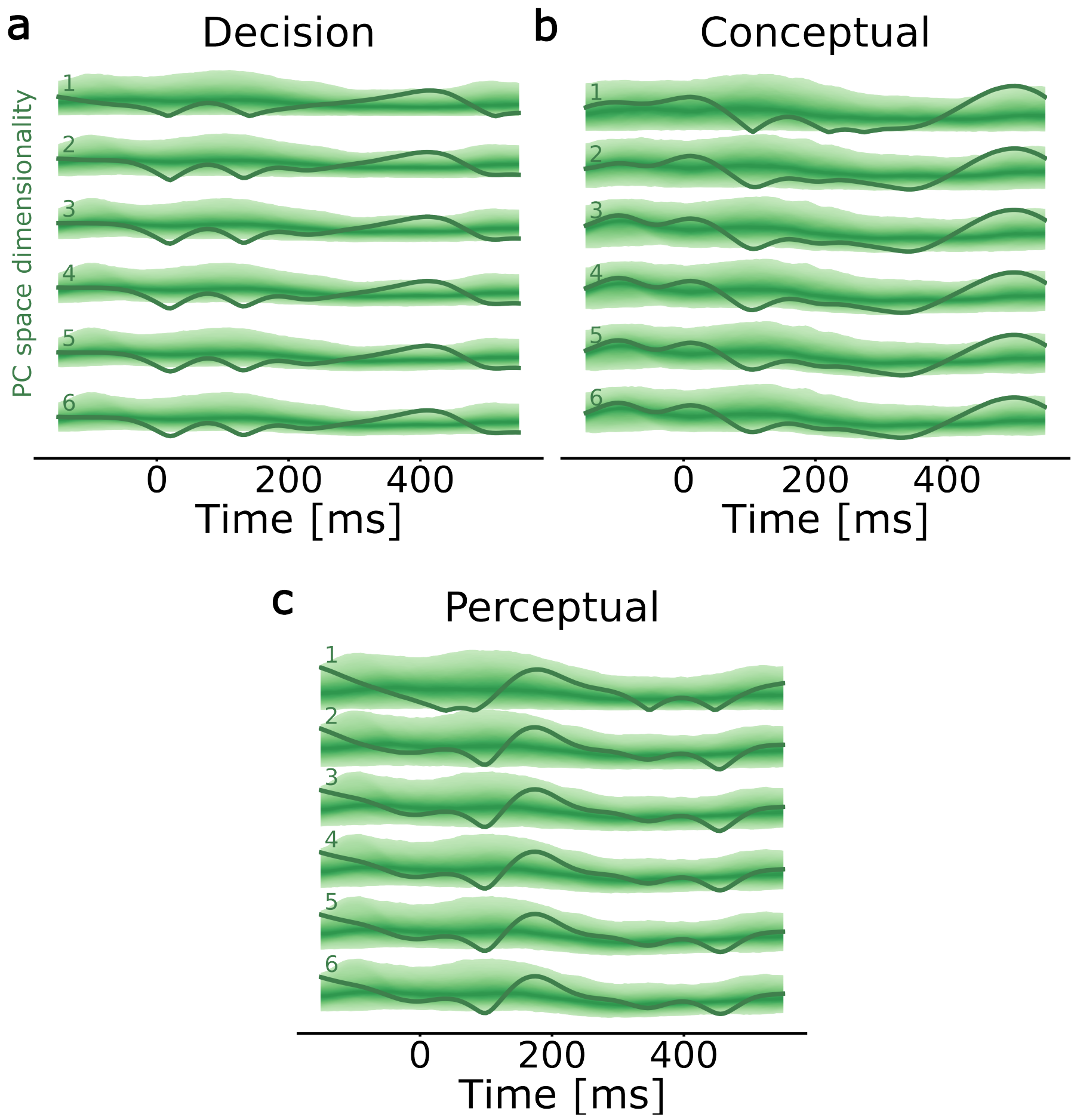

Supplementary Fig. 9 | Clustering index for semantic features across dimensions during semantic categorization task in Participant 2. a. Clustering index for semantic decision (response bigger vs. smaller) kernels. The 95% confidence region of the surrogate (chance-level) distribution is shown with brighter shading for increasingly peripheral percentiles. **b.** Clustering index for conceptual semantics (object vs. animal). Coloring and numbering as in a. **c.** Clustering index for perceptual semantics (bigger vs. smaller). Coloring and numbering as in a.

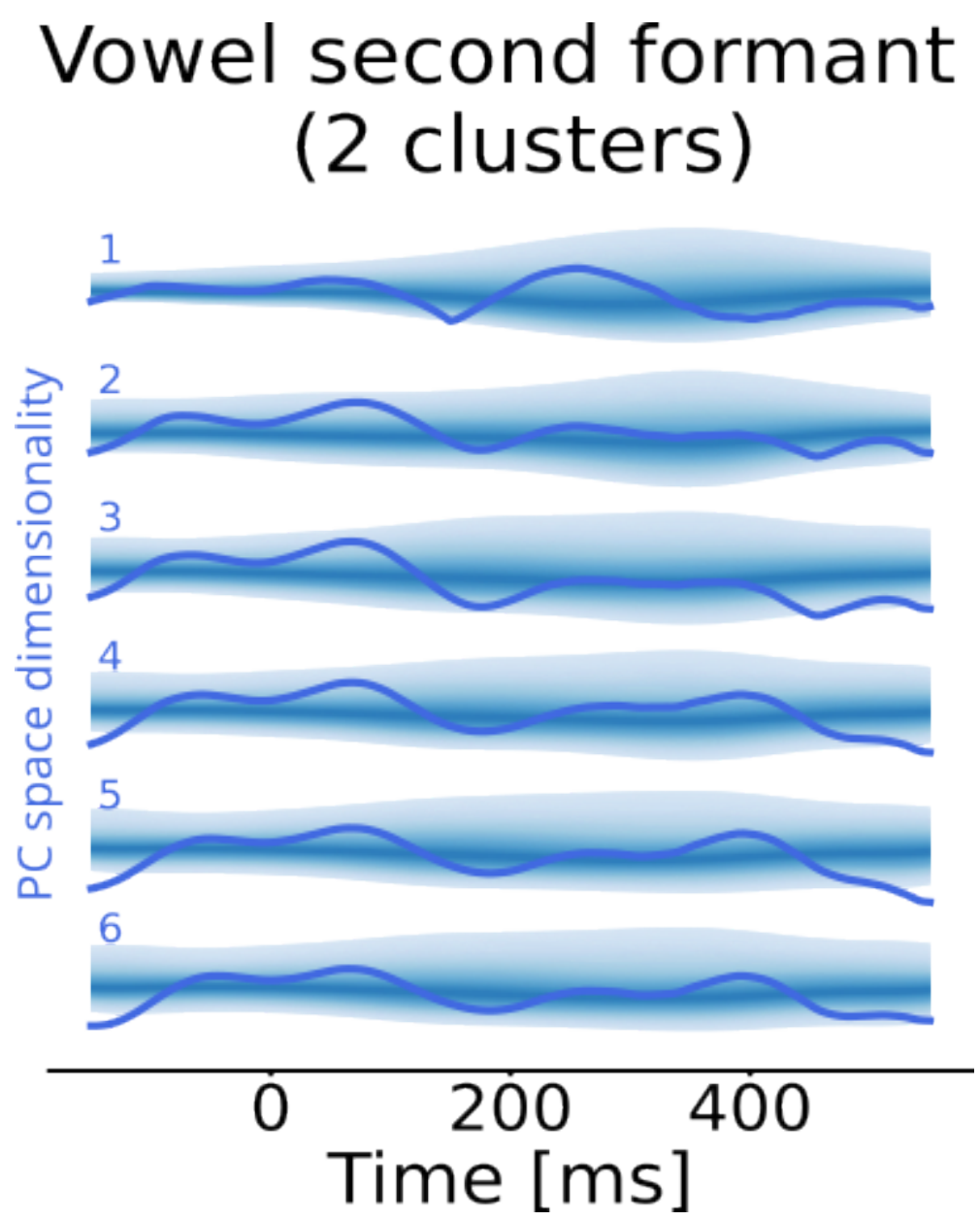

Supplementary Fig. 10 | Clustering for the vowel second formant group using only two phonetic features in Participant 1. The 95% confidence region of the surrogate (chance-level) distribution is shown with brighter shading for increasingly peripheral percentiles.

Linear discriminant analysis (LDA) classifier

We ran the LDA classifier for each of the 4 phonetic groups (vowel first formant, vowel second formant, consonant manner, consonant place). For each time point, we first ran an LDA classifier which is a Gaussian mixture model to compute the means of the multivariate normal distributions for each class. Then, we computed the average Euclidean distance between all class means and compared it against the distribution of 1000 surrogates. This is similar to the between-cluster distances from our clustering algorithm, with the difference that here the class mean (cluster centroid) is a parameter of the estimated multivariate distribution, and not computed directly by averaging class elements.

Replicating the clustering index analyses, at 200 ms we observed a significant separation of vowels into first formant categories (Supplementary Fig. 11a). Linear separators estimated at 0 and 200 ms are shown in Supplementary Fig. 11b. No peaks was observed for the vowel second formant (Supplementary Fig. 11c). LDA also indicated the two previously observed peaks for consonant manner (200 and 400 ms), however, here, they did not reach significance (Supplementary Fig. 11d). Finally, LDA revealed a significant peak for consonant place at 50 ms (Supplementary Fig. 11e) that was not observed before.

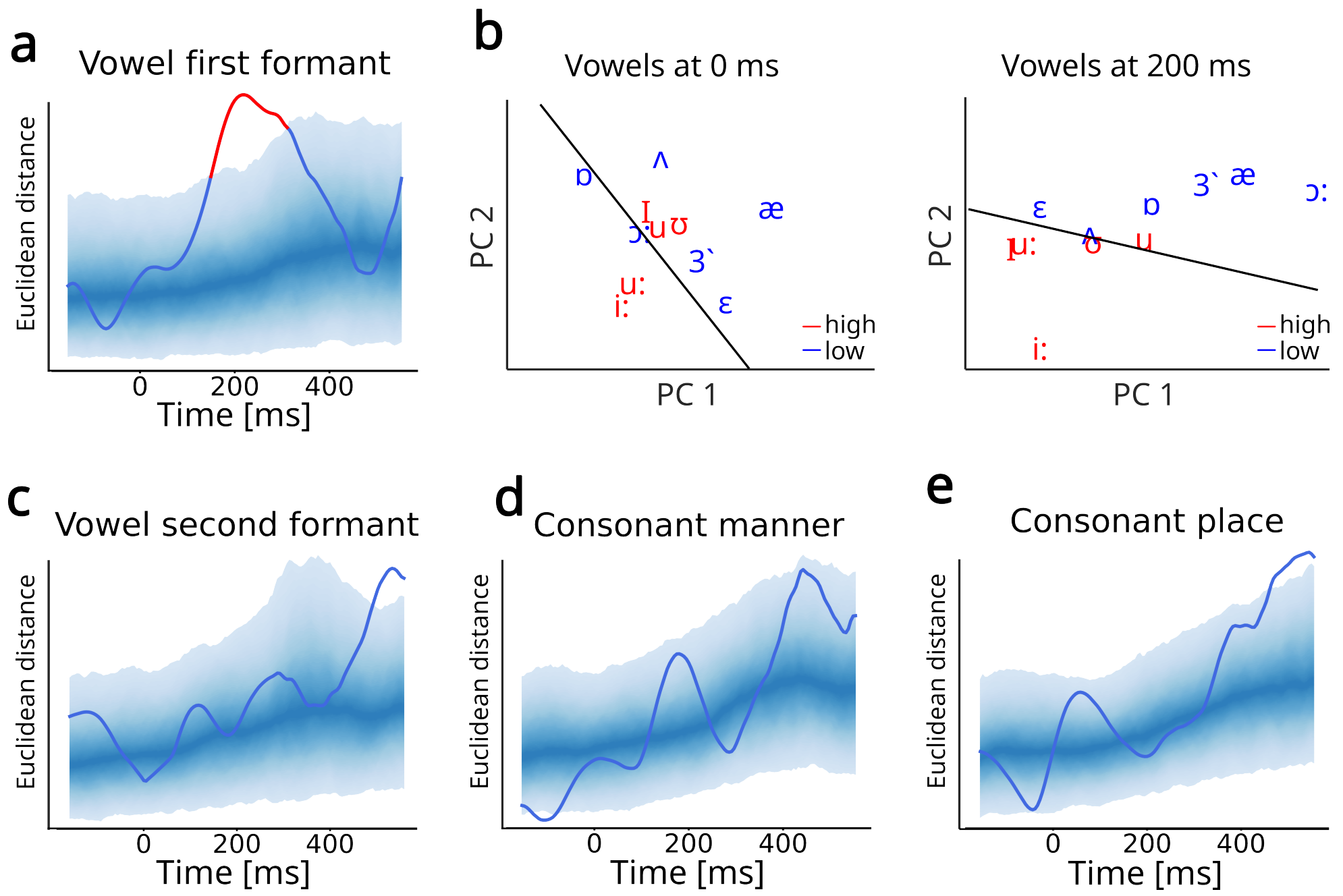

Supplementary Fig. 11 | **Linear discriminant analysis during the semantic categorization task in Participant 1. a.** Vowel first formant. The 95% confidence region of the surrogate (chance-level) distribution is shown with brighter shading for increasingly peripheral percentiles. Red segments indicate significant periods after multiple comparison correction (cluster-based test). **b.** Estimated linear separators for vowel first formant feature at 0 and 200 ms. **c** Vowel second formant. Shading and red segments are as in a. **d.** Consonant manner of articulation. Coloring as in a. **e.** Consonant place of articulation. Coloring as in a.

Rank regression

Since formants are actually ordered by their frequency values along the first and second formant axis, we additionally explored whether the actual ordering of formant frequency values was encoded in the low-dimensional space. To that aim, we ran a rank regression analysis, where a rank value (1-7) was assigned to each vowel, based on the standard IPA table. At each time point, the ranked order of vowels was correlated with their coordinates on the first three axes (PC1, PC2, and PC3), and compared against a distribution of 1000 surrogates.

We observed a significant ordering of vowels based on their first formant ranks on all three PCs (Fig. 12a). We performed the same regression for the second formant values and observed a significant correlation at about 0 ms, but only on PC1 (Supplementary Fig. 12b). As the two consonant groups are based on categorical and not continuous variables, rank regression analysis is not applicable.

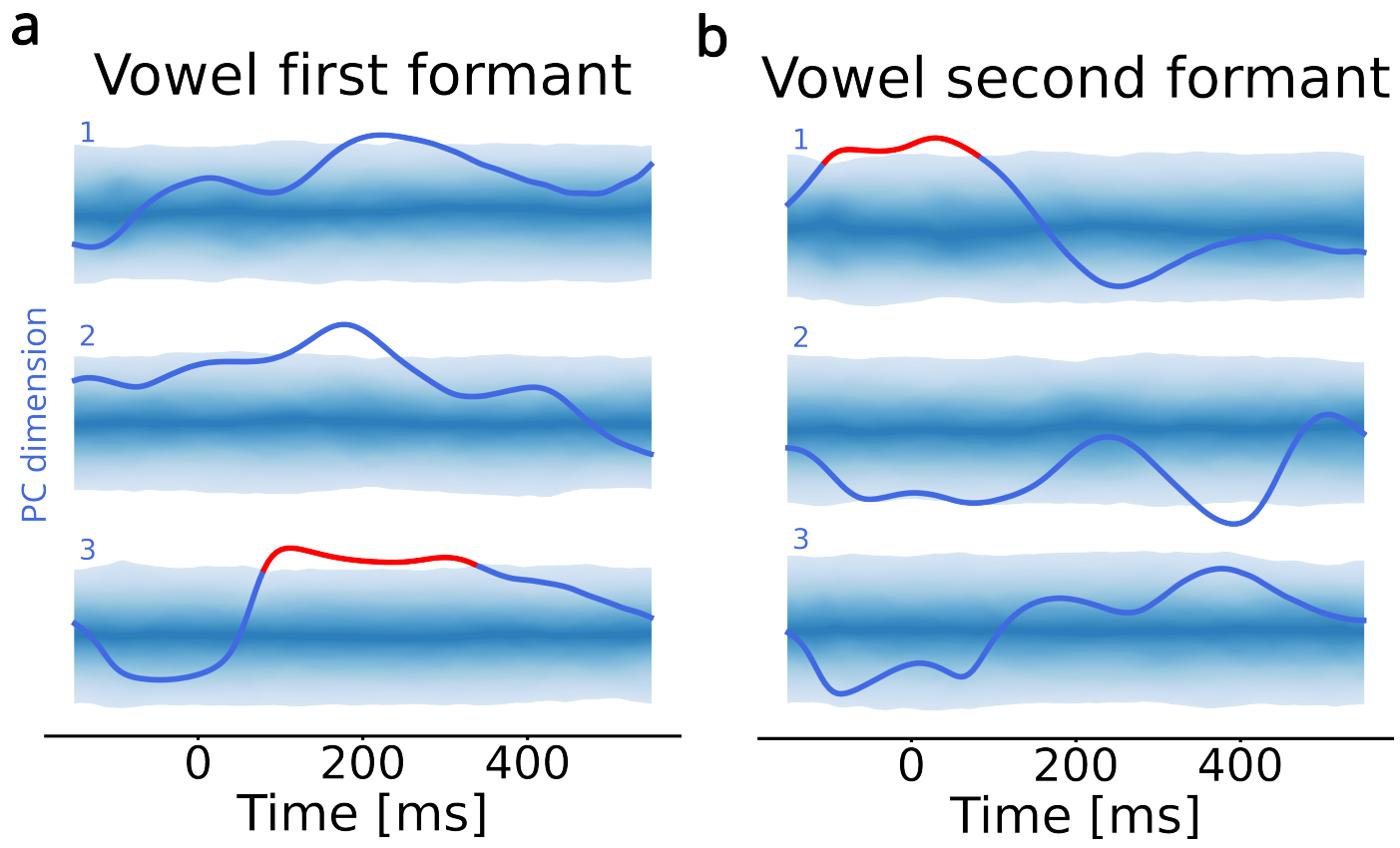

Supplementary Fig. 12 | **Rank regression during semantic categorization in Participant 1. a.** Correlation between vowel first formant ranks and coordinates on PC axes. The 95% confidence region of the surrogate (chance-level) distribution is shown with brighter shading for increasingly peripheral percentiles. Red segments indicate significant periods after multiple comparison correction (cluster-based test). Numbers indicate PC dimensions. **b.** Correlation between vowel second formant ranks and coordinates on PC axes. Coloring and numbering as in **a**.

K-means clustering

We used k-means clustering to investigate whether the same clustering of phonemes would emerge in a data-driven fashion. Our clustering results of the main text necessitate assigning a priori each of the N phonemes to a cluster (e.g., for the vowel first formant feature see Supplementary Fig. 13a), and the clustering index is then quantified based on the Euclidean distances of those a priori chosen clusters. On the contrary, k-means clustering is an unsupervised method that partitions all N phonemes into k clusters based on their proximity in PC space. Thus, for each time point, we first ran k-means clustering 1000 times, as the clustering results might change based on the algorithm's random initialization. Then, we computed an average N-by-N attribution matrix that indicated how often each of the N phonemes was clustered together (Supplementary Fig. 13b). Finally, we correlated the resulting k-means attribution matrix with the attribution matrix of the actual, linguistically-based clusters, and compared the correlation value against the distribution of correlation values for the 1000 surrogates at each time point (Supplementary Fig. 13c-f).

Replicating previous clustering results for the vowel first formant group, this analysis revealed a significant period at about 200 ms during which data-driven clusters overlapped with the linguistic ones (Supplementary Fig. 13c). This analysis further revealed a significant peak at 75 ms for vowel second formant that was not present before (Supplementary Fig. 13d). The 200-ms peak was also observed again for the consonant manner group, with an additional peak at about 0 ms, and no significant peak at 400 ms (Supplementary Fig. 13e). No significant peaks were observed for consonant place (Supplementary Fig. 13f).

Despite its advantageous data-driven perspective, there are also some important drawbacks of this method, that can be observed in the shape of the correlation curve for the vowel first formant group (Supplementary Fig. 13c). Namely, at 200 ms there is a

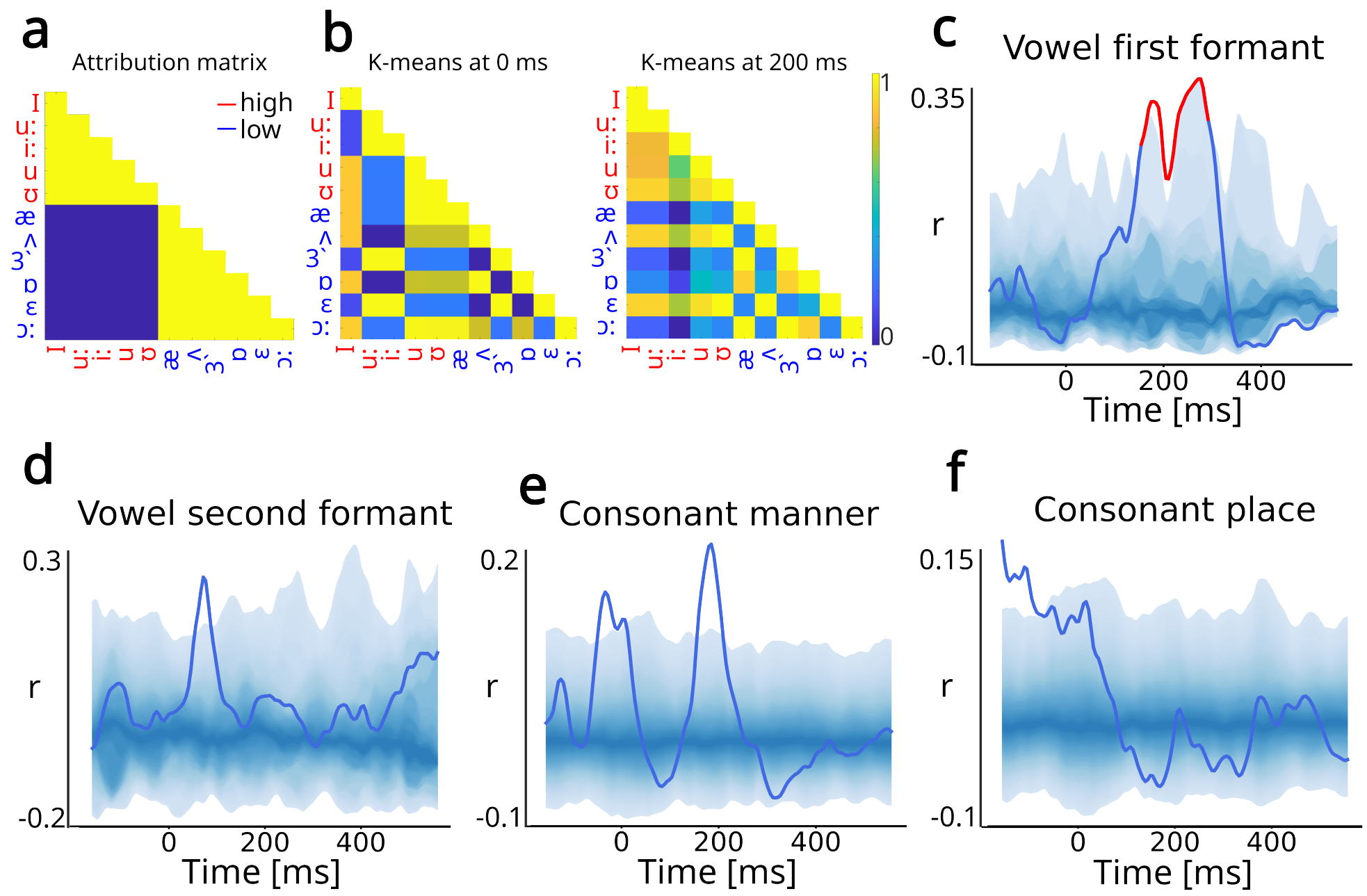

Supplementary Fig. 13 | K-means clustering during semantic categorization in Participant 1. a. Attribution matrix for the vowel first formant feature. **b.** K-means matrices for the vowel first formant feature at 0 and 200 ms. **c.** Correlation between vowel first formant attribution matrix (**a**) and corresponding k-means matrices (**b**) at each time point. The 95% confidence region of the surrogate (chance-level) distribution is shown with brighter shading for increasingly peripheral percentiles. Red segments indicate significant periods after multiple comparison correction (cluster-based test). **d.** Correlation between vowel second formant and corresponding k-means attribution matrices at each time point. Coloring as in **c**. **e.** Correlation between consonant manner and corresponding k-means matrices at each time point. Coloring as in **c**. **f.** Correlation between consonant place and corresponding k-means matrices at each time point. Coloring as in **c**.

decrease in the correlation value (although still significant), because at that time point vowels /ʌ/ and /ɛ/, which belong to the 'low' group, are closer in space to the 'high' group (compare the 200-ms insets on Fig. 2e and Supplementary Fig. 13b). However, it is apparent that, despite their spatial proximity to the 'high' group, vowels /ʌ/ and /ɛ/ can be attributed to the 'low' group because the two clusters can be linearly separated right next to the positions of these two vowels in space (Supplementary Fig. 11b).

Summary of control analyses

Supplementary Table 5 summarizes all peaks observed across different analyses for all 4 groups. Although some analyses sporadically revealed significant peaks for vowel second formant (k-means) and consonant place (LDA) groups, the peaks that were consistent across all analyses are the same peaks as observed in our initial clustering analysis: 200 ms for vowel first formant and consonant manner, and 400 ms for consonant manner alone.

Supplementary Table 5 | Summary of the significant window locations during semantic categorization identified through control analyses. Bold entries indicate peaks that were consistent across analyses. Entries in brackets indicate existing peaks that did not cross the significance threshold. Entries in red indicate peaks surviving multiple comparison correction (cluster-based test). All values are in ms.

**Phonetic feature group**	**Clustering**	**LDA**	**Rank regression**	**K-means**
Vowel first formant	**200**	**200**	**200**	**200**
Vowel second formant			0	75
Consonant manner	**200, 400**	**(200, 400)**	not applicable	0, **200**
Consonant place		450	not applicable	

Clustering control analyses for natural speech perception

We performed the same control analyses for natural speech perception: generalization of clustering for up to 6-dimensional PC spaces for phonetic (Supplementary Figs. 14 and 15) and semantic features (Supplementary Fig. 16). As for the semantic task, for phonetic features we also performed LDA (Supplementary Fig. 17), rank regression (Supplementary Fig. 18), and correlation with K-means attribution (Supplementary Fig. 19). We then summarized the significant peaks observed during natural speech perception across all analyses. (Supplementary Table 6).

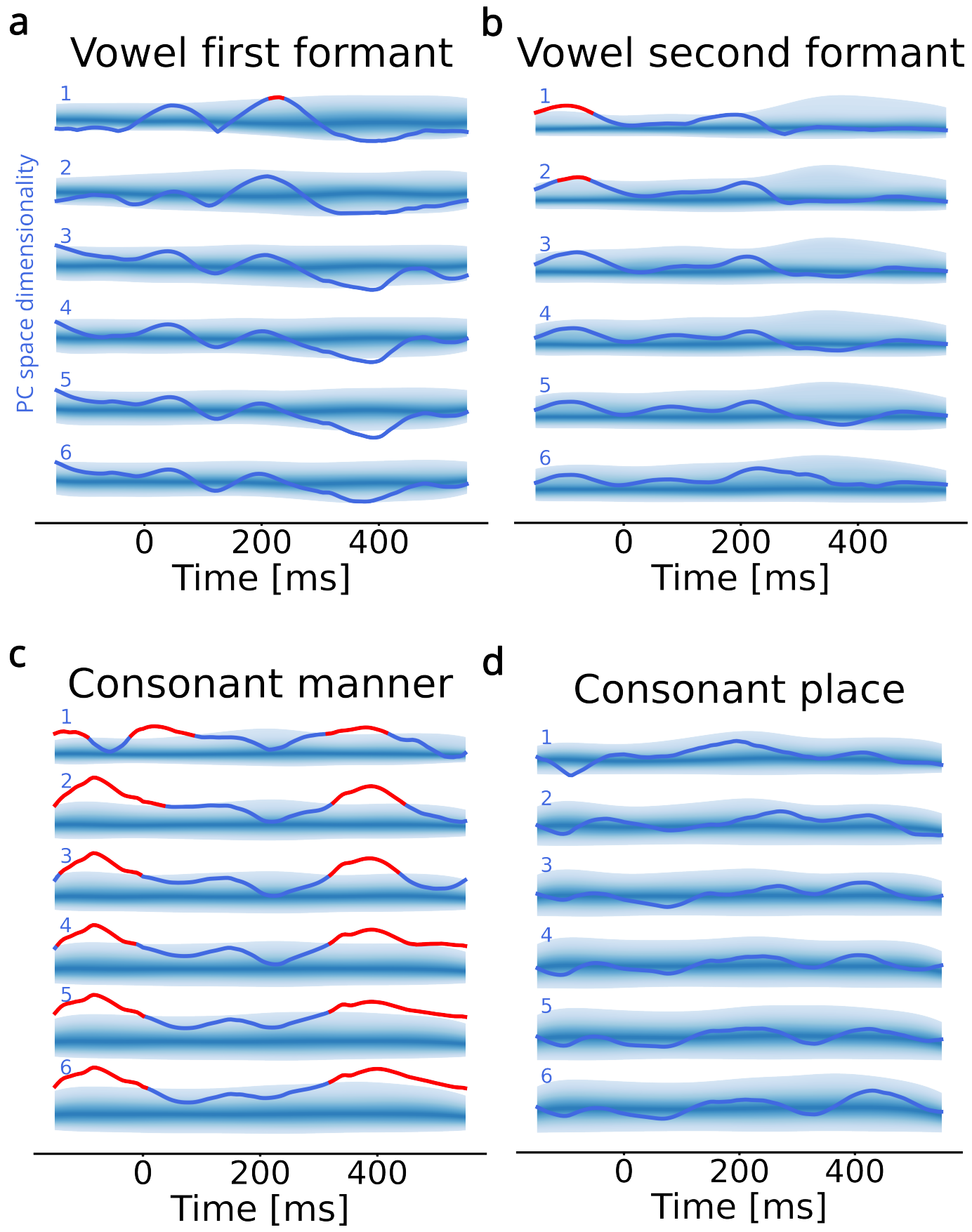

Supplementary Fig. 14 | Clustering index across dimensions during natural speech perception in Participant 1. a. Clustering index for vowels grouped by the first formant (high and low tongue position) up to 6 PC dimensions. The 95% confidence region of the surrogate (chance-level) distribution is shown with brighter shading for increasingly peripheral percentiles. Numbers indicate the dimensionality of the PC space. **b.** Clustering index for vowels grouped by the second formant up to six PC dimensions. Coloring and numbering as in a. **c.** Clustering index for consonants grouped by the manner of articulation up to six PC dimensions. Shading and numbering as in a. **d.** Clustering index for consonants grouped by the place of articulation up to six PC dimensions. Coloring and numbering as in a.

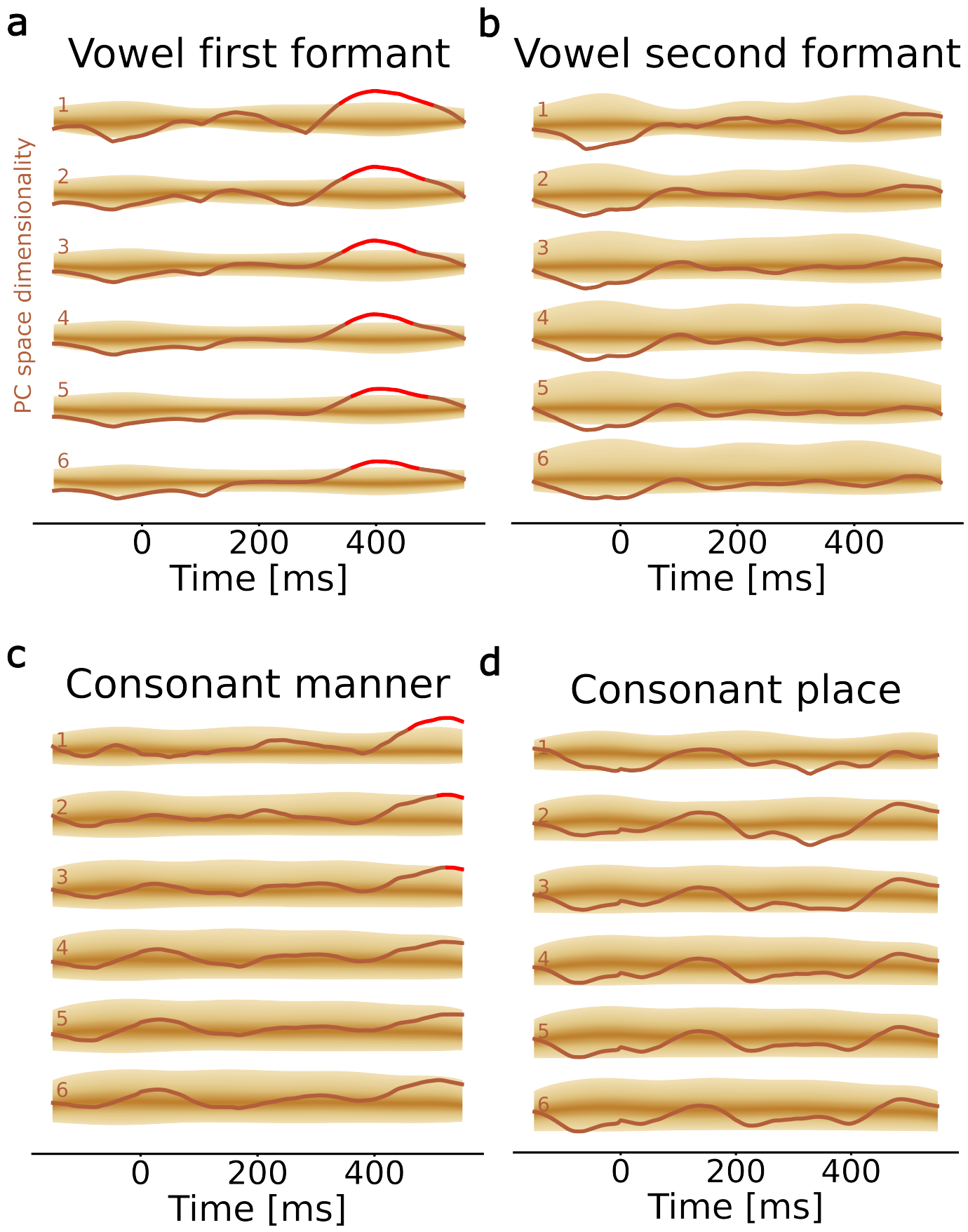

Supplementary Fig. 15 | **Clustering index across dimensions during natural speech perception in Participant 3. a.** Clustering index for vowels grouped by the first formant (high and low tongue position) up to 6 PC dimensions. The 95% confidence region of the surrogate (chance-level) distribution is shown with brighter shading for increasingly peripheral percentiles. Numbers indicate the dimensionality of the PC space. **b.** Clustering index for vowels grouped by the second formant up to six PC dimensions. Coloring and numbering as in a. **c.** Clustering index for consonants grouped by the manner of articulation up to six PC dimensions. Shading and numbering as in a. **d.** Clustering index for consonants grouped by the place of articulation up to six PC dimensions. Coloring and numbering as in a.

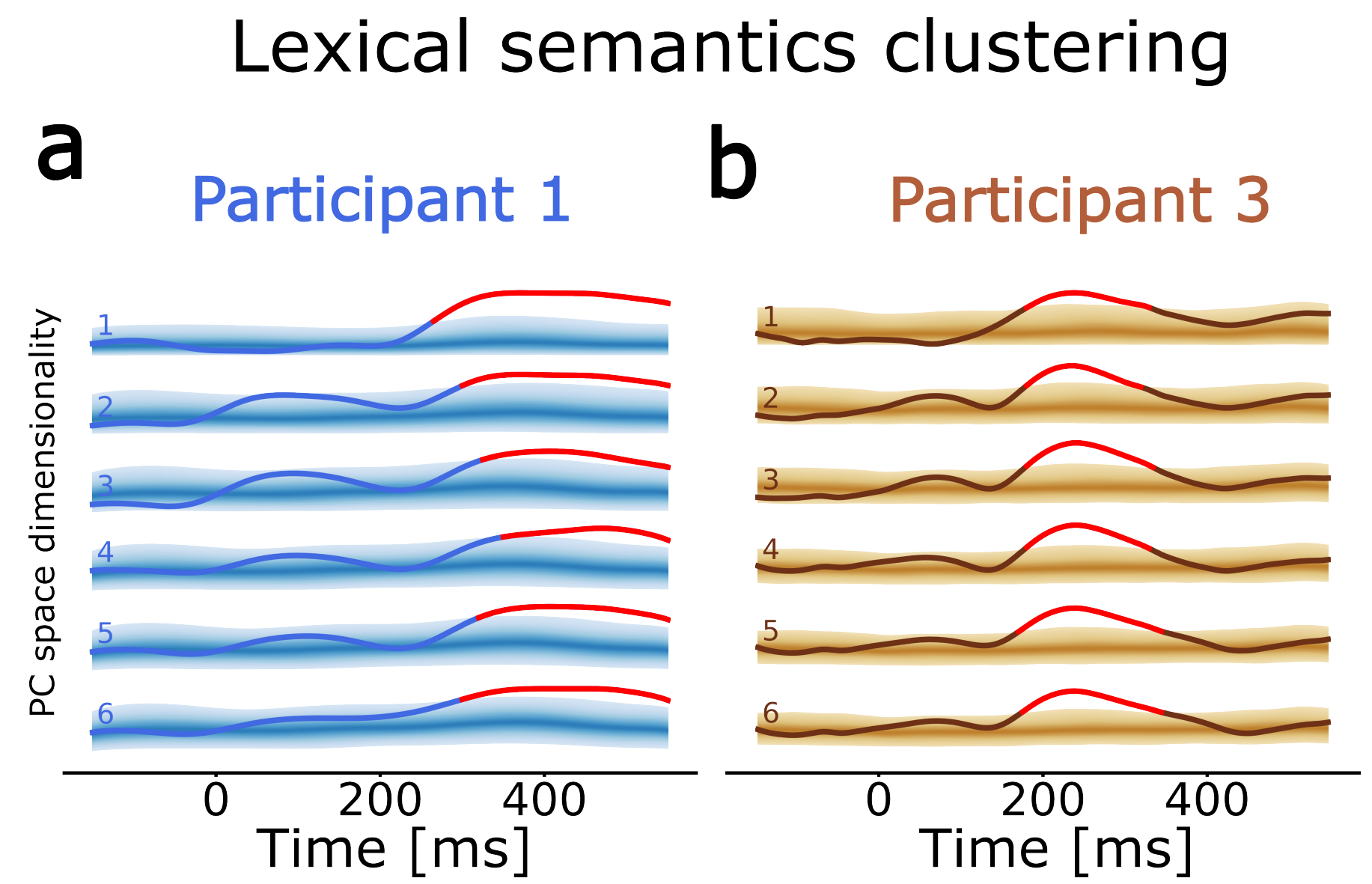

Supplementary Fig. 16 | Clustering of semantic features across dimensions during natural speech perception. a. Clustering index for lexical semantics feature in Participant 1. The 95% confidence region of the surrogate (chance-level) distribution is shown with brighter shading for increasingly peripheral percentiles. Red segments indicate significant periods after multiple comparison correction (cluster-based test). Numbers indicate the dimensionality of the PC space. **b.** Clustering index for lexical semantics feature in Participant 3.

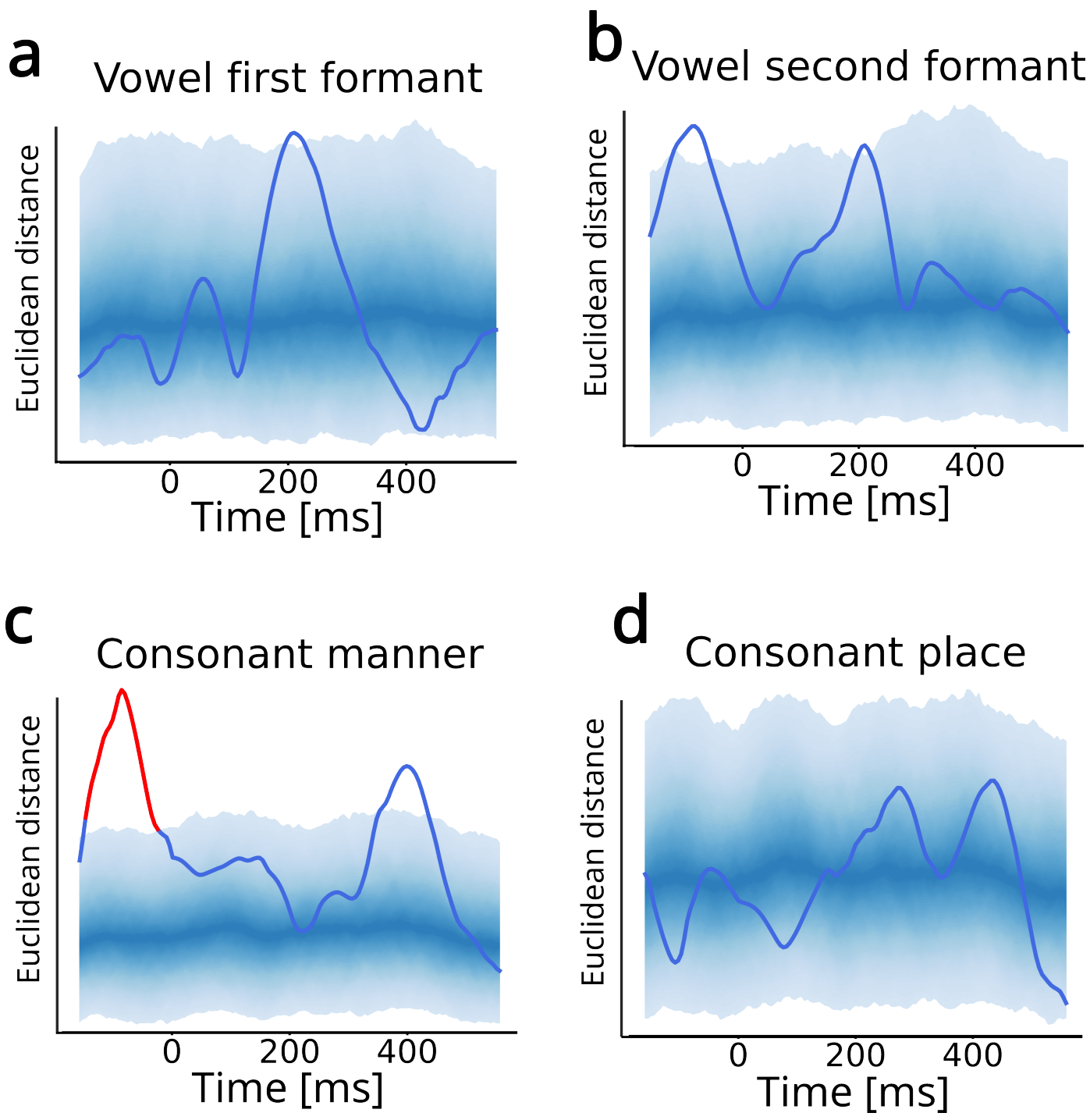

Supplementary Fig. 17 | Linear discriminant analysis during natural speech perception in Participant 1. a. Vowel first formant. The 95% confidence region of the surrogate (chance-level) distribution is shown with brighter shading for increasingly peripheral percentiles. Red segments indicate significant periods after multiple comparison correction (cluster-based test). **b.** Vowel second formant. Coloring as in **a**. **c.** Consonant manner of articulation. Coloring as in **a**. **d.** Consonant place of articulation. Coloring as in **a**.

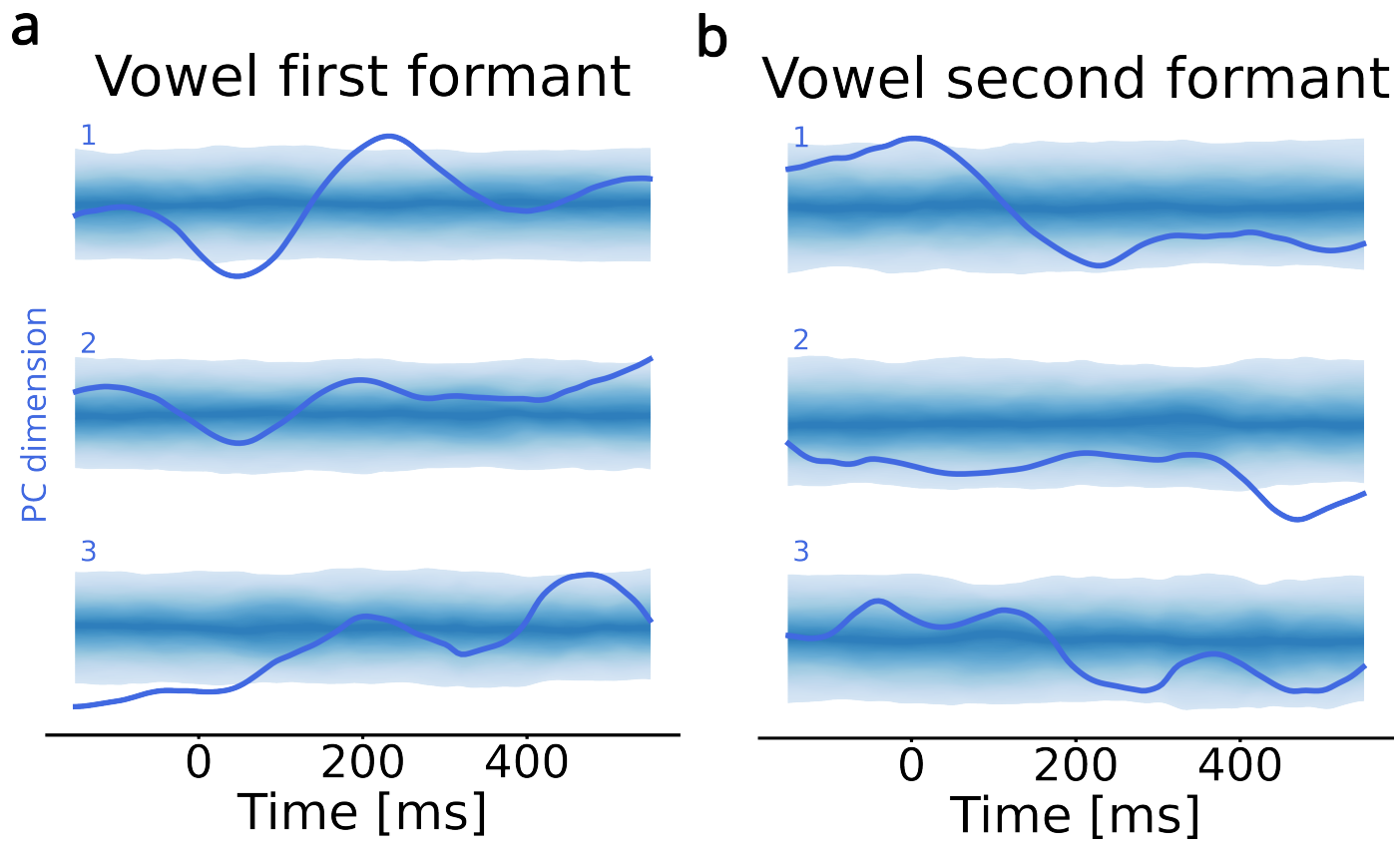

Supplementary Fig. 18 | Rank regression during natural speech perception in Participant 1. a. Correlation between vowel first formant ranks and coordinates on PC axes. The 95% confidence region of the surrogate (chance-level) distribution is shown with brighter shading for increasingly peripheral percentiles. Numbers indicate PC dimensions. **b.** Correlation between vowel second formant ranks and coordinates on PC axes. Coloring and numbering as in **a**.

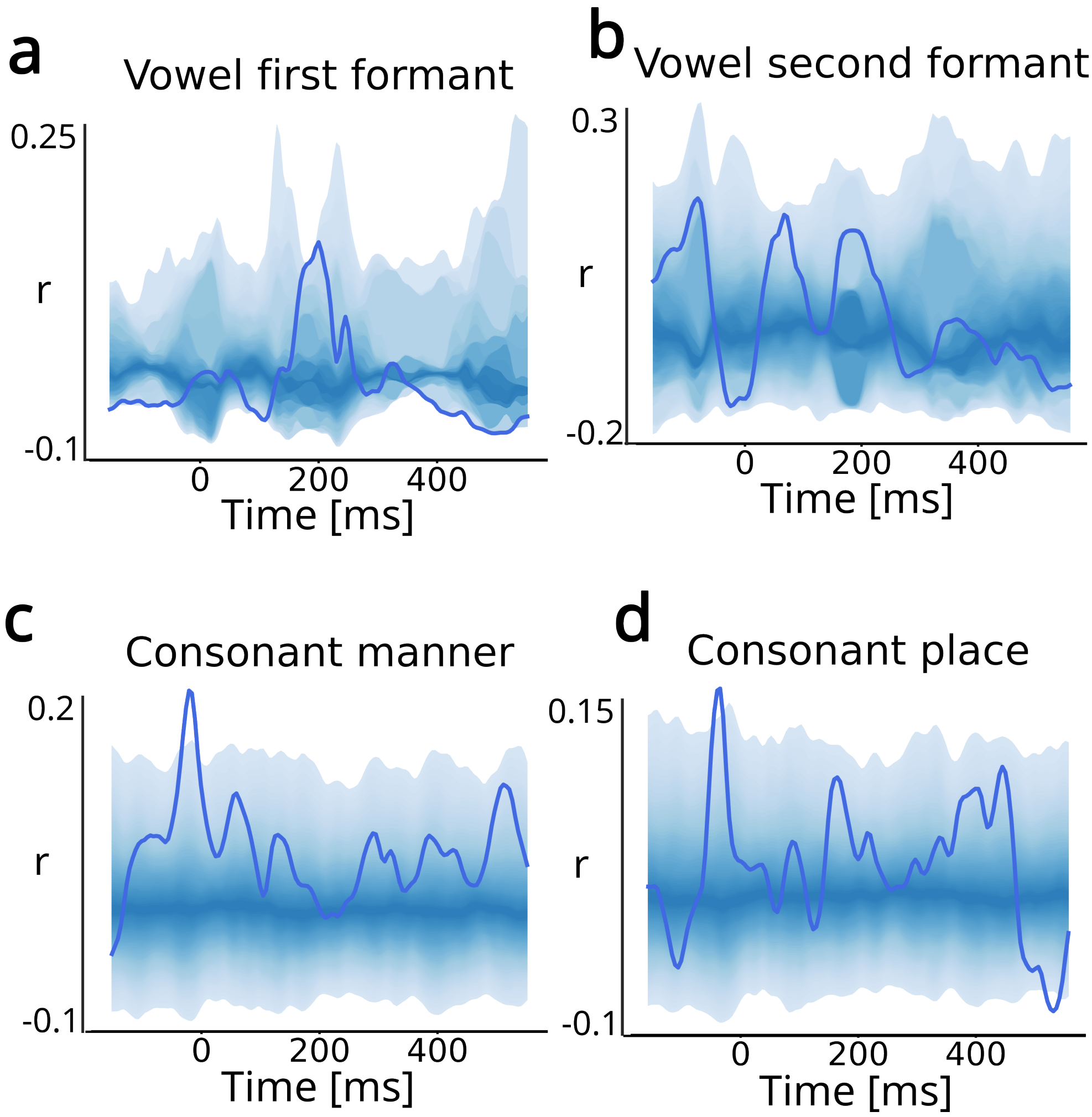

Supplementary Fig. 19 | **K-means clustering during natural speech perception in Participant 1. a.** Correlation between vowel first formant and corresponding k-means attribution matrices at each time point. The 95% confidence region of the surrogate (chance-level) distribution is shown with brighter shading for increasingly peripheral percentiles. **b.** Correlation between vowel second formant and corresponding k-means attribution matrices at each time point. Coloring as in **a**. **c.** Correlation between consonant manner and corresponding k-means attribution matrices at each time point. Coloring as in **a**. **d.** Correlation between consonant place and corresponding k-means attribution matrices at each time point. Coloring as in **a**.

Supplementary Table 6 | Summary of the significant window locations during natural speech perception identified through control analyses. Bold entries indicate peaks that were consistent across analyses. Entries in brackets indicate existing peaks that did not cross the significance threshold. Entries in red indicate peaks surviving multiple comparison correction (cluster-based test). All values are in ms.

Phonetic feature group	Clustering	LDA	Rank regression	K-means
Vowel first formant	**(200)**	**200**	**200**	**200**
Vowel second formant	**-100, (200)**	**-100, 200**	0	
Consonant manner	**-100, 400**	**-100, 400**	not applicable	-25
Consonant place			not applicable	-25

Geometrical alignment of phonetic and lexico-semantic neural manifolds

To further characterize the relationship between phonetic and semantic representations, we quantified the geometry of the low-dimensional population spaces obtained from the phonetic and semantic mTRF models. The goal of this analysis was to test whether phonetic and semantic responses occupied independent neural subspaces, shared the same low-dimensional space, or showed partial overlap.

For each participant and condition, we extracted the first three principal components from the phonetic and semantic response spaces. Each principal component corresponds to a PCA eigenvector, or population axis, defined by one weight per unit. Thus, the first three phonetic eigenvectors defined a three-dimensional phonetic subspace in unit space, and the first three semantic eigenvectors defined a three-dimensional semantic subspace in the same unit space.

We first quantified alignment between individual phonetic and semantic eigenvectors. Specifically, we computed the absolute dot product between each of the first three phonetic PC eigenvectors and each of the first three semantic PC eigenvectors. This produced a 3 x 3 PC-by-PC overlap matrix. Values close to 1 indicate that the corresponding phonetic and semantic population axes are strongly aligned, whereas values close to 0 indicate near-orthogonality. Because the sign of each PCA axis is arbitrary, we used the absolute value of the dot product.

We then quantified the overlap between the full three-dimensional phonetic and semantic subspaces using principal angles. To compute these angles, we formed a 3 x 3 matrix containing all pairwise dot products between the first three phonetic eigenvectors and the first three semantic eigenvectors. We then applied singular value decomposition to this matrix. The singular values are the cosines of the principal angles between the two subspaces, so the angles were obtained by taking the inverse cosine of the singular values. The first principal angle corresponds to the best-aligned population axis shared between the two subspaces, the second angle corresponds to the next-best independent alignment, and the third angle corresponds to the final independent alignment. Small principal angles indicate strong subspace overlap, whereas angles near 90 degrees indicate near-orthogonality. We summarized the overall subspace overlap as the mean squared cosine of the three principal angles, where values near 1 indicate strong overlap and values near 0 indicate near-orthogonal subspaces.

Finally, we performed a descriptive analysis of point-cloud geometry. This analysis used the PCA score matrices, i.e. the coordinates of observations in the first three

PCs. The phonetic and semantic spaces were each represented as observation-by-PC matrices. We centered both pointclouds by subtracting their column means and then tested how well increasingly flexible transformations could map the phonetic coordinates onto the semantic coordinates. For translation only, we shifted the phonetic point cloud to the semantic mean. For uniform scaling, we estimated the single scalar multiplier that minimized the squared error between the scaled phonetic coordinates and the semantic coordinates. For rotation only, we estimated the orthogonal matrix that minimized the squared error between the phonetic and semantic point clouds, using singular value decomposition of the cross-covariance matrix, while constraining the determinant to be +1. For rotation/reflection, we used the same orthogonal Procrustes solution but allowed the determinant to be either +1 or -1. For rotation/reflection plus global scaling, we first estimated the best orthogonal transform and then estimated a single global scale factor. For the full affine model, we solved a linear regression mapping the phonetic coordinates to the semantic coordinates, allowing translation, rotation, reflection, non-uniform scaling, compression, expansion, and shear-like transformations.

For each transformation, we computed an R-squared value comparing the predicted semantic coordinates to the observed semantic coordinates. Negative R-squared values indicate that the transformation predicted the semantic coordinates worse than simply predicting their mean. For the full affine transform, we also computed the singular values of the affine matrix. These singular values quantify scaling along the main axes of the affine transformation: similar singular values indicate approximately uniform scaling, whereas strongly unequal singular values indicate dimension-specific stretching or compression. We also computed the anisotropy ratio, defined as the largest singular value divided by the smallest singular value, and a non-similarity index quantifying how far the affine transform was from a simple rotation/reflection plus uniform scaling.

Across the four datasets, the most consistent result was that phonetic and semantic spaces were not orthogonal. Each participant and condition showed at least one relatively small principal angle, indicating at least one shared population dimension between phonetic and semantic spaces. Mean subspace overlap was 0.59 in Participant 1 task, 0.95 in Participant 2 task, 0.53 in Participant 1 speech, and 0.60 in Participant 3 speech.

Participant 1 showed a similar degree of phonetic–semantic overlap in the task and speech conditions. The mean overlap was 0.59 in task and 0.53 in speech, and the angle structure was similar across the two settings: both had one clearly shared population dimension, one intermediate dimension, and one more separated dimension. This suggests that, within Participant 1, phonetic and semantic representations showed a comparable degree of partial overlap across task and speech-listening contexts.

The two speech participants also showed a similar overall pattern. Participant 1 speech and Participant 3 speech both showed partial phonetic–semantic overlap, with mean overlaps of 0.53 and 0.60, respectively. The precise dimensional structure differed: Participant 1 speech showed one strongly shared dimension and two more distinct dimensions, whereas Participant 3 speech showed two shared dimensions and one more clearly separate dimension. Nevertheless, both speech participants showed

measurable phonetic–semantic subspace overlap, indicating that this pattern was not unique to one participant.

Participant 2 task was the main outlier, showing much stronger overlap than the other datasets. Because Participant 2 had sparse task responses, this result may partly reflect the dominance ofa small number of responsive units in both phonetic and semantic PCA spaces. Therefore, we interpret Participant 2 as showing strong overlap of the dominant low-dimensional axes, while avoiding the stronger claim that this reflects a broadly distributed shared manifold.

Overall, the geometry analysis indicates that phonetic and semantic representations are not implemented in fully separate neural subspaces. Instead, they occupy partially overlapping population spaces. At the same time, except for Participant 2 in the task condition, the relationship between phonetic and semantic point clouds was not well captured by a simple rotation. In most datasets, affine transformations provided better fits and showed unequal singular values, indicating dimension-specific stretching or compression. Thus, the geometry of phonetic and semantic coding is best described as partial overlap with participant- and condition-specific transformations, rather than complete separation or complete identity of the two representational spaces.

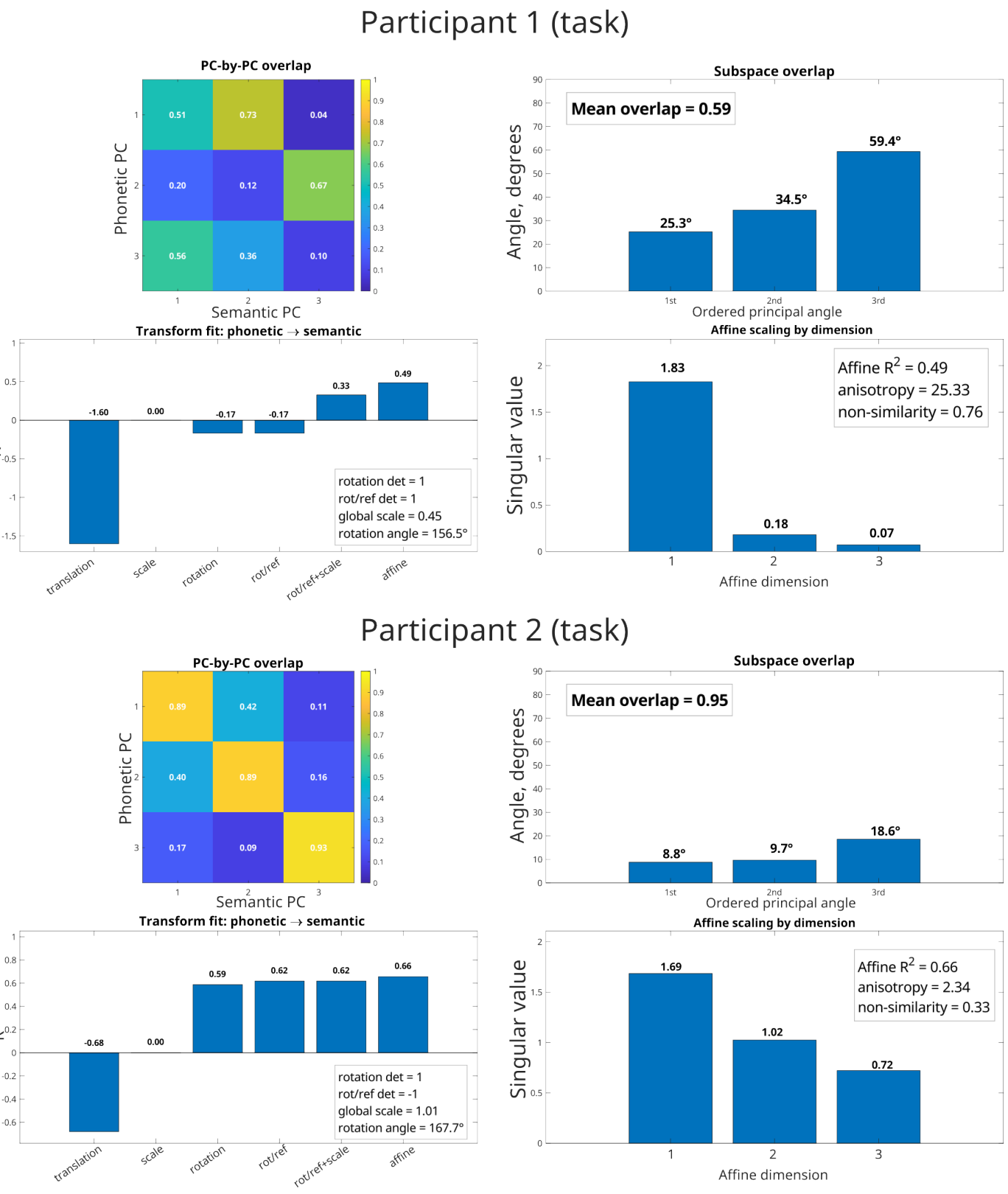

Supplementary Fig. 20 Geometry of phonetic and semantic manifolds during the semantic categorization task. For each task participant, the first panel shows the absolute PC-by-PC overlap between the first three phonetic and semantic PC eigenvectors. Brighter values indicate stronger alignment between individual population axes. The second panel shows the principal angles between the three-dimensional phonetic and semantic subspaces. Smaller angles indicate stronger subspace overlap. The third panel shows how well the phonetic point cloud could be transformed to predict the semantic point cloud using increasingly flexible transformations: translation, uniform scaling, rotation, rotation/reflection, rotation/reflection plus global scaling, and full affine mapping. The fourth panel shows the singular values of the full affine transformation,indicating dimension-specific scaling or compression. Participant 1 showed partial phonetic– semantic overlap with mixed PC axes and a better fit for the affine transform than for simpler transformations. Participant 2 showed the strongest phonetic–semantic overlap, with small principal angles and a transformation profile consistent with a largely orthogonal mapping between spaces. This Participant 2 result should be interpreted cautiously because Participant 2 had sparser task responses than Participant 1, and only a small number of units contributed strongly to the low-dimensional task structure. Therefore, the high phonetic–semantic overlap in Participant 2 may partly reflect the fact that both phonetic and semantic PCA spaces were dominated by the same small set of responsive units, rather than indicating a broadly distributed shared phonetic–semantic manifold (Supplementary figures 1 and 3).

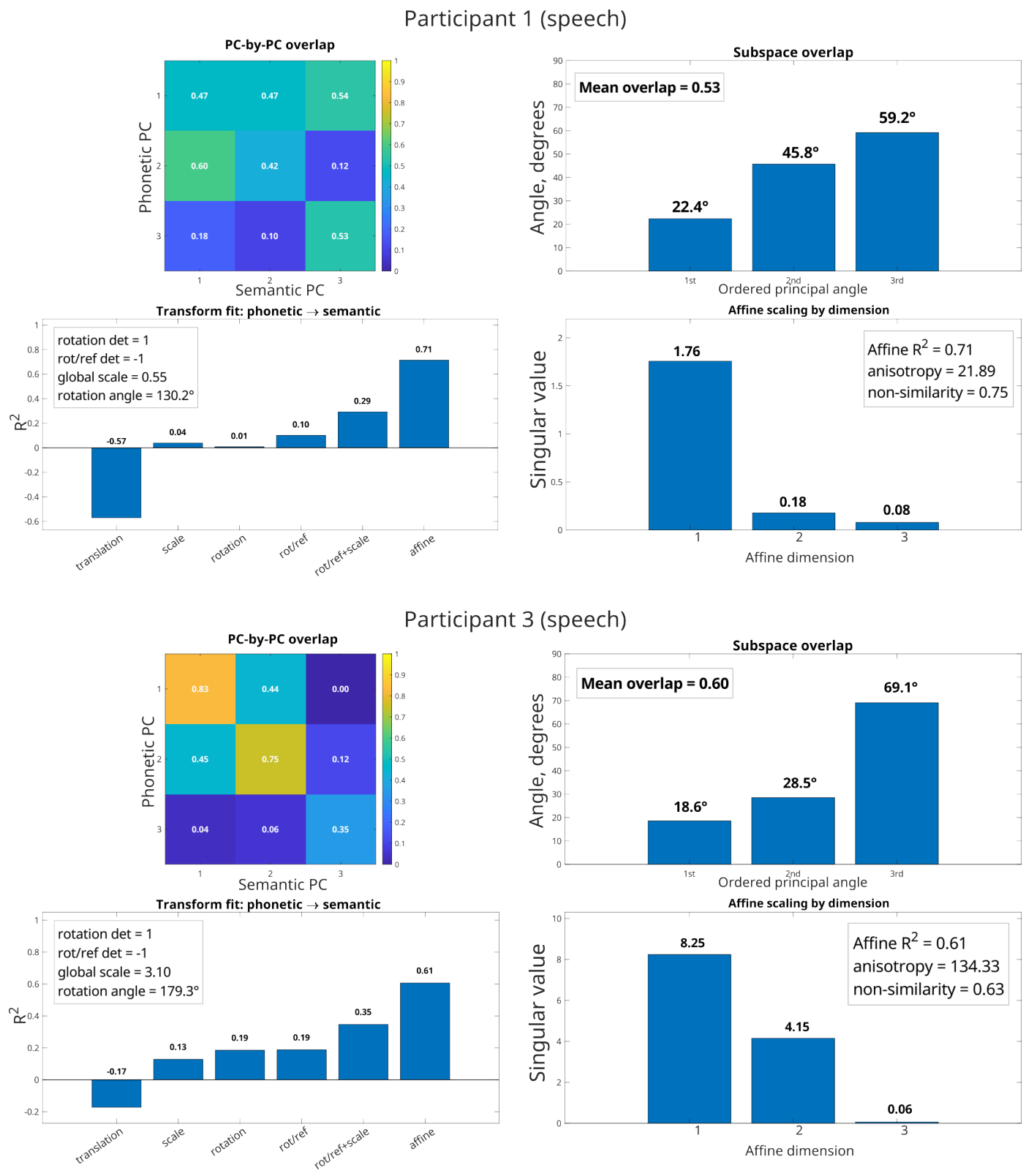

Supplementary Fig. 21 Geometry of phonetic and semantic spaces during natural speech perception The same geometry analysis was applied to the speech-listening data. Participant 1 showed partial overlap between phonetic and semantic spaces, with one relatively shared dimension and two more distinct dimensions. Participant 3 also showed partial overlap, with stronger alignment in the first two dimensions and a more separate third dimension. In both speech participants, full affine transformations fit the point-cloud relationship better than simple rotations, indicating that phonetic and semantic spaces shared some population structure but differed in dimension-specific scaling

Top-down semantic and phonetic alignment

Control for word-level lexical and phonological covariates

To test whether the phonetic–semantic alignment shown in Fig. 4b could be explained by word-level variables related to lexical access, lexical predictability, or phonological word form, we performed an additional control analysis including four covariates: word frequency, word duration, GPT-2 surprisal, and phonotactic probability.

Word frequency was computed for each transcribed word using the `wordfreq` Python package, which returns English Zipf-scale lexical frequency estimates. Higher values therefore correspond to more frequent words in English.

Word duration was computed directly from the manual word annotations as the difference between word offset and word onset.

GPT-2 surprisal was computed using the pretrained GPT-2 base model (124M parameters). For each word token in the natural-speech transcript, we constructed an input sequence consisting of the target word preceded by up to the previous ten words from the same sentence or audio segment. The target word was always placed as the final word of the input sequence. Surprisal was computed as the negative summed log probability of the GPT-2 subword tokens corresponding to the target word, conditioned on the preceding context. For one-word utterances, a beginning-of-text token was prepended so that the probability of the word could still be estimated. This produced one surprisal value for each word token.

Phonotactic probability was computed from the CMU Pronouncing Dictionary. For each word, we used the first available dictionary pronunciation, removed lexical stress markers from phonemes, and modeled the pronunciation as a sequence of phoneme bigrams including start and end boundaries. Phoneme bigram probabilities were estimated from the full CMU dictionary with add-one smoothing. The phonotactic-probability value for each word was defined as the average log probability of its phoneme bigram sequence. Words absent from the dictionary were assigned the mean value of the regressor after z-scoring, corresponding to a neutral value in the mTRF model.

All four covariates were added as word-level regressors at the corresponding word onset times. Before inclusion in the mTRF model, each regressor was z-scored across word tokens. We first added these covariates to the baseline model and refit the lexical-semantic kernels used for the phonetic–semantic alignment analysis. The alignment between the phoneme-order and lexical-semantic kernels was preserved, indicating that the alignment reported in Fig. 4b cannot be explained by word frequency, word duration, GPT-2 surprisal, or phonotactic probability.

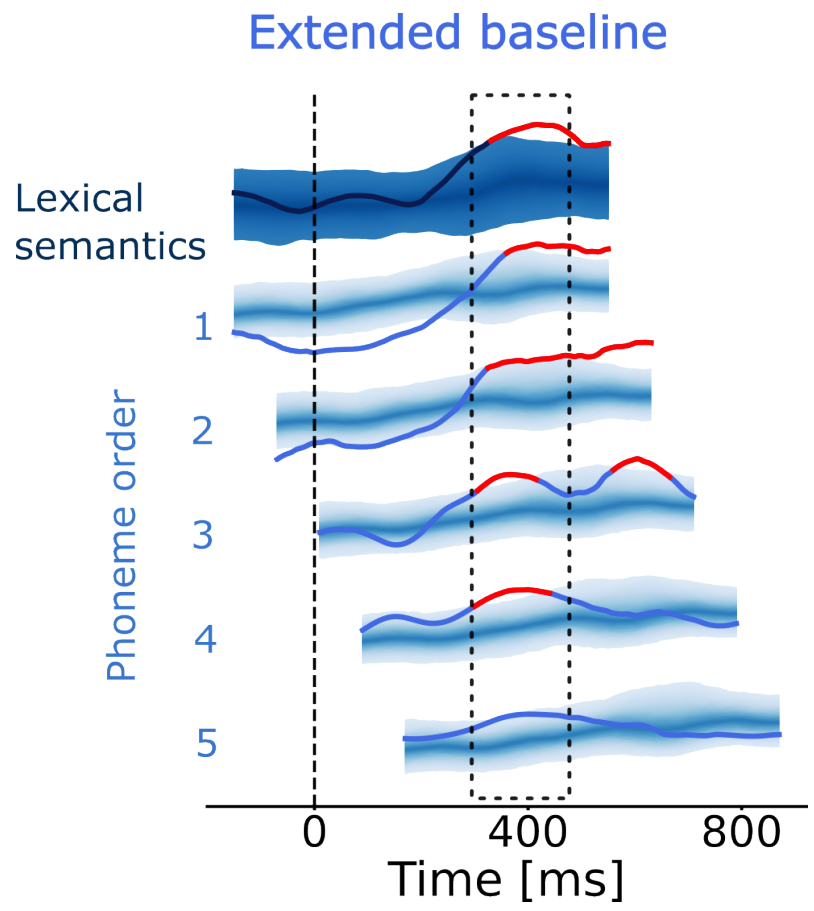

Supplementary Fig. 22 Phonetic–semantic alignment after controlling for word-level lexical and phonological covariates. The lexical-semantic kernels were refit after adding word frequency, word duration, GPT-2 surprisal, and phonotactic probability to the baseline model. The alignment analysis was then repeated as in main Fig. 4b. The same phonetic–semantic alignment was preserved after including these additional word-level covariates, indicating that the semantic-aligned peak is not explained by lexical frequency, word duration, language-model surprisal, or phonotactic probability.

We also fitted the four control covariates directly using the same baseline model used for the other encoding analyses, which included word-onset and acoustic-edge features (Fig. 2c). This analysis tested whether the four covariates were themselves encoded in the neuronal population. Among the four regressors, we observed a significant encoding period only for word duration. Thus, word duration was represented in the same neuronal population, but accounting for it did not alter the phonetic–semantic alignment.

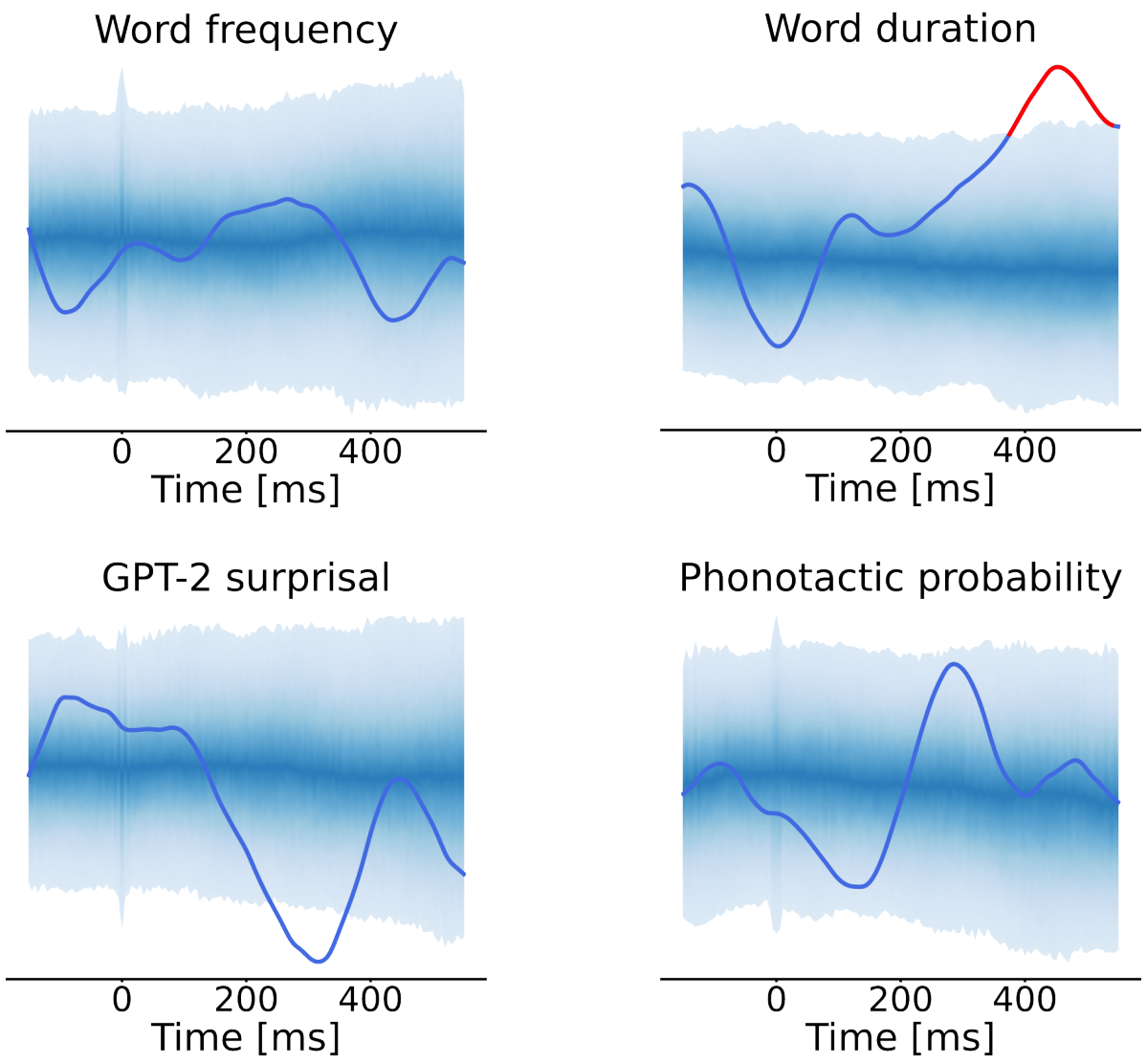

Supplementary Fig. 23 Encoding of word-level lexical and phonological control covariates. Kernels for word frequency, word duration, GPT-2 surprisal, and phonotactic probability were fitted using the same baseline model used for the other encoding analyses, containing word-onset and acoustic-edge features. Among these four covariates, a significant encoding period was observed only for word duration.

Together, these analyses show that the phonetic–semantic alignment is preserved after controlling for lexical frequency, word duration, GPT-2 surprisal, and phonotactic probability. The alignment therefore cannot be explained by general word-onset-locked lexical access, word predictability, word duration, or phonological word-form statistics.

Additional analyses for the top-down alignment in Participant 1

As an additional confirmation of the 400 ms top-down process for Participant 1, we extracted the broadband high-frequency activity (BHA, 70-150 Hz) across the MEA, as it has recently been shown to originate from both local neuronal firing and top-down predictive information [1]. We found a significant BHA-related peak at about 400 ms, coinciding with the lexical semantics peak from above. To ensure that this peak does reflect top-down predictive information and not underlying neuronal firing [2], we also fitted a word-onset mTRF to firing rate, and observed no significant peak at 400 ms (Supplementary Fig. 24b).

We also investigated whether there is a directional relationship between the low-dimensional phonetic and semantic trajectories by computing Granger temporal forecasting between their low-dimensional projections. We used the multivariate Granger causality toolbox (MVGC v1.3, 2022), which is based on a state-space formulation of Granger temporal forecasting analysis [3, 4]. We first applied a half-Gaussian filter 25 ms wide to the spiking traces of individual units to obtain a causal firing rate estimate. The resulting firing rates were then projected into the three-dimensional phonetic and semantic PC spaces, constructed by performing PCA on the corresponding

phonetic and semantic kernels as described above. The Granger models were created by separately predicting each of the three PCs of one feature (e.g. phonetic) from all three dimensions of another feature (e.g. semantic). We then combined the resulting Granger coefficients into two 3-by-3 matrices, one for phonetic-to-semantic and another one for semantic-to-phonetic predictions (Supplementary Figure 24c). Autoregression model parameters were estimated from data via the Levinson-Wiggins-Robinson algorithm. The order of AR models was selected via the Akaike Information Criterion (AIC). Statistical significance of estimated Granger causality measures was assessed via the corresponding F-test as implemented by the toolbox.

We found more significant effects in the top-down direction (Supplementary Fig. 24c). However, caution is needed when interpreting these results: Granger temporal forecasting is limited by several factors [5]: (i) it assumes linearity in the relationships between variables; (ii) it can be influenced by other confounding variables that are not accounted for in the model or unmeasured components of the neural system; (iii) it requires stationarity, meaning the statistical properties of the system must remain constant over time. These assumptions are necessary all met by our data.

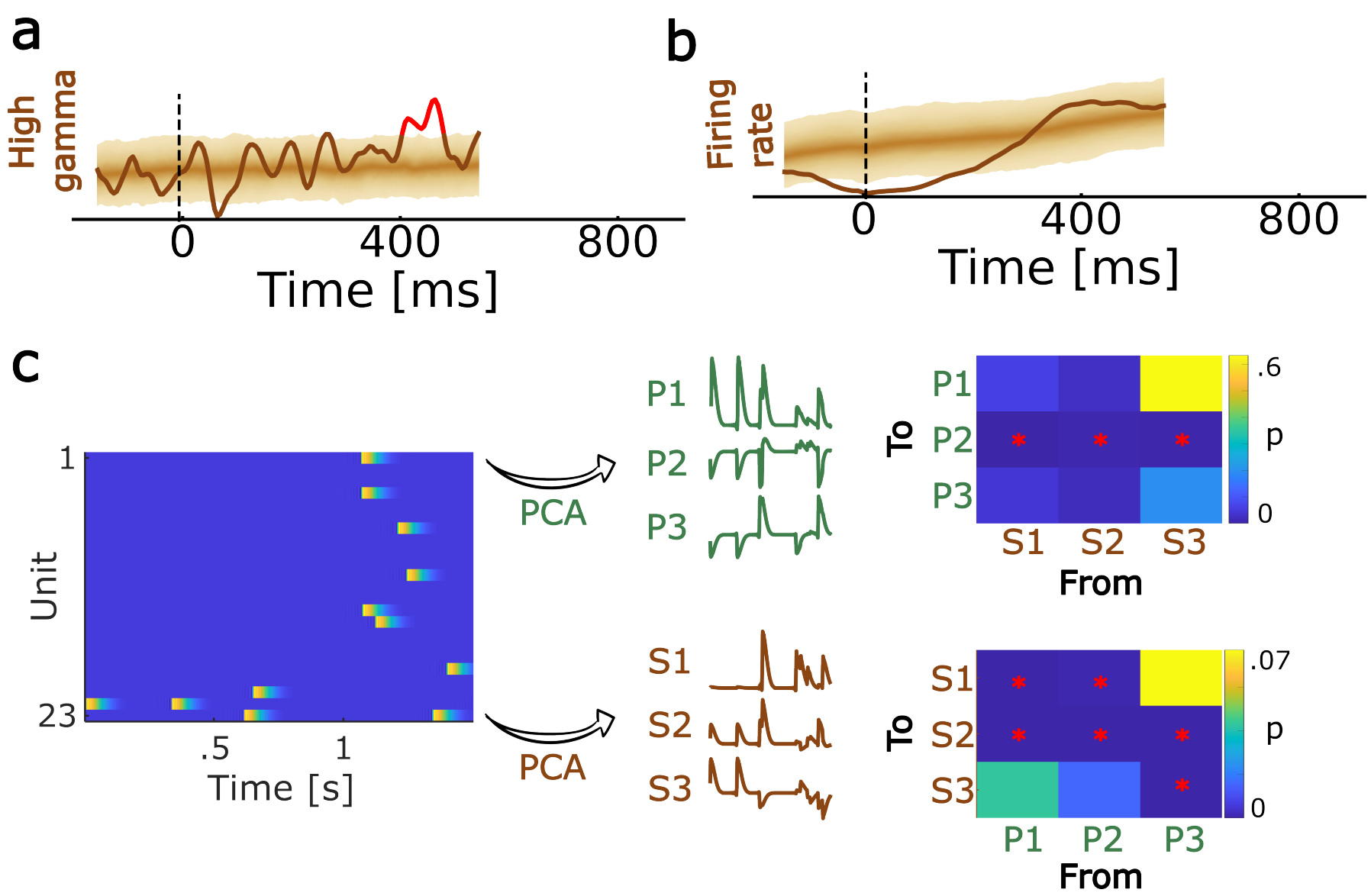

Supplementary Fig. 24 | Additional analyses for the top-down alignment in Participant 1. a. Kernel for the broadband high-frequency activity (BHA, 70-150 Hz) across the MEA showed a significant peak at around 400 ms, overlapping with the significant periods of the lexical semantic firing rate kernel (main Fig. 4b), and the beta-band word-onset kernel (main Fig. 4c). The 95% confidence region of the surrogate (chance-level) distribution is shown with brighter shading for increasingly peripheral percentiles. Red segments indicate periods surviving multiple comparison correction. **b.** Firing rate kernel aligned to word onset did not have a significant peak at 400 ms, suggesting that the observed BHA peak is not resulting from the underlying neuronal spiking. Coloring as in a. **c.** Granger temporal forecasting between phonetic and semantic low-dimensional representations. The left plot shows a 1.5-second snippet of the spiking data recorded during natural speech perception. Spiking activity was smoothed with a 25-ms half-Gaussian kernel to obtain a causal firing rate estimates. These are then projected onto three PCs to obtain time-varying phonetic (P) and semantic (S) features, which are then used in the Granger temporal forecasting analysis (MVGC toolbox). Matrices indicate p-values of Granger's F-test for a temporal forecasting relationship from the dimensions indicated on the x-axis to the dimensions indicated on the y-axis. The stars indicate significant relationships at the 0.05 threshold. While there were significant temporal forecasting relationships in both directions (i.e. phonetic to semantic and vice versa), there were more temporal forecasting relationships in the top-down direction.

Quality control of ECoG BHA responses

We performed an additional quality-control analysis to test whether the ECoG responses used in the encoding analysis contained residual artifact-contaminated word epochs. This analysis was performed on the same broadband high-frequency activity (BHA) responses used in the ECoG encoding model. The BHA responses were computed after preprocessing of the ECoG recordings, including line-noise removal and rereferencing. The analysis had three steps.

First, we identified abnormal word-by-contact pairs. For each word epoch and each ECoG contact, we computed four measures of possible artifact contamination: (i) the 99th percentile of the absolute BHA value within the epoch, which captures unusually large signal values; (ii) the peak-to-peak range, defined as the maximum value minus the minimum value within the epoch, which captures unusually large changes across the word-locked window; (iii) the standard deviation across time within the epoch, which captures unusually variable responses; and (iv) the largest absolute difference between neighboring time points, which captures abrupt jumps in the signal. Each measure was evaluated separately for each ECoG contact. For a given contact and measure, values across word epochs were converted to z-scores using the median and median absolute deviation (MAD):

$$z_{\text{robust}} = \frac{x - \text{median}(x)}{1.4826 \times \text{MAD}(x)}$$

where

$$\text{MAD}(x) = \text{median}\left(|x - \text{median}(x)|\right).$$

The factor 1.4826 is the standard consistency factor that rescales MAD to estimate the standard deviation under normally distributed data [6, 7]. This normalization was performed within each contact, so that each word epoch was evaluated relative to the typical values of the same contact, rather than by comparing raw signal magnitudes across different contacts. A word-by-contact pair was marked as artifactual if any of the four measures exceeded a z-score of 8, indicating clear outliers in individual contacts.

Second, we used these word-by-contact flags to decide whether any word epochs should be removed. For each word epoch, we counted how many ECoG contacts were marked as artifactual in that same epoch. We refer to this count as "badness per word". A word epoch was removed if at least 8 ECoG contacts were marked as artifactual in that epoch. This criterion identifies epochs in which the abnormality is present across several contacts at the same time, as expected for movement, jaw, or recording transients, rather than isolated fluctuations in a single contact.

Third, we used the same word-by-contact flags to check whether any ECoG contacts were persistently noisy. For each contact, we computed the fraction of word epochs in which that contact was marked as artifactual. We refer to this quantity as "badness per channel". A contact would have been removed if it had been marked as artifactual in more than 15% of word epochs. This is a separate channel-level check: the robust z-score threshold identifies abnormal contact-epoch pairs, the word-level criterion removes contaminated epochs, and the 15% criterion tests whether any contact is repeatedly contaminated across the recording.

The analysis identified two word epochs in which 23 and 24 ECoG contacts were marked as artifactual. In contrast, no ECoG contact was marked as artifactual in more than 15% of word epochs, so no channels were removed. GR35, the contact closest to the Utah array, showed a low artifact rate comparable to the other ECoG contacts. This indicates that the MEA-proximal ECoG contact did not show prominent persistent artifact contamination. After removing the contaminated epochs, the ECoG encoding analysis remained unchanged: MEA-proximal ECoG contacts still did not show significant encoding effects.

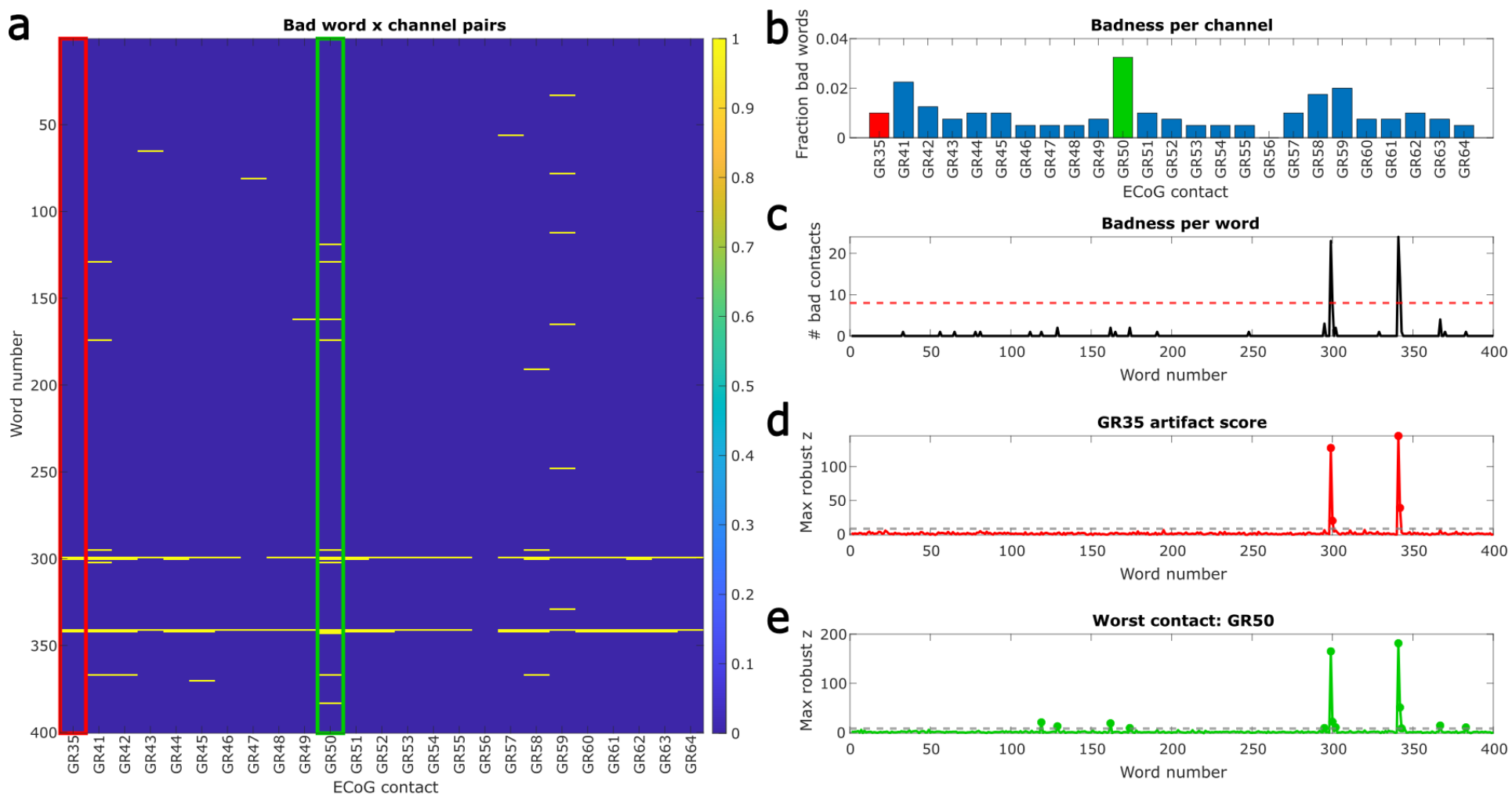

Supplementary Fig. 25 Quality control of ECoG BHA responses. a. Binary artifact matrix for word-locked BHA epochs. Rows indicate word epochs and columns indicate ECoG contacts. Yellow entries mark word-by-contact pairs in which at least one artifact measure exceeded the z-score threshold of 8. GR35, the contact closest to the Utah array, is outlined in red. The contact with the highest fraction of flagged epochs is outlined in green. **b**. Badness per channel, defined as the fraction of word epochs in which each ECoG contact was flagged. **c**. Badness per word, defined as the number of ECoG contacts flagged for each word epoch. The dashed red line marks the word-rejection threshold of 8 flagged contacts. Two word epochs exceeded this threshold, consistent with brief global artifacts affecting many contacts simultaneously. Overall, artifacts were concentrated in two word epochs affecting many contacts simultaneously, while no ECoG contact showed persistent artifact contamination across epochs **d**. Maximum robust artifact score across word epochs for GR35, the contact closest to the Utah array. Red points indicate epochs in which GR35 exceeded the z-score threshold of 8. **e**. Maximum robust artifact score across word epochs for GR50, the contact with the highest artifact rate. Green points indicate epochs in which GR50 exceeded the z-score threshold of 8.

References

[1] Leszczyński, M., Barczak, A., Kajikawa, Y., Ulbert, I., Falchier, A.Y., Tal, I., Haegens, S., Melloni, L., Knight, R.T., Schroeder, C.E.: Dissociation of broadband high-frequency activity and neuronal firing in the neocortex. Science Advances **6**(33), 0977 (2020) https://doi.org/10.1126/sciadv.abb0977 . Chap. Research Article

[2] Crone, N.E., Boatman, D., Gordon, B., Hao, L.: Induced electrocorticographic gamma activity during auditory perception. Clinical Neurophysiology **112**(4), 565–582 (2001) https://doi.org/10.1016/S1388-2457(00)00545-9

[3] Barnett, L., Seth, A.K.: The MVGC multivariate Granger causality toolbox: A new approach to Granger-causal inference. Journal of Neuroscience Methods **223**, 50–68 (2014) https://doi.org/10.1016/j.jneumeth.2013.10.018

[4] Barnett, L., Seth, A.K.: Granger causality for state-space models. Physical Review E **91**(4), 040101 (2015) https://doi.org/10.1103/PhysRevE.91.040101

[5] Shojaie, A., Fox, E.B.: Granger Causality: A Review and Recent Advances. Annual Review of Statistics and Its Application **9**(1), 289–319 (2022) https://doi.org/10.1146/annurev-statistics-040120-010930

[6] Rousseeuw, P.J., Croux, C.: Alternatives to the Median Absolute Deviation. Journal of the American Statistical Association **88**(424), 1273–1283 (1993) https://doi.org/10.2307/2291267 2291267

[7] Leys, C., Ley, C., Klein, O., Bernard, P., Licata, L.: Detecting outliers: Do not use standard deviation around the mean, use absolute deviation around the median. Journal of Experimental Social Psychology **49**(4), 764–766 (2013) https://doi.org/10.1016/j.jesp.2013.03.013
