# Supplementary material for: Top-down semantic predictions align phonetic neuronal dynamics in human superior temporal gyrus
Source: Nat Commun. 2026 Oct 3;17:11313. doi: 10.1038/s41467-026-77942-x (PMC13724739; doi:10.1038/s41467-026-77942-x)
Supplement: Supplementary file 2 [file 41467_2026_77942_MOESM2_ESM.pdf]

nature portfolio

Peer Review File

Top-down semantic predictions align phonetic neuronal dynamics in human superior temporal gyrus

Corresponding Author: Dr Pavo Orepic

This file contains all reviewer reports in order by version, followed by all author rebuttals in order by version.

Version 1:

Reviewer comments:

Reviewer #1

(Remarks to the Author)
This manuscript reports a relatively rare human intracortical MEA recordings from STG in three epilepsy patients, combined with ECoG, during an auditory semantic categorization task and conversational speech perception. The results will be of interest to the general readership of this journal. Though the data may be rare, the sample size is extremely small (n=3), and many other MEA human studies from 2024-26 report 10-25 patients. Still, the manuscript reports results that seem to be remarkably clear and convincing, and statistically robust (mTRF, permutation/cluster corrections etc). Indeed, the link from manifold-style population geometry with hierarchical speech processing is done using data at resolutions that are almost never available in humans. The central claim is that population activity in aSTG contains separable low-dimensional 'manifolds' for phonetic and semantic features, and that phonetic representations become temporally aligned to word-level semantic timing, consistent with top-down predictive mechanisms, with semantic information sculpting aspects of sensorimotor representations. I also think the attempt to show that phonetic geometry generalizes from a controlled task to natural speech in the same participant is a real strength.

I recommend the following revisions before it can be accepted in publishable form.

I wonder if the authors might want to explore some alternative explanations for their effects, like 'word' boundary or lexical access confounds (e.g., the process of searching for lexico-semantic and morphosyntactic features is inevitably triggered). Regressing phoneme order kernels is sensible, but in continuous speech many processes (acoustic, lexical access, prosodic boundary cues, motor entrainment) can be time-locked to word onsets and could pull kernels toward a common latency even without semantic prediction. This is basically a similar critique to many other sentence-level perception tasks that will always trigger a bunch of non-linguistic processes. One possible way to approach this would be to add explicit controls for word duration or phoneme count, and ideally lexical covariates like frequency, surprisal, phonotactic probability to show that the semantic-aligned peak is not a proxy for simply accessing the lexical item and its attendant morphosyntactic features.

The operationalization of semantics in natural speech is not very saliently tied to semantic prediction, which is not in itself a major problem, but it does force some re-framing. Maybe tone down or sharpen the claims, and either explicitly relabel the focus as word-level semantic access/lexico-semantic processing, or add a genuine predictive variable (any would do, like surprisal from a language model, cloze probability, etc) and test whether predictability modulates the alignment.

The discussion is appropriately cautious about the restricted sampling and possible functional micro-organization, and I applaud the authors for being mostly conservative here, but the claims about aSTG should be better integrated with semantic and speech models in the literature (see Hickok 2025, in particular). I am thinking here of the traditional psycholinguistic (and localizationist) implications, which could be discussed more. For instance, aSTG has been implicated in emotional and contextual aspects of semantics, usually more so than 'pure' lexico-semantic representations.

Relatedly, other intracranial work exposing semantic processing in aSTG and aSTS should be acknowledged and incorporated (Murphy et al. 2023; Pescatore et al. 2025; Thye et al. 2021).

The author's discovery of null ECoG effects in the face of their MEA effects (and their main discovery that many sub-symbolic properties of speech are represented in single units) should be related to contemporary neural models of language

that predict a clear dissociation between spiking effects for phonetic and semantics vs distinct processes at broadband gamma / LFP. For example, Murphy (2025) also invokes neural manifolds for properties observed by the authors, and is the only model I am aware of in the literature that would predict differences between single-unit phonetic and semantic signatures and ECOG gamma signatures. All other neural models of language, as far as I am aware, do not make predictions for units vs. high-frequency gamma. The notion of 'dynamical motifs' has also been used to explain local parsing of lexico-semantic features in language (Murphy 2025, Labancová & Kazanina 2025), and given that the authors' main claim in their manuscript is that they are "Extending the rapidly emerging neural manifold framework to speech processing", they should acknowledge these existing proposals.

Many analyses focus on the 23 most-spiking units (and, in the task, firing-rate thresholding). This may be necessary for SNR, and is understandable, but still it raises concerns about selection bias and stability of the manifold geometry. Perhaps in Suppl Material the authors might show the key results (phonetic clustering, semantic separation, alignment) across multiple inclusion thresholds (top X/Y/Z units; or all units with rate > X), or using a reliability-weighted approach. Having said this, if the authors have some ulterior logic or reasoning here that I may be missing, then I am happy for them to further clarify this.

The ATL is notoriously problematic for intracranial LFP recordings, due to jaw muscle artefacts, movement, and proximity to typical ictal effects (in addition to downstream effects from MTL). The authors should clarify how they dealt with this. Based on the methods, it doesn't look like they performed any trial-wise cleaning procedure, and merely applied some notch filters, CAR, then downsampling. So there was no active 'cleaning' procedure beyond this, which is problematic, since any trained neurologist will tell you that aSTG electrodes need to be treated differently from, say, inferior parietal electrodes. Different territory, different noise profiles – and ATL is the noisiest region of all. The implication of what I'm saying is that perhaps the reason they didn't recovery any effects in the LFP is because of noise. The authors should either strengthen their cleaning procedure or carefully clarify in the text why these ATL-specific issues are less of a threat to their data.

Please define semantic manifold/subspace separation more concretely (eg., orthogonality in PC space? Temporal segregation? Variance partition?).

Perhaps I am wrong here, but there is controversy in the field at the moment about why rotational dynamics alone cannot truly be considered a 'manifold' unless strongly linked to behavioral effects. I wonder if the authors might address some of these philosophical and methodological issues in their Discussion (i.e. how to dissociate circular, rotational dynamics, manifolds, etc).

Some basic lessons in philosophy of causation are needed. Granger causality does not elucidate genuine causal relationships. Since GC is used to support directionality, the authors should provide key model parameters and robustness (eg., downsampling, stationarity checks), and interpret cautiously (GC does *not* deliver mechanistic causation).

Minor:
Correct typos (performend, alignemnt)

References

Hickok, G. (2025). Wired for Words: The Neural Architecture of Language. Cambridge, MA: MIT Press.
Labancová, K., & Kazanina, N. (2025). Dynamical motifs for computations in language. Cognitive Neuroscience 16(1-4): 83-84.
Murphy, E. (2025). ROSE: A universal neural grammar. Cognitive Neuroscience 16(1-4): 49-80.
Murphy, E., Forseth, K.J., Donos, C., Snyder, K.M., Rollo, P.S., & Tandon, N. (2023). The spatiotemporal dynamics of semantic integration in the human brain. Nature Communications 14(1): 6336.
Pescatore, C.R.C. et al. (2025). Decoding semantics from natural speech using human intracranial EEG. bioRxiv: https://doi.org/10.1101/2025.02.10.637051.
Thye, M., Geller, J., , Szaflarski, J.P., Mirman, D. (2021). Intracranial EEG evidence of functional specialization for taxonomic and thematic relations. Cortex 140: 40-50.

(Remarks on code availability)

Reviewer #2

(Remarks to the Author)
This is an important study using extremely rare single unit recordings from microelectrode arrays (MEAs) in combination with ECoG recordings in the human brain to study the neural bases of speech comprehension. The study targeted phonetic and semantic encoding in an auditory semantic categorization task and in natural speech and investigated the relation between semantic and phonetic processing. The results indicate a distributed encoding of phonemes and semantic features at the local neuronal level (from the MEA recordings) but not in the neighboring ECoG electrodes' LFP signal. The dynamic phonetic organization observed in neural manifolds generalized to natural speech. In addition, phoneme encoding and semantic features were aligned to word onsets rather than phoneme encoding features unfolding along with the acoustic input. This result was interpreted as being in agreement with top-down semantic predictions aligning phonetic feature representations through predictive mechanisms. The study provides a window into how phonetic representations dynamically contribute to retrieving the meaning of speech during speech perception.
This is an important study with extremely valuable data and findings. I hope the comments below help solidify or moderate the claims it makes given some potential concerns.

1. Methods
• The choice of where to place the MEAs was made based on fMRI correlates of phonetic processing and semantic categorization obtained from a large fMRI database. There is therefore only an indirect link between where the MEAs were placed in the patients enrolled in this study and the fMRI results. This is an acceptable inference however if no other within patient result is available, such as electrical stimulation results or fMRI results from the patients enrolled as they are performing the same or similar tasks used in this study. Is this the case here? We know how language-related activations can be variable from one participant to the next as has been shown for example by Ev Fedorenko's work, so this could be an important consideration. Also, one of the 2 participants who participated in the semantic categorization task had their MEA placed in the right hemisphere, where the inference made for its placement are less strong than in the left hemisphere.

• It seems like the semantic decision and the perceptual category features were regressed separately in the mTRF analyses because the trials in which the participants responded incorrectly were not included in the semantic decision analysis but were in the perceptual analysis. Wouldn't the choice of including the incorrect trials in the perceptual analysis introduce some undue noise? If the participants decided that the word they heard represented an object that was smaller than a foot as bigger, maybe their perception of the object was different than assumed by the experimenters? It seems odd to compare results obtained from different trials here.

2. Results
• There were very few units that were responsive to the probed features in the semantic categorization task on average (8/289 total units recorded correlated with phoneme onsets, 1/289 to phonetic features, 1/289 for conceptual categories, and 2/289 for perceptual categories), and these were only found in participant 1. A reason why participant 2 may have shown no responsive units is because the MEA in this person was placed in the right hemisphere, but still the one patient with the MEA in the left hemisphere shows very few significant results. Could the authors comment on this and how it compares to other single unit studies?

• The neural manifold results seem more convincing, supporting the claim that neural population dynamics might be more suited for the understanding of language processing in the brain. However, the semantic neural manifold results are still weaker than the phonetic encoding ones, as none of the semantic results survived the correction for multiple representations. Could the authors comment on why that may be and how this could impact the claims made here?

3. Discussion
• The potential effect of where the MEAs were placed (left vs. right hemispheres) on the timing differences reported between participants are not discussed. This may be an important aspect to consider however given how lateralized language processing has been shown to be.

(Remarks on code availability)

Reviewer #3

(Remarks to the Author)
This manuscript presents an elegant and technically sophisticated investigation of phonetic–semantic interactions in human anterior superior temporal gyrus (aSTG) using intracortical recordings from microelectrode arrays. The dataset is rare and valuable: single-neuron resolution in human cortex during both an auditory semantic categorization task and natural speech perception represents a substantial empirical contribution in itself. The authors combine these recordings with low-dimensional population analyses to identify separable neural manifolds corresponding to phonetic and semantic features, and to test competing hypotheses about bottom-up versus top-down temporal alignment. The finding that phonetic activity aligns with word-level semantic representations in a manner consistent with top-down influence is compelling, and the contrast with adjacent ECoG—where these effects are not detected—nicely illustrates the importance of fine-grained population geometry at the spiking level. Several aspects of the work are particularly strong.

First, there is clear methodological rigor and innovation. The use of manifold analysis to characterize neural population structure is well-motivated and implemented. The identification of functionally distinct low-dimensional subspaces for phonetic and semantic representations provides a principled way to move beyond single-neuron selectivity and toward population-level coding geometry. I hope to see much more for this from this lab and the field!

The explicit comparison between a bottom-up cumulative account and a top-down predictive account of phonetic–semantic temporal alignment is a major strength, however, at the same time, I think the manuscript would benefit from more explicitly situating its findings within the extensive literature on top-down and word-level influences in speech perception. There is already substantial behavioral and neural evidence for word superiority effects, lexical biasing of phoneme perception (e.g., Ganong-type effects, speech rate effects), contextual modulation of early auditory responses, and rapid semantic influences on sublexical processing. In other words, the existence of top-down influences on phonetic processing is well established in psycholinguistics and neurolinguistics. The novelty of the present study is not that such influences occur, but that it reveals how they are instantiated at the level of neuronal population geometry. Making this distinction explicit would sharpen the studie's impact: the manuscript provides a population-dynamics account of a phenomenon that has previously been characterized at the behavioral, ERP, or non-invasive levels. Framing the advance in these terms would avoid the impression that the work primarily demonstrates the presence of top-down effects, and instead emphasize its unique insight

into their population dynamics implementation.

The manuscript advances an important claim: that semantic prediction reorganizes the population dynamics supporting phonetic representations. Framed in terms of low-dimensional dynamics and subspace structure, this is a meaningful step toward understanding how distant representational levels interface in cortex. I do wonder, though, if how this theoretical position accounts for how speech perception works before lexical inforamtion can contribute, or when top-down information is not particularly biasing, strong, or predictive. To be fair, I feel this way about predictive coding conclusions pretty much all the time.

To wit, the authors interpret the observed phonetic–semantic alignment as evidence for predictive coding. While the results are certainly consistent with top-down influence, I find the invocation of "predictive coding" somewhat underspecified. Predictive coding, on its best day, entails some specific computational and formal commitments… but those never seem to get specified. Leaving the reader wondering: 1) What exactly is being predicted (feature identity, category membership, probability distributions over lexical candidates, semantic features)? 2) In what representational format are these predictions encoded? 3)What constitutes the error signal, and how would it be expressed in the observed manifolds? 4)Through what circuit-level mechanism would semantic-level information reshape phonetic population geometry?

As currently presented, the term risks functioning as a broad explanatory label rather than a mechanistic account. I encourage the authors either to (i) more precisely articulate what aspects of predictive coding theory are directly supported by their findings, or (ii) frame their conclusions more conservatively as evidence for top-down semantic modulation of phonetic population dynamics, without overcommitting to a specific predictive coding framework. Clarifying this would substantially strengthen the theoretical contribution of the manuscript.

Secondly, the semantic categorization task likely induces strong top-down expectations. This is not a weakness—in fact, it is a useful manipulation—but it raises an important question about generality. I wonder to what extent might the observed reorganization of phonetic manifolds reflect task-driven predictive pressure rather than intrinsic, spontaneously engaged predictive transforms during everyday speech perception? Of course, this is not a real problem for the manuscript as it stands in its conclusoins now, as the inclusion of natural speech perception data is a major asset of the study and approach. However, the contrast between task and natural speech could be more explicitly leveraged to address this concern. For example: 1) Are the manifold geometries qualitatively similar across task and naturalistic contexts? I don't think this was there and I missed this…2) Is the strength of phonetic–semantic alignment modulated by task demands? 3)Does the top-down alignment persist, attenuate, or reorganize in the absence of explicit semantic categorization goals? Even a more explicit discussion of these issues would help situate the findings within a broader theory of speech processing beyond high-predictability laboratory contexts.

The identification of separable semantic and phonetic subspaces is exciting. However, the manuscript could further explore what this implies about representational organization: 1) Does functional separation in low-dimensional space imply partial orthogonality of coding axes? 2)How stable are these subspaces across time and conditions? 3)Does semantic prediction rotate, compress, or otherwise geometrically transform the phonetic manifold? The geometric interpretation is one of the most interesting aspects of the work. Expanding on the nature of these transformations—rather than focusing primarily on their predictive interpretation—could broaden the manuscript's impact and connect it more deeply to the emerging literature on population coding geometry.
The failure to detect the effect in adjacent ECoG recordings is compelling and important, and worrisome for people who can't get spike data! It reinforces the idea that fine-grained population structure at the spiking level contains information that may not survive spatial averaging. A slightly more explicit discussion of what this implies about measurement scale and representational granularity would further strengthen the translational relevance of the findings. I would love to hear more of what the authors think this means and its implications for future research.

Overall this is a beautifully executed study leveraging rare human intracortical data to address a foundational question about hierarchical speech and language processing. The population-level manifold approach is a major strength and provides a valuable demonstration of how neural geometry can reveal structure in encoding schemes that might otherwise remain hidden. By more clearly positioning the work relative to the extensive literature on top-down lexical and semantic influences —and by sharpening the theoretical claims regarding predictive coding—the manuscript will make a significant and conceptually precise contribution to the study of speech perception and neural population dynamics.

(Remarks on code availability)

Version 2:

Reviewer comments:

Reviewer #1

(Remarks to the Author)
The authors have comprehensively addressed all of my comments in great detail, and the new analyses and textual edits greatly improve the argumentation of the report and rigor of the results. I am confident that this new version is suitable for publication and believe it will make a strong impact.

(Remarks on code availability)

Reviewer #2

(Remarks to the Author)
I thank the authors for thoroughly addressing my previous comments. I have no further concerns.

(Remarks on code availability)

Reviewer #3

(Remarks to the Author)
I would like to congratulate the authors on an excellent, responsive revision. They have fully addressed my concerns and were ever thoughtful about it. I look forward to this work's impact on the field!

(Remarks on code availability)

Open Access This Peer Review File is licensed under a Creative Commons Attribution 4.0 International License, which permits use, sharing, adaptation, distribution and reproduction in any medium or format, as long as you give appropriate credit to the original author(s) and the source, provide a link to the Creative Commons license, and indicate if changes were made.
In cases where reviewers are anonymous, credit should be given to 'Anonymous Referee' and the source.
The images or other third party material in this Peer Review File are included in the article’s Creative Commons license, unless indicated otherwise in a credit line to the material. If material is not included in the article’s Creative Commons license and your intended use is not permitted by statutory regulation or exceeds the permitted use, you will need to obtain permission directly from the copyright holder.
To view a copy of this license, visit https://creativecommons.org/licenses/by/4.0/

Response to reviewers' comments

We thank the reviewers for the time and effort they have dedicated to reviewing our manuscript. We have now revised several sections of the manuscript to improve clarity, added new results, and correct minor issues as suggested by the reviewers. We hope these changes will successfully address the reviewers comments.

Answers to Reviewer 1

Comment R1.1 *This manuscript reports a relatively rare human intracortical MEA recordings from STG in three epilepsy patients, combined with ECoG, during an auditory semantic categorization task and conversational speech perception. The results will be of interest to the general readership of this journal. Though the data may be rare, the sample size is extremely small (n=3), and many other MEA human studies from 2024-26 report 10-25 patients. Still, the manuscript reports results that seem to be remarkably clear and convincing, and statistically robust (mTRF, permutation/cluster corrections etc). Indeed, the link from manifold-style population geometry with hierarchical speech processing is done using data at resolutions that are almost never available in humans. The central claim is that population activity in aSTG contains separable low-dimensional 'manifolds' for phonetic and semantic features, and that phonetic representations become temporally aligned to word-level semantic timing, consistent with top-down predictive mechanisms, with semantic information sculpting aspects of sensorimotor representations. I also think the attempt to show that phonetic geometry generalizes from a controlled task to natural speech in the same participant is a real strength. I recommend the following revisions before it can be accepted in publishable form.*

Answer: We appreciate the reviewer's positive feedback on our work and their constructive suggestions, which we address in detail below.

We agree that a few recent studies involving Utah array recordings in humans have reported larger sample sizes [9, 20, 52, 51, 5]. However, most of these studies focus on motor and premotor regions in human participants with significant impairment of the motor system [9, 20]. By contrast, the use of Utah array to study cognitive processing not directly related to motor output remain extremely rare. A notable exception is work of the group of Kareem Zaghloul, with Utah arrays located in the anterior temporal lobe, typically in visual categorization tasks [52, 51, 5]. By contrast, our work focuses on the superior temporal gyrus (STG), a region critical for auditory and speech processing, which has not been studied in the studies cited above.

Moreover, as noted by the reviewer, intracortical MEA recordings like those used in our study are becoming increasingly rare due to their invasive nature. At institutions like MGH, where our data were collected, such recordings are no longer performed since most clinical epilepsy monitoring has shifted from ECoG to SEEG. (ECoG requires a craniotomy, allowing MEA implantation, while SEEG does not). While newer technologies, such as Neuropixels, offer promising alternatives, they are typically implanted for only short durations, limiting their use in complex cognitive tasks and natural speech experiments

Given these constraints, we believe the uniqueness and quality of our dataset, combined with the robustness and consistency of our findings across participants and modalities in this study, provide significant contributions to the field. As such, we remain confident in the importance of this work despite the limitations in sample size.

We now acknowledge the small sample size in the discussion, and argue that the rare nature of these recordings and their focus on a critical brain region for auditory processing make our findings particularly valuable.

Comment R1.2 *I wonder if the authors might want to explore some alternative explanations for their effects, like 'word' boundary or lexical access confounds (e.g., the process of searching for lexico-semantic and morphosyntactic features is inevitably triggered). Regressing phoneme order kernels is sensible, but in continuous speech many processes (acoustic, lexical access, prosodic boundary cues, motor entrainment) can be time-locked to word onsets and could pull kernels toward a common latency even without semantic prediction. This is basically a similar critique to many other sentence-level perception tasks that will always trigger a bunch of non-linguistic processes. One possible way to approach this would be to add explicit controls for word duration or phoneme count, and ideally lexical covariates like frequency, surprisal, phonotactic probability to show that the semantic-aligned peak is not a proxy for simply accessing the lexical item and its attendant morphosyntactic features.*

Answer: We thank the reviewer for raising this important point. We agree that word-level variables related to lexical access, lexical predictability, and phonological word form could contribute to responses aligned to word onset. To address this, we performed an additional control analysis including four word-level covariates: word frequency, word duration, GPT-2 surprisal, and phonotactic probability.

We added these four covariates to the baseline model and refit the lexico-semantic and phoneme order mTRF kernels. We then repeated the alignment analysis shown in Fig. 4b (Fig. R1). The same phonetic–semantic alignment was preserved after adding these covariates, indicating that the semantic-aligned peak is not simply a proxy for lexical access, word predictability, word duration, or phonological word-form statistics.

As an additional check, we also fitted these four covariates using the same baseline model used for the other encoding analyses, containing word-onset and acoustic-edge features shown in Fig. 2c (Fig. R2). Among the four control regressors, we observed a significant encoding period only for the word duration kernel. Thus, although word duration was encoded in the same neuronal population, including this regressor did not alter the phonetic–semantic alignment. We added the following text and figures to the Supplementary Material, and referred to it in the main text:

Control for word-level lexical and phonological covariates

To test whether the phonetic–semantic alignment shown in Fig. 4b could be explained by word-level variables related to lexical access, lexical predictability, or phonological word form, we performed an additional control analysis including four covariates: word frequency, word duration, GPT-2 surprisal, and phonotactic probability.

Word frequency was computed for each transcribed word using the `wordfreq` Python package, which returns English Zipf-scale lexical frequency estimates. Higher values therefore correspond to more frequent words in English.

Word duration was computed directly from the manual word annotations as the difference between word offset and word onset.

GPT-2 surprisal was computed using the pretrained GPT-2 base model (124M parameters). For each word token in the natural-speech transcript, we constructed an input sequence consisting of the target word preceded by up to the previous ten words from the same sentence or audio segment.

The target word was always placed as the final word of the input sequence. Surprisal was computed as the negative summed log probability of the GPT-2 subword tokens corresponding to the target word, conditioned on the preceding context. For one-word utterances, a beginning-of-text token was prepended so that the probability of the word could still be estimated. This produced one surprisal value for each word token.

Phonotactic probability was computed from the CMU Pronouncing Dictionary. For each word, we used the first available dictionary pronunciation, removed lexical stress markers from phonemes, and modeled the pronunciation as a sequence of phoneme bigrams including start and end boundaries. Phoneme bigram probabilities were estimated from the full CMU dictionary with add-one smoothing. The phonotactic-probability value for each word was defined as the average log probability of its phoneme bigram sequence. Words absent from the dictionary were assigned the mean value of the regressor after z-scoring, corresponding to a neutral value in the mTRF model.

All four covariates were added as word-level regressors at the corresponding word onset times. Before inclusion in the mTRF model, each regressor was z-scored across word tokens. We first added these covariates to the baseline model and refit the lexical-semantic and phoneme-order kernels used for the phonetic–semantic alignment analysis. The alignment between the phoneme-order and lexical-semantic kernels was preserved, indicating that the alignment reported in Fig. 4b cannot be explained by word frequency, word duration, GPT-2 surprisal, or phonotactic probability.

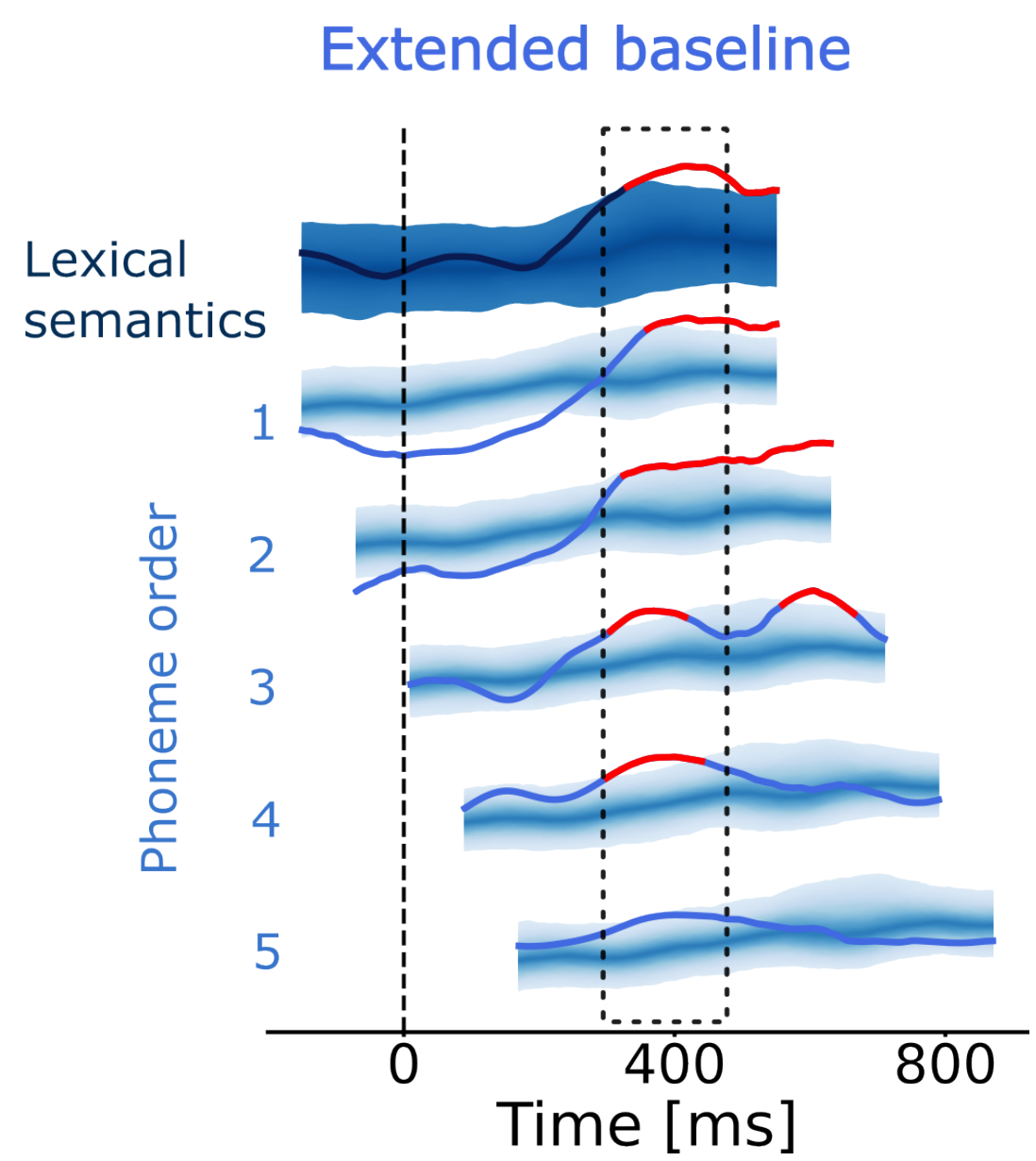

Figure R1: **Phonetic–semantic alignment after controlling for word-level lexical and phonological covariates.** The lexical-semantic and phoneme-order mTRF kernels were refit after adding word frequency, word duration, GPT-2 surprisal, and phonotactic probability to the baseline model. The alignment analysis was then repeated as in main Fig. 4b. The same phonetic–semantic alignment was preserved after including these additional word-level covariates, indicating that the semantic-aligned peak is not explained by lexical frequency, word duration, language-model surprisal, or phonotactic probability.

We also fitted the four control covariates directly using the same baseline model used for the

other encoding analyses, which included word-onset and acoustic-edge features (Fig. 2c). This analysis tested whether the four covariates were encoded in the neuronal population. Among the four regressors, we observed a significant encoding period only for word duration. Thus, word duration was represented in the same neuronal population, but accounting for it did not alter the phonetic–semantic alignment.

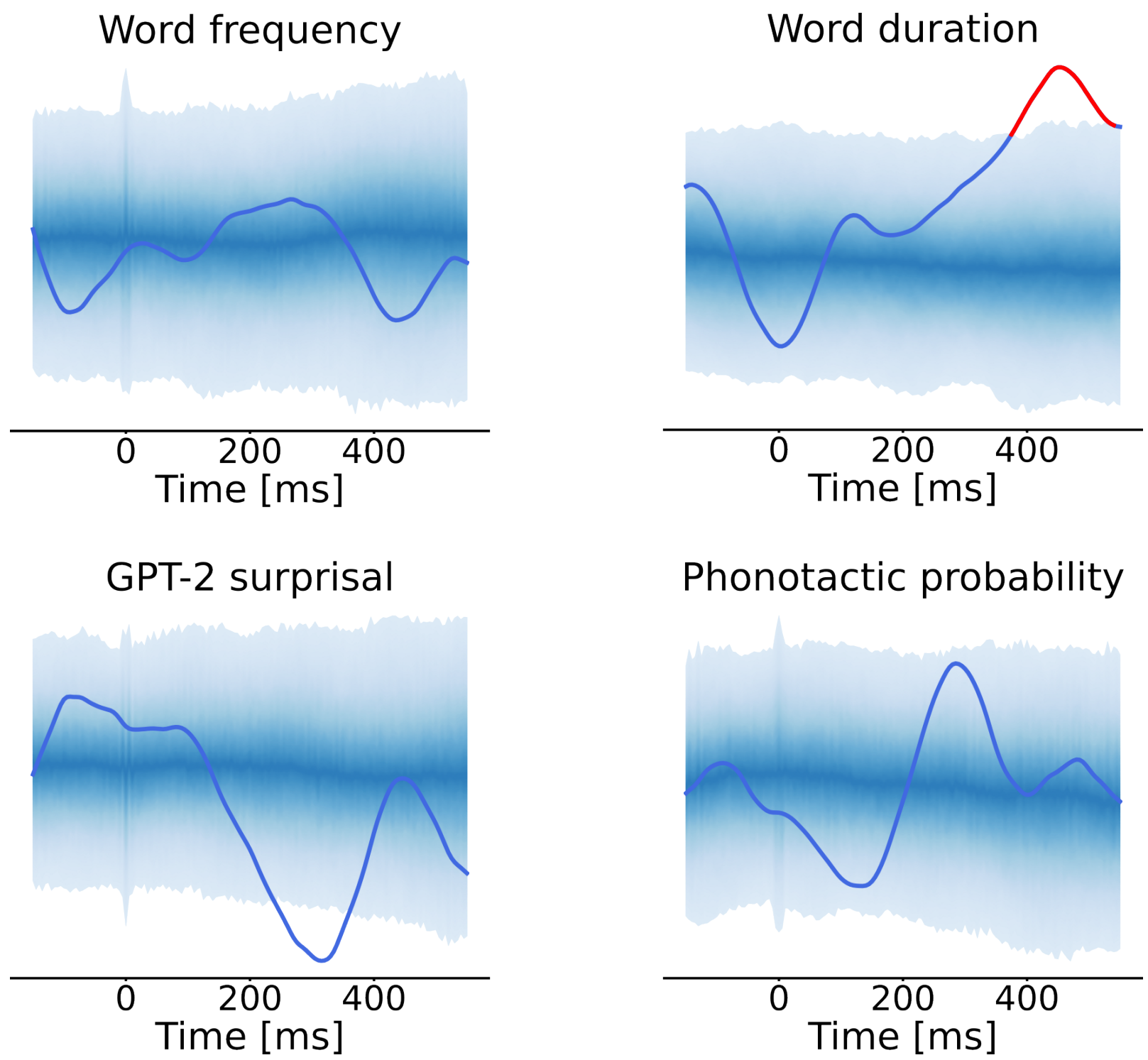

Figure R2: **Encoding of word-level lexical and phonological control covariates.** Kernels for word frequency, word duration, GPT-2 surprisal, and phonotactic probability were fitted using the same baseline model used for the other encoding analyses, containing word-onset and acoustic-edge features. Among these four covariates, a significant encoding period was observed only for word duration.

Together, these analyses show that the phonetic–semantic alignment is preserved after controlling for lexical frequency, word duration, GPT-2 surprisal, and phonotactic probability. The alignment therefore cannot be explained by general word-onset-locked lexical access, word predictability, word duration, or phonological word-form statistics.

Comment R1.3 *The operationalization of semantics in natural speech is not very saliently tied to semantic prediction, which is not in itself a major problem, but it does force some re-framing. Maybe tone down or sharpen the claims, and either explicitly relabel the focus as word-level semantic access/lexico-semantic processing, or add a genuine predictive variable (any would do, like surprisal from a language model, cloze probability, etc) and test whether predictability modulates the alignment.*

Answer: We appreciate the reviewer's feedback that clarification is needed regarding our operationalization of semantics in natural speech.

Our study primarily focuses on lexical-semantic processing rather than higher-level semantic prediction. Indeed, focusing on this level allows aligning our findings more accurately between the single-word experiment (first part of the study) and the natural speech experiment (second part of the study).

In light of this feedback, we have now revised all sections where semantics is discussed to reflect our focus on lexico-semantic processing, preventing confusion about the scope of our claims.

Comment R1.4 *The discussion is appropriately cautious about the restricted sampling and possible functional micro-organization, and I applaud the authors for being mostly conservative here, but the claims about aSTG should be better integrated with semantic and speech models in the literature (see Hickok 2025, in particular). I am thinking here of the traditional psycholinguistic (and localizationist) implications, which could be discussed more. For instance, aSTG has been implicated in emotional and contextual aspects of semantics, usually more so than 'pure' lexico-semantic representations.*

Answer: In agreement with the reviewer, Hickok recent book reviews studies suggesting that the aSTG is indeed involved in processes beyond pure lexico-semantic representations, notably including emotional and contextual aspects of semantics [12]. Specifically, the dorsal aspect of the anterior STG has been implicated in auditory-emotion processing, as demonstrated by imaging and non-invasive stimulation studies on social valence and music perception [18, 32, 23, 13].

However, our Utah arrays are not located on the dorsal aspect but rather at the surface of the gyrus, which is more lateral. The lateral part of the aSTG might not be as much involved in emotional processing, as such effect has not been reported in several studies focusing on this particular area [34, 53]. Rather, this specific area seems to better relate the aSTG role in bridging phonetic and semantic processes. Given its anatomical location, the aSTG indeed resides at the interface between these processes, as the mid-STG is associated with phoneme, syllable, and word encoding, while the anterior temporal lobe is involved in entity semantics and combinatorics [12, 32]. This integration is supported by fMRI studies [53] and our own systematic review of conceptual semantic and phonetic aspects through fMRI meta-analysis (phonetic: https://identifiers.org/neurovault.collection/22071, semantic: https://neurovault.org/collections/22048/). This aligns our findings with the dual-stream model of speech processing while expanding the understanding of aSTG's role in speech processing.

We have now added the above points to the Discussion section.

Comment R1.5 *Relatedly, other intracranial work exposing semantic processing in aSTG and aSTS should be acknowledged and incorporated (Murphy et al. 2023; Pescatore et al. 2025; Thye et al. 2021).*

Answer: We thank the reviewer for pointing out these important intracranial EEG studies that relate to semantic processing in the anterior superior temporal gyrus (aSTG) and anterior superior temporal sulcus (aSTS).

Murphy et al. [36] found that the aSTG is involved in narrow semantic references, specifically in

determining the probability of correctly identifying an object. This finding relates to our work on semantic predictions and supports our understanding of the aSTG's role in semantic processing.

Thye et al. (2021) [50] investigated semantic representations during a reading semantic relatedness judgment task, revealing that taxonomic semantic relationships (i.e. whether a concept falls into a category) were predominantly encoded in the anterior temporal lobe, including the aSTG and the temporal pole. Their findings are closely related to the judgment task, and their results align well with our semantic findings. The inclusion of contacts in the temporal pole and specific experimental constraints may explain why they observed stronger results at the LFP/ECoG level compared to our study.

Regarding Pescatore et al. [39], we have decided not to include this study for several reasons. First, unlike our study, their work focuses on natural speech production rather than perception. Speech production involves other sensorimotor and cognitive processes which could contribute additional effects to the experimental results. Furthermore, they employ decoding models, and as noted by Haufe et al. [11], interpreting the importance weights of decoders can be challenging. Finally, their manuscript is currently available only on bioRxiv and, to our knowledge, has not yet undergone complete peer review.

Thus, we have now acknowledged and discussed only Murphy et al. 2023 and Thye et al. 2021 in the manuscript.

Comment R1.6 *The author's discovery of null ECoG effects in the face of their MEA effects (and their main discovery that many sub-symbolic properties of speech are represented in single units) should be related to contemporary neural models of language that predict a clear dissociation between spiking effects for phonetic and semantics vs distinct processes at broadband gamma / LFP. For example, Murphy (2025) also invokes neural manifolds for properties observed by the authors, and is the only model I am aware of in the literature that would predict differences between single-unit phonetic and semantic signatures and ECOG gamma signatures. All other neural models of language, as far as I am aware, do not make predictions for units vs. high-frequency gamma. The notion of 'dynamical motifs' has also been used to explain local parsing of lexico-semantic features in language (Murphy 2025, Labancová & Kazanina 2025), and given that the authors' main claim in their manuscript is that they are "Extending the rapidly emerging neural manifold framework to speech processing", they should acknowledge these existing proposals.*

Answer: We thank the reviewer for bringing Murphy's paper (2025) [35] to our attention. The reviewer rightly points out that our discovery of null ECoG effects in the face of robust MEA effects, particularly concerning the representation of sub-symbolic properties of speech at the single-unit level, aligns with the predictions of the recent theoretical framework (ROSE) proposed by Murphy. Specifically, Murphy's model predicts a clear dissociation between spiking activity for phonetic and semantic representations and the aggregation of these representations as manipulable objects observed in broadband high-gamma activity.

In line with this work, we find that the fine-grained trajectories in the neural state space used for representation as captured by MEA recordings are not detectable at the coarser mean-field spatial resolution of ECoG. However, we emphasize that this does not imply an absence of effects at the ECoG level; indeed, we did obtain effects that were spatially distinct from the MEA location,

localized closer to primary auditory cortex or mid-STG regions. This regional specificity precludes a broad generalization about the functional relationship between single-unit and high-gamma signals, and we remain cautious about the interpretation of our findings. Rather, this relationship might depend on the functional specialization of the recorded brain area. Nevertheless, we now acknowledge this work in our discussion.

The reviewer also raises an important point about the notion of "dynamical motifs" and their role in parsing lexico-semantic features, as discussed by Murphy (2025) [35] and Labancová and Kazanina (2025) [22]. While we acknowledge the value of this concept, we would like to highlight that the core idea of dynamical motifs, defined in our study as trajectories on neural manifolds, has precedents in earlier work, where it is called structured flows on manifolds, and has been applied in the context of language processing as well [38, 40]. These studies provide a broader theoretical and dynamical context for understanding how neural populations represent and process linguistic information dynamically.

In response to these points, we have now cited the above studies into our manuscript. We have also refined our discussion to better align with contemporary neural models of language, emphasizing the contribution of our findings within this theoretical landscape.

Comment R1.7 *Many analyses focus on the 23 most-spiking units (and, in the task, firing-rate thresholding). This may be necessary for SNR, and is understandable, but still it raises concerns about selection bias and stability of the manifold geometry. Perhaps in Suppl Material the authors might show the key results (phonetic clustering, semantic separation, alignment) across multiple inclusion thresholds (top X/Y/Z units; or all units with rate > X), or using a reliability-weighted approach. Having said this, if the authors have some ulterior logic or reasoning here that I may be missing, then I am happy for them to further clarify this.*

Answer: We understand the reviewer's concerns that our results might be biased by our focus on the 23 most-spiking units. Our mTRF and neural manifolds analysis focus on firing rate representations rather than sparse temporal coding schemes. On one hand, sparse spiking units have little effect on the firing rate and do not strongly affect the trajectories in the neural manifold. On the other hand, identifying whether these units contribute through a temporal coding scheme would necessitate completely different methods and research questions than the ones we are asking in the manuscript.

Nevertheless, to test whether the main mTRF results depended on selecting the 23 highest-spiking units, we repeated the phonetic and semantic mTRF analyses for Participant 1 using all recorded units. The resulting phonetic and semantic kernels preserved the same temporal structure as those shown in Fig. 2a, indicating that the reported kernel shapes were not driven only by the unit-selection criterion (Fig. R3). We added this control analysis and figure to the Supplementary Material, to which we refer in the main text.

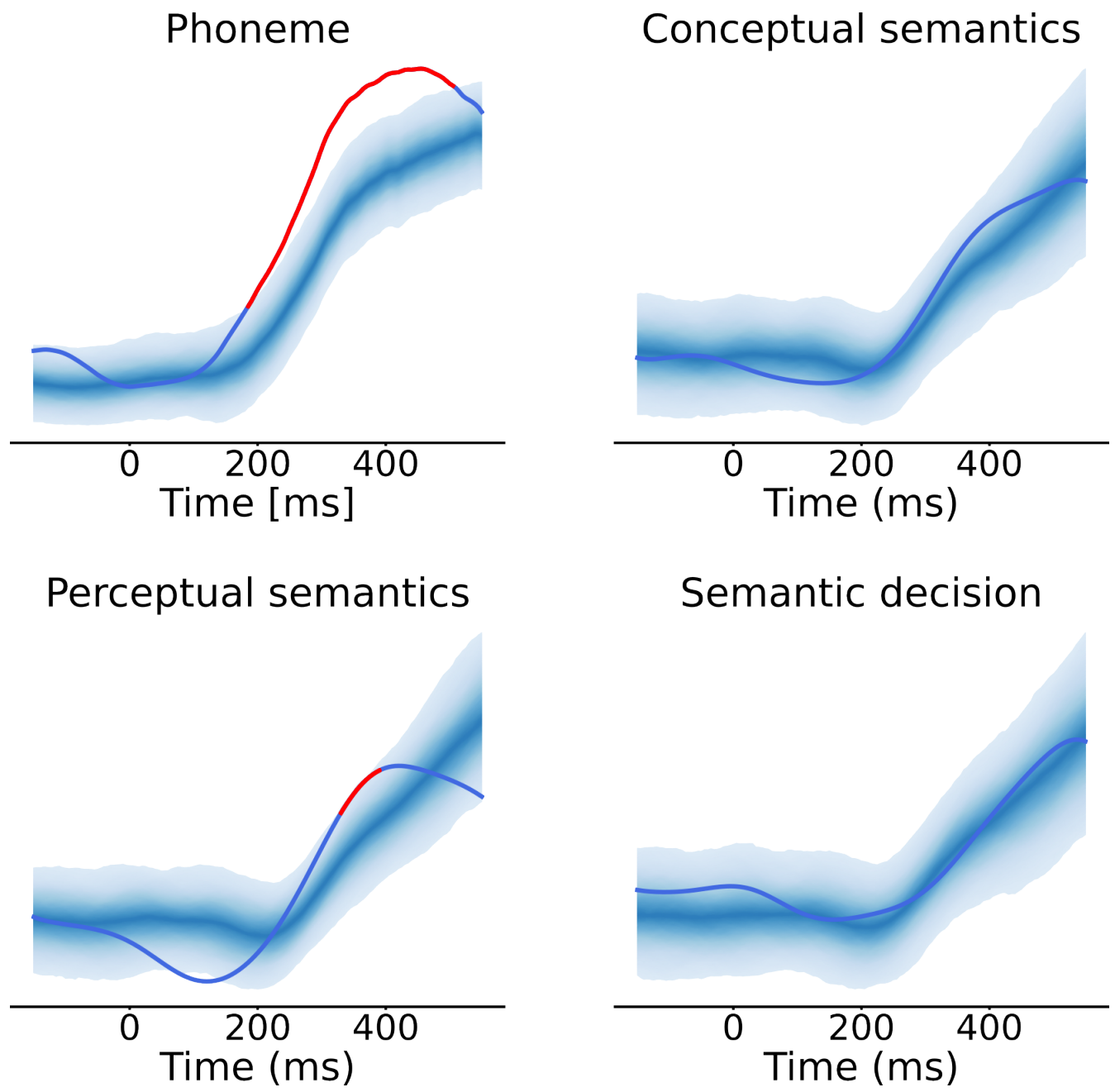

Figure R3: **Phonetic and semantic mTRF kernels using all units in Participant 1.** To test whether the mTRF results depended on selecting the 23 highest-spiking units, we repeated the main phonetic and semantic encoding analyses for Participant 1 using all recorded units. Specifically, we refit the same phonetic and semantic mTRF models used in Fig. 2a, but included the full set of units recorded from Participant 1 rather than restricting the analysis to the highest-spiking subset. The four panels show the phonetic and semantic mTRF kernels for the same model components shown in Fig. 2a. Lines indicate the mean kernel across units. The 95% confidence region of the surrogate (chance-level) distribution is shown with brighter shading for increasingly peripheral percentiles. The resulting kernels preserved the same overall temporal profiles as the kernels reported in the main text. This indicates that the main phonetic and semantic response patterns were not driven solely by the unit-selection criterion.

We also note that finding that only a few units significantly contribute to feature representation is common as one progresses to higher cognitive regions. For instance, data from the International Brain Laboratory [14] revealed sparse single-cell encoding, with only 0.5% units showing responsiveness across the brain, and not exceed about 10% in the most responsive brain regions. Other single-unit studies on speech processing also find very low numbers. For instance, Jang et al. [15], utilizing Utah arrays in the anterior part of the middle temporal gyrus during verbal memory tasks, found responsiveness ranging from 5% to 10%, which is more consistent with our findings.

Comment R1.8 *The ATL is notoriously problematic for intracranial LFP recordings, due to jaw muscle artefacts, movement, and proximity to typical ictal effects (in addition to downstream effects from MTL). The authors should clarify how they dealt with this. Based on the methods, it doesn't look like they performed any trial-wise cleaning procedure, and merely applied some notch filters, CAR, then downsampling. So there was no active 'cleaning' procedure beyond this, which is problematic, since any trained neurologist will tell you that aSTG electrodes need to be treated differently from, say, inferior parietal electrodes. Different territory, different noise profiles – and ATL is the noisiest region of all. The implication of what I'm saying is that perhaps the reason they*

didn't recovery any effects in the LFP is because of noise. The authors should either strengthen their cleaning procedure or carefully clarify in the text why these ATL-specific issues are less of a threat to their data.

Answer: We appreciate the reviewer's comment regarding the challenge of recording intracranial LFP in the anterior temporal lobe (ATL) and acknowledge the importance of addressing this issue. The ATL is indeed known to be particularly susceptible to artifacts such as jaw muscle activity, movement, and proximity to ictal activity in the medial temporal lobe, hippocampus and amygdala, which can complicate data interpretation [16, 19]. We note that the spatial distribution of these artifacts, as reported in the literature, show that they are more prevalent in the anterior half of the ventral and medial temporal lobe, which is somewhat remote from our primary region of interest in the aSTG.

Nevertheless, to test whether the ECoG/LFP results could be affected by these artifacts, we performed an additional quality-control analysis on our ECoG data by reproducing our findings after exclusion of all trials potentially affected by artifacts. For each word epoch and ECoG contact, we tested whether the BHA signal contained unusually large values or changes across the epoch, unusually high variability, or abrupt jumps between neighboring time points, relative to the other word epochs from the same contact. This analysis identified two word epochs that were flagged across many ECoG contacts, consistent with brief global artifacts affecting many contacts at the same time. In contrast, no ECoG contact showed an elevated artifact rate across epochs, indicating that there were no persistently noisy contacts. Importantly, GR35, the ECoG contact closest to the Utah array, showed a low artifact rate comparable to the other contacts. This suggests that there were no prominent persistent ATL/MEA-proximal artifacts of the type raised by the reviewer, apart from the two transient contaminated epochs. We therefore removed all epochs that met the artifact criterion and repeated the ECoG encoding analysis. The MEA-proximal ECoG contacts still did not show significant encoding effects. Thus, the absence of significant ECoG encoding near the MEA cannot be explained by persistent artifacts in ATL contacts or by the small number of transient contaminated epochs.

We added the following text summarizing this analysis and figure in the Supplementary Material, which we now refer to in the Methods:

Quality control of ECoG BHA responses

We performed an additional quality-control analysis to test whether the ECoG responses used in the encoding analysis contained residual artifact-contaminated word epochs. This analysis was performed on the same broadband high-frequency activity (BHA) responses used in the ECoG encoding model. The BHA responses were computed after preprocessing of the ECoG recordings, including line-noise removal and rereferencing. The analysis had three steps.

First, we identified abnormal word-by-contact pairs. For each word epoch and each ECoG contact, we computed four measures of possible artifact contamination: (i) the 99th percentile of the absolute BHA value within the epoch, which captures unusually large signal values; (ii) the peak-to-peak range, defined as the maximum value minus the minimum value within the epoch, which captures unusually large changes across the word-locked window; (iii) the standard deviation across time within the epoch, which captures unusually variable responses; and (iv) the largest absolute

difference between neighboring time points, which captures abrupt jumps in the signal. Each measure was evaluated separately for each ECoG contact. For a given contact and measure, values across word epochs were converted to z-scores using the median and median absolute deviation (MAD):

$$z_{\text{robust}} = \frac{x - \text{median}(x)}{1.4826 \times \text{MAD}(x)}$$

where

$$\text{MAD}(x) = \text{median}\left(|x - \text{median}(x)|\right).$$

The factor 1.4826 is the standard consistency factor that rescales MAD to estimate the standard deviation under normally distributed data [44, 27]. This normalization was performed within each contact, so that each word epoch was evaluated relative to the typical values of the same contact, rather than by comparing raw signal magnitudes across different contacts. A word-by-contact pair was marked as artifactual if any of the four measures exceeded a z-score of 8, indicating clear outliers in individual contacts.

Second, we used these word-by-contact flags to decide whether some word epochs should be removed. For each word epoch, we counted how many ECoG contacts were marked as artifactual in that same epoch. We refer to this count as "badness per word". A word epoch was removed if at least 8 ECoG contacts were marked as artifactual in that epoch. This criterion identifies epochs during which the anomaly abnormality is present across several contacts at the same time, as expected for movement, jaw, or recording transients, rather than isolated fluctuations in a single contact.

Third, we used the same word-by-contact flags to check whether any ECoG contacts were persistently noisy. For each contact, we computed the fraction of word epochs in which that contact was marked as artifactual. We refer to this quantity as "badness per channel". A contact would have been removed if it had been marked as artifactual in more than 15% of word epochs. This is a separate channel-level check: the robust z-score threshold identifies abnormal contact-epoch pairs, the word-level criterion removes contaminated epochs, and the 15% criterion tests whether any contact is repeatedly contaminated across the recording.

The analysis identified two word epochs during which 23 and 24 ECoG contacts were marked as artifactual. In contrast, no ECoG contact was marked as artifactual in more than 15% of word epochs, so no channels were removed. GR35, the contact closest to the Utah array, showed a low artifact rate comparable to the other ECoG contacts. This indicates that the MEA-proximal ECoG contact did not show prominent persistent artifact contamination. After removing the contaminated epochs, the ECoG encoding analysis remained unchanged: MEA-proximal ECoG contacts still did not show significant encoding effects.

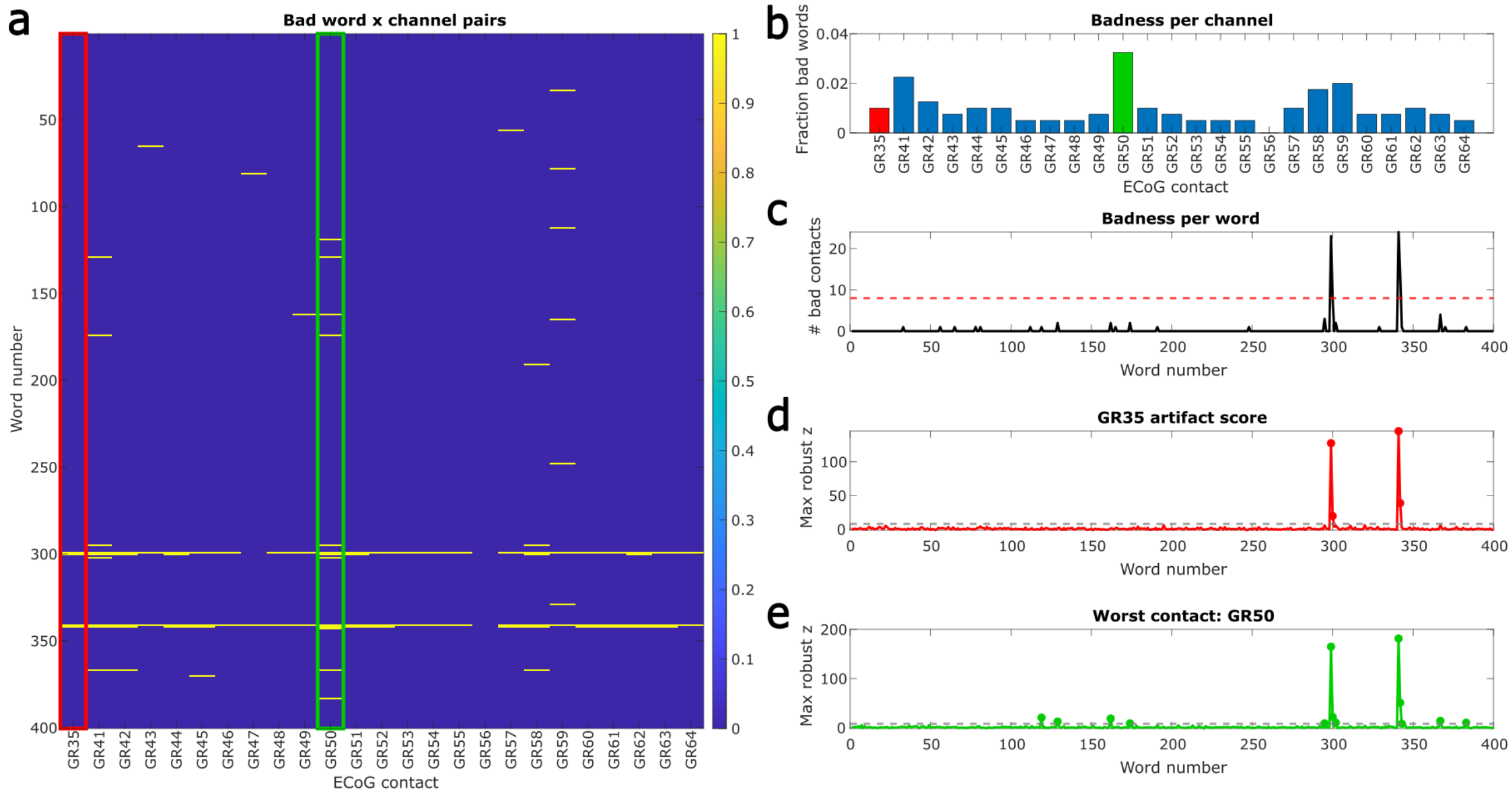

Figure R4: **Quality control of ECoG BHA responses. a.** Binary artifact matrix for word-locked BHA epochs. Rows indicate word epochs and columns indicate ECoG contacts. Yellow entries mark word-by-contact pairs in which at least one artifact measure exceeded the z-score threshold of 8. GR35, the contact closest to the Utah array, is outlined in red. The contact with the highest fraction of flagged epochs is outlined in green. **b.** Badness per channel, defined as the fraction of word epochs in which each ECoG contact was flagged. **c.** Badness per word, defined as the number of ECoG contacts flagged for each word epoch. The dashed red line marks the word-rejection threshold of 8 flagged contacts. Two word epochs exceeded this threshold, consistent with brief global artifacts affecting many contacts simultaneously. Overall, artifacts were concentrated in two word epochs affecting many contacts simultaneously, while no ECoG contact showed persistent artifact contamination across epochs **d.** Maximum robust artifact score across word epochs for GR35, the contact closest to the Utah array. Red points indicate epochs in which GR35 exceeded the z-score threshold of 8. **e.** Maximum robust artifact score across word epochs for GR50, the contact with the highest artifact rate. Green points indicate epochs in which GR50 exceeded the z-score threshold of 8.

Comment R1.9 *Please define semantic manifold/subspace separation more concretely (eg., orthogonality in PC space? Temporal segregation? Variance partition?).*

Answer: We assume the reviewer is referring to the separation of the trajectories within the different semantic subspaces. For both the task-related and natural-speech semantic clustering, we examined the separation between semantic kernels by calculating the Euclidean distance between their projection to the PCA space. For the task, the semantic kernels were defined by the design (e.g. animal or object), and for natural speech, the lexical semantics feature was defined by regressing each of the 11 Lancaster sensorimotor norms [30] at the corresponding word. We then computed the clustering index based on the separation between these multivariate semantic representations in PCA space. We hope that this explanation clarifies our methodology regarding the

semantic separation in our study. We have also clarified in the Methods section how the clustering index is computed between semantic kernels in the case of natural speech.

Additionally, we have added a new analysis directly comparing the geometries of the phonetic and semantic manifolds (see response to comment R3.6).

Comment R1.10 *Perhaps I am wrong here, but there is controversy in the field at the moment about why rotational dynamics alone cannot truly be considered a 'manifold' unless strongly linked to behavioral effects. I wonder if the authors might address some of these philosophical and methodological issues in their Discussion (i.e. how to dissociate circular, rotational dynamics, manifolds, etc).*

Answer: The reviewer raises an important point regarding the controversy surrounding the interpretation that rotational dynamics can be interpreted as a signature of a generative dynamical system as we and others have examined before [25, 41, 45]. We would first like to emphasize that rotational dynamics, as we understand it, refer to the trajectories on the manifold, rather than the manifold itself [41]. We do recognize that spurious rotational patterns can emerge from these methods, especially when applied to high-dimensional neural data. However, our study does not interpret the observed rotational dynamics as indicative of a recurrent dynamical system. Since this issue is only remotely connected to the analyses performed in our study, we decided to not add it to the discussion, in order to remain concise and to the point.

Comment R1.11 *Some basic lessons in philosophy of causation are needed. Granger causality does not elucidate genuine causal relationships. Since GC is used to support directionality, the authors should provide key model parameters and robustness (eg., downsampling, stationarity checks), and interpret cautiously (GC does *not* deliver mechanistic causation).*

Answer: We agree that Granger causality provides a "weak" assessment of causal relationships, as is based on purely statistical estimation rather than on inference based on actual interventions to probe causation [47, 1]. This means that while Granger causality can indicate (time-causal) directional dependencies between variables, it does not provide mechanistic evidence of causation. To avoid overstatement, we have now reformulated our statements. Instead, we now refer to "temporal forecasting," following the approach suggested by Siddiqi et al. [47]. This change in terminology reflects a more cautious interpretation of our results, more consistent with the limitations inherent to Granger causality.

Further, we recognize that Granger causality is limited by several factors [46]: (i) it assumes linearity in the relationships between variables; (ii) it can be influenced by other confounding variables that are not accounted for in the model or unmeasured components of the neural system; (iii) it requires stationarity, meaning the statistical properties of the system must remain constant over time. Since these assumptions are difficult to check extensively, we have decided to not report Granger causality results anymore in the main text. We left them in the Supplementary Materials, but added a discussion about the caution required in interpretation.

Comment R1.12 *Minor: Correct typos (performend, alignemnt)*

Answer: Thank you, we have fixed these typos.

Comment R1.13 *References*

Hickok, G. (2025). Wired for Words: The Neural Architecture of Language. Cambridge, MA: MIT Press.

Labancová, K., & Kazanina, N. (2025). Dynamical motifs for computations in language. Cognitive Neuroscience 16(1-4): 83-84.

Murphy, E. (2025). ROSE: A universal neural grammar. Cognitive Neuroscience 16(1-4): 49-80.

Murphy, E., Forseth, K.J., Donos, C., Snyder, K.M., Rollo, P.S., & Tandon, N. (2023). The spatiotemporal dynamics of semantic integration in the human brain. Nature Communications 14(1): 6336.

Pescatore, C.R.C. et al. (2025). Decoding semantics from natural speech using human intracranial EEG. bioRxiv: https://doi.org/10.1101/2025.02.10.637051.

Thye, M., Geller, J., , Szaflarski, J.P., Mirman, D. (2021). Intracranial EEG evidence of functional specialization for taxonomic and thematic relations. Cortex 140: 40-50.

Answers to Reviewer 2

Comment R2.1 *This is an important study using extremely rare single unit recordings from microelectrode arrays (MEAs) in combination with ECoG recordings in the human brain to study the neural bases of speech comprehension. The study targeted phonetic and semantic encoding in an auditory semantic categorization task and in natural speech and investigated the relation between semantic and phonetic processing. The results indicate a distributed encoding of phonemes and semantic features at the local neuronal level (from the MEA recordings) but not in the neighboring ECoG electrodes' LFP signal. The dynamic phonetic organization observed in neural manifolds generalized to natural speech. In addition, phoneme encoding and semantic features were aligned to word onsets rather than phoneme encoding features unfolding along with the acoustic input. This result was interpreted as being in agreement with top-down semantic predictions aligning phonetic feature representations through predictive mechanisms. The study provides a window into how phonetic representations dynamically contribute to retrieving the meaning of speech during speech perception. This is an important study with extremely valuable data and findings. I hope the comments below help solidify or moderate the claims it makes given some potential concerns.*

Answer: We thank the reviewer for the appreciation of our work and the suggested revisions, which we address in detail below.

Comment R2.2 *1. Methods*
The choice of where to place the MEAs was made based on fMRI correlates of phonetic processing and semantic categorization obtained from a large fMRI database. There is therefore only an indirect link between where the MEAs were placed in the patients enrolled in this study and the fMRI results. This is an acceptable inference however if no other within patient result is available, such as electrical stimulation results or fMRI results from the patients enrolled as they are performing the same or similar tasks used in this study. Is this the case here? We know how language-related activations can be variable from one participant to the next as has been shown for example by Ev Fedorenko's work, so this could be an important consideration. Also, one of the 2 participants who participated in the semantic categorization task had their MEA placed in the right hemisphere, where the inference made for its placement are less strong than in the left hemisphere.

Answer: We agree with the reviewer that there is a large variability across human participant language mapping studies, and that it is critical to consider this with respect to the placement of the Utah arrays in our study. We would like to clarify that the placement of these microeletrode arrays was determined solely based on clinical considerations, rather than prior direct fMRI results obtained from the patients before implantation. MEAs were implanted in the anterior superior temporal gyrus (aSTG) only because that area was also part of the region anticipated for resection during surgery, and not for any research purposes. This is already stated in the Methods.

We also emphasize that, in this study, we selected participants who had already been implanted with Utah arrays in the aSTG. A large fMRI database provided evidence for this region's involvement in phonetic processing and semantic categorization, which is why we chose those patients for this study. This approach allowed us to maximize the relevance of our findings while adhering to clinical constraints. Based on the fMRI meta-analysis, we selected three human participants

with Utah array implants in the aSTG. Out of these 3 participants, two performed the semantic categorization task, and two had natural speech segments.

Regarding the placement of one of the MEAs in the right hemisphere, we note that our fMRI meta-analysis revealed overlapping regions associated with phonetic and semantic processing on both sides of the brain. This bilateral representation is consistent with previous studies showing that semantic processing in the anterior temporal lobe (ATL) can be bilaterally distributed [42]. Moreover, all three participants demonstrated left-hemisphere language dominance through WADA testing, ensuring that our findings remain relevant to typical language organization.

We have now clarified our statement in the Results section.

Comment R2.3 *It seems like the semantic decision and the perceptual category features were regressed separately in the mTRF analyses because the trials in which the participants responded incorrectly were not included in the semantic decision analysis but were in the perceptual analysis. Wouldn't the choice of including the incorrect trials in the perceptual analysis introduce some undue noise? If the participants decided that the word they heard represented an object that was smaller than a foot as bigger, maybe their perception of the object was different than assumed by the experimenters? It seems odd to compare results obtained from different trials here.*

Answer: The reviewer is right that these two variables diverged on trials where the participant's response did not match the experimenter-defined category. The stimulus set was constructed so that half of the words were objectively bigger than a foot and the other half were smaller, yielding a balanced design. When participants responded differently, these trials were labeled as incorrect. However, because such trials made up about 18 % of the data, we do not think they simply reflect random noise, such as accidental button presses. Rather, they may capture a distinction between two processes: semantic information tied to the stimulus itself, and processes related to the participant's internal evaluation and decision.

The existence of these two different processes is the reason we decided to use two distinct regressors (instead of just one) relative to perceptual category and semantic decision separately, leading to two distinct regression models. Importantly, both analyses included the same trials; only the binary labels of the regressors differed for the approximately 18 % of trials on which response and stimulus-defined category diverged. We agree that this rationale was not sufficiently clear in the manuscript. We have now clarified this point.

Comment R2.4 *2. Results*
There were very few units that were responsive to the probed features in the semantic categorization task on average (8/289 total units recorded correlated with phoneme onsets, 1/289 to phonetic features, 1/289 for conceptual categories, and 2/289 for perceptual categories), and these were only found in participant 1. A reason why participant 2 may have shown no responsive units is because the MEA in this person was placed in the right hemisphere, but still the one patient with the MEA in the left hemisphere shows very few significant results. Could the authors comment on this and how it compares to other single unit studies?

Answer: We agree with the reviewer's observation regarding the limited number of units respon-

sive to probed features in the semantic categorization task across participants. After correction for multiple comparisons, we indeed found a total of 12/289 responding units, i.e. around 4% significant units.

First, we would like to highlight that our key takeaway is that, although responses of individual aSTG neurons may be sparse, population-level analyses can still reveal meaningful encoding patterns. This is because single units that do not significantly respond to a speech feature on their own may still covary with other responsive units and contribute to the population response. Our PC-based analysis captures this shared variance by projecting activity into the dimensions along which units covary most strongly. Thus, the apparent sparsity of responsive single neurons does not preclude robust encoding at the population level, and underscores the importance of considering population-level perspectives in understanding neural representations.

Nevertheless, we compared our findings with other recent single-unit studies:

1. Leonard et al. (2023)[26] reported that out of 685 putative units, a total of 287 exhibited significant correlations across various speech features, including acoustic, phonetic, prosodic, and sequence-level attributes (i.e., they here account for the sum of all significant units across features). However, their neuropixel probes were situated closer to the auditory cortex and parts of the STG directly involved in phonetic encoding, which may explain the higher number of responsive units.

2. Lakretz et al. (2021) [24] found 14/45 (31%) responsive units across both hemispheres, with electrodes distributed centrally in the STG.

3. Khanna et al. (2024)[17] reported around 10% of units responding to speech stimuli, with electrodes located in the posterior middle frontal gyrus.

4. Jang et al. (2017) [15], using Utah arrays in the anterior part of the middle temporal gyrus during verbal memory tasks, found responsiveness ranging from 5% to 10%, which is more consistent with our findings.

These comparisons highlight that as one progresses to higher cognitive regions, the sparsity of responsive neurons associated with relevant information increases, as also shown in our results. In line with this statement, animal data during other types of tasks from the International Brain Laboratory [14] also revealed sparse encoding in most brain regions, with only 0.5% units showing responsiveness across the brain, and not exceed about 10% in the most responsive brain regions.

Notably, in our study, these responsive units were only identified in Participant 1. The absence of significant results in Participant 2 might be attributed to the placement of the MEA on the right hemisphere, which is less ideal for capturing speech-related features compared to the left hemisphere.

We have now extended the Discussion section to address the above comparisons with other studies.

Comment R2.5 *The neural manifold results seem more convincing, supporting the claim that neural population dynamics might be more suited for the understanding of language processing*

in the brain. However, the semantic neural manifold results are still weaker than the phonetic encoding ones, as none of the semantic results survived the correction for multiple representations. Could the authors comment on why that may be and how this could impact the claims made here?

Answer: We thank the reviewer for raising this point. While the semantic results did not survive multiple comparisons correction in the task-based analysis, they were significant in the natural speech condition, where semantic processes are likely more widespread and prominent. This discrepancy suggests that the hypothesized top-down predictive mechanisms in the anterior superior temporal gyrus (aSTG) may be more strongly active during natural speech processing.

In natural speech, the ability to predict semantic content is influenced by prior context and the initial phonemes of words, which can enhance the accuracy of semantic predictions [21]. This context-dependent nature of semantic processing aligns with our hypothesis that the aSTG plays a key role in integrating top-down expectations with bottom-up sensory information. The stronger semantic results in natural speech further support this interpretation, as they likely capture the dynamic interplay between phonetic and semantic levels more faithfully than controlled tasks.

The weaker semantic effects in the task condition may also reflect the structure of the task itself. In our task, semantic judgments were constrained to specific binary distinctions, such as whether an item was bigger or smaller than a foot, rather than engaging the richer, continuous semantic context available during natural speech. Thus, the task may have elicited more limited or more variable semantic representations than natural speech comprehension.

Another reason could be that semantic representations are likely more distributed, temporally broader, and more variable across participants than phonetic ones. This is especially relevant in our study because the recordings come from a very small 4×4 mm sampling cortical patch in aSTG, which we note may lead to participant-specific effects depending on the exact implantation site.

Finally, the semantic analysis was intrinsically lower-powered: each semantic feature had only two regressors (e.g. object or animal), so the clustering measure was effectively reduced to the distance between two kernels, whereas the phonetic analyses relied on richer multivariate structure across many phonemes and phonetic classes.

In summary, the weaker semantic results in the task-based analysis and the significant findings in natural speech underscore the importance of considering both controlled tasks and ecologically valid conditions when studying language processing in the brain.

We have now added these points in the Discussion section.

Comment R2.6 *3. Discussion*
The potential effect of where the MEAs were placed (left vs. right hemispheres) on the timing differences reported between participants are not discussed. This may be an important aspect to consider however given how lateralized language processing has been shown to be.

Answer: The reviewer raises a crucial point regarding the potential impact of MEA placement in different hemispheres on the timing differences observed between participants. Language pro-

cessing is indeed predominantly lateralized, with the left hemisphere typically being dominant for phonetic processing. However, semantic processing has been shown to be bilaterally distributed in the anterior temporal lobe, as supported by Ralph et al. (2017) [42]. Our fMRI analysis further indicates that certain aspects of phonetic processing may also involve the right hemisphere, particularly when related to voice identity [29].

This bilateral involvement could explain the variability in our findings. Participant 1, with the MEA placed on the left hemisphere, showed significant results, aligning with the dominance of this hemisphere for phonetics. Conversely, Participant 2's MEA placement on the right hemisphere resulted in fewer significant findings, consistent with the understanding that speech processing functions are more localized to the left.

We have now addressed these lateralization effects in the Discussion.

Answers to Reviewer 3

Comment R3.1 *This manuscript presents an elegant and technically sophisticated investigation of phonetic–semantic interactions in human anterior superior temporal gyrus (aSTG) using intracortical recordings from microelectrode arrays. The dataset is rare and valuable: single-neuron resolution in human cortex during both an auditory semantic categorization task and natural speech perception represents a substantial empirical contribution in itself. The authors combine these recordings with low-dimensional population analyses to identify separable neural manifolds corresponding to phonetic and semantic features, and to test competing hypotheses about bottom-up versus top-down temporal alignment. The finding that phonetic activity aligns with word-level semantic representations in a manner consistent with top-down influence is compelling, and the contrast with adjacent ECoG—where these effects are not detected—nicely illustrates the importance of fine-grained population geometry at the spiking level. Several aspects of the work are particularly strong.*
First, there is clear methodological rigor and innovation. The use of manifold analysis to characterize neural population structure is well-motivated and implemented. The identification of functionally distinct low-dimensional subspaces for phonetic and semantic representations provides a principled way to move beyond single-neuron selectivity and toward population-level coding geometry. I hope to see much more for this from this lab and the field!

Answer: We thank the reviewer for the appreciation of our work.

Comment R3.2 *The explicit comparison between a bottom-up cumulative account and a top-down predictive account of phonetic–semantic temporal alignment is a major strength, however, at the same time, I think the manuscript would benefit from more explicitly situating its findings within the extensive literature on top-down and word-level influences in speech perception. There is already substantial behavioral and neural evidence for word superiority effects, lexical biasing of phoneme perception (e.g., Ganong-type effects, speech rate effects), contextual modulation of early auditory responses, and rapid semantic influences on sublexical processing. In other words, the existence of top-down influences on phonetic processing is well established in psycholinguistics and neurolinguistics. The novelty of the present study is not that such influences occur, but that it reveals how they are instantiated at the level of neuronal population geometry. Making this distinction explicit would sharpen the studie's impact: the manuscript provides a population-dynamics account of a phenomenon that has previously been characterized at the behavioral, ERP, or non-invasive levels. Framing the advance in these terms would avoid the impression that the work primarily demonstrates the presence of top-down effects, and instead emphasize its unique insight into their population dynamics implementation.*

Answer: The reviewer is correct that top-down semantic influences on phonetic processing are already well-established in psycholinguistics and neurolinguistics. In addition to the reviewer's comment, we would like to point out that this is what we meant to acknowledge in our manuscript by our reference to the analysis-by-synthesis theoretical framework [31, 10, 2, 49]. However, we indeed did not provide a detailed account of previous experimental findings about top-down effects. As noted by the reviewer, these effects manifest through phenomena such as word superiority effects [43, 6, 28], where words are recognized more easily than isolated letters, and Ganong-type effects

[7, 37, 3], where lexical context influences phonetic categorization. Additionally, speech rate effects [33] and contextual modulation of early auditory responses [4, 48] further illustrate the pervasive nature of these top-down processes.

As correctly pointed out by the reviewer, our study does not claim to demonstrate the existence of such effects, but rather provides a neural mechanistic account by examining the neuronal population-dynamics basis of these effects. We think this is an important advancement, as previous work has primarily characterized these effects at behavioral, ERP, or non-invasive levels without delving into the underlying neural population dynamics.

We have now extended the Introduction and Discussion sections to explicitly reference this extensive literature on top-down effects, ensuring that our study is appropriately situated within the broader context of psycholinguistics and neurolinguistics.

Comment R3.3 *The manuscript advances an important claim: that semantic prediction reorganizes the population dynamics supporting phonetic representations. Framed in terms of low-dimensional dynamics and subspace structure, this is a meaningful step toward understanding how distant representational levels interface in cortex. I do wonder, though, if how this theoretical position accounts for how speech perception works before lexical inforamtion can contribute, or when top-down information is not particularly biasing, strong, or predictive. To be fair, I feel this way about predictive coding conclusions pretty much all the time.*

Answer:

We appreciate the reviewer's thoughtful observation regarding how our theoretical framework accounts for situations where top-down information is weak or non-predictive.

In the manuscript we propose a bottom-up and a top-down hypothesis. Depending on the brain region studied, the predominant process that is validated might change. The Utah arrays are in the aSTG, which, in our results, is mostly influenced by top-down processes. This region is distinct from earlier auditory processing areas, such as the posterior STG, which may prioritize bottom-up processing.

We further hypothesize that when top-down cues are strong, this integration in the aSTG is more pronounced, reflecting the influence of lexical and semantic contexts on phonetic processing. Conversely, when top-down information is weaker or less predictive, earlier auditory regions like the posterior STG may dominate, emphasizing bottom-up processing. This might explain why we find stronger semantic activation during the natural speech task in comparison to the semantic decision task. Further experiments would be necessary to systematically test this hypothesis.

In summary, our study does not dismiss the role of bottom-up processing, but rather highlights how semantic prediction can reorganize neural dynamics associated with phonetic processing. We have now conveyed these points more clearly in the Discussion.

Comment R3.4 *To wit, the authors interpret the observed phonetic–semantic alignment as evidence for predictive coding. While the results are certainly consistent with top-down influence, I find the invocation of "predictive coding" somewhat underspecified. Predictive coding, on its best*

day, entails some specific computational and formal commitments... but those never seem to get specified. Leaving the reader wondering: 1) What exactly is being predicted (feature identity, category membership, probability distributions over lexical candidates, semantic features)? 2) In what representational format are these predictions encoded? 3)What constitutes the error signal, and how would it be expressed in the observed manifolds? 4)Through what circuit-level mechanism would semantic-level information reshape phonetic population geometry?
As currently presented, the term risks functioning as a broad explanatory label rather than a mechanistic account. I encourage the authors either to (i) more precisely articulate what aspects of predictive coding theory are directly supported by their findings, or (ii) frame their conclusions more conservatively as evidence for top-down semantic modulation of phonetic population dynamics, without overcommitting to a specific predictive coding framework. Clarifying this would substantially strengthen the theoretical contribution of the manuscript.

Answer:

We appreciate the reviewer's critique regarding our use of the term "predictive coding." It is correct that this term entails broad specific computational and formal commitments, which we do not satisfy with our specific findings. We have revised our language to more accurately reflect the nature of our results. Instead of invoking "predictive coding," we now frame our findings as evidence for top-down semantic modulation of phonetic population dynamics. We have now throughout revised the manuscript accordingly.

Comment R3.5 *Secondly, the semantic categorization task likely induces strong top-down expectations. This is not a weakness—in fact, it is a useful manipulation—but it raises an important question about generality. I wonder to what extent might the observed reorganization of phonetic manifolds reflect task-driven predictive pressure rather than intrinsic, spontaneously engaged predictive transforms during everyday speech perception? Of course, this is not a real problem for the manuscript as it stands in its conclusoins now, as the inclusion of natural speech perception data is a major asset of the study and approach. However, the contrast between task and natural speech could be more explicitly leveraged to address this concern. For example: 1) Are the manifold geometries qualitatively similar across task and naturalistic contexts? I don't think this was there and I missed this... 2) Is the strength of phonetic–semantic alignment modulated by task demands? 3)Does the top-down alignment persist, attenuate, or reorganize in the absence of explicit semantic categorization goals? Even a more explicit discussion of these issues would help situate the findings within a broader theory of speech processing beyond high-predictability laboratory contexts.*

Answer: We thank the reviewer for questioning the generalizability of the manifold geometries across tasks. We first would like to highlight that in the manuscript, we did compare the trajectories of the phoneme kernels between the semantic categorization task and natural speech using canonical correlation analysis (CCA), similarly to what is performed in the literature, and found a significant overlap between the two (Fig. 3b). CCA could not be used for semantics, as the semantic features are not the same in task versus natural speech (task-defined kernels in the task: animal, object; Lancaster sensorimotor norms for natural speech).

Beyond CCA, it is difficult to directly compare the neural manifold geometries between the task and natural speech. The reason is that the PCA is not performed on the same units, which are

difficult to track along extensive time windows. By contrast, a comparison between phonetic and semantic spaces is possible (see our answer to the next question).

The strength of phonetic-semantic alignment is further addressed in our response to question R1.1., where we showed that the alignment survives when controlling for word duration, word frequency, GPT-2 surprisal, and phonotactic probability.

We now acknowledge this point in the Discussion.

Comment R3.6 *The identification of separable semantic and phonetic subspaces is exciting. However, the manuscript could further explore what this implies about representational organization: 1) Does functional separation in low-dimensional space imply partial orthogonality of coding axes? 2)How stable are these subspaces across time and conditions? 3)Does semantic prediction rotate, compress, or otherwise geometrically transform the phonetic manifold? The geometric interpretation is one of the most interesting aspects of the work. Expanding on the nature of these transformations—rather than focusing primarily on their predictive interpretation—could broaden the manuscript's impact and connect it more deeply to the emerging literature on population coding geometry.*

Answer: These are excellent remarks regarding the interpretation of variations in manifold geometry. They prompted us to delve deeper into the implications of our results concerning representational organization, which in the end strengthen both the theoretical and methodological contributions of our study.

To explore whether functional separation in low-dimensional space implies orthogonality of coding axes, we calculated the principal angles between the eigenvectors corresponding to the phonetic and semantic subspaces, for each participant and both the task and natural speech conditions. This analysis revealed that while there was some degree of overlap, the semantic and phonetic subspaces maintained a significant level of orthogonality, suggesting functional separation in their coding axes. We then further characterized the exact relationship between these spaces, first by comparing the dot product between the first three eigenvectors of each space, and second by determining the nature of the transformation (translation, scaling, rotation, or a combination of those).

The results are shown in figures R5 and R6. Across the four datasets, the most consistent result was that phonetic and semantic spaces were not orthogonal. Each participant and condition showed at least one relatively small principal angle, indicating at least one shared population dimension between phonetic and semantic spaces. Mean subspace overlap was 0.59 in Participant 1 task, 0.95 in Participant 2 task, 0.53 in Participant 1 speech, and 0.60 in Participant 3 speech.

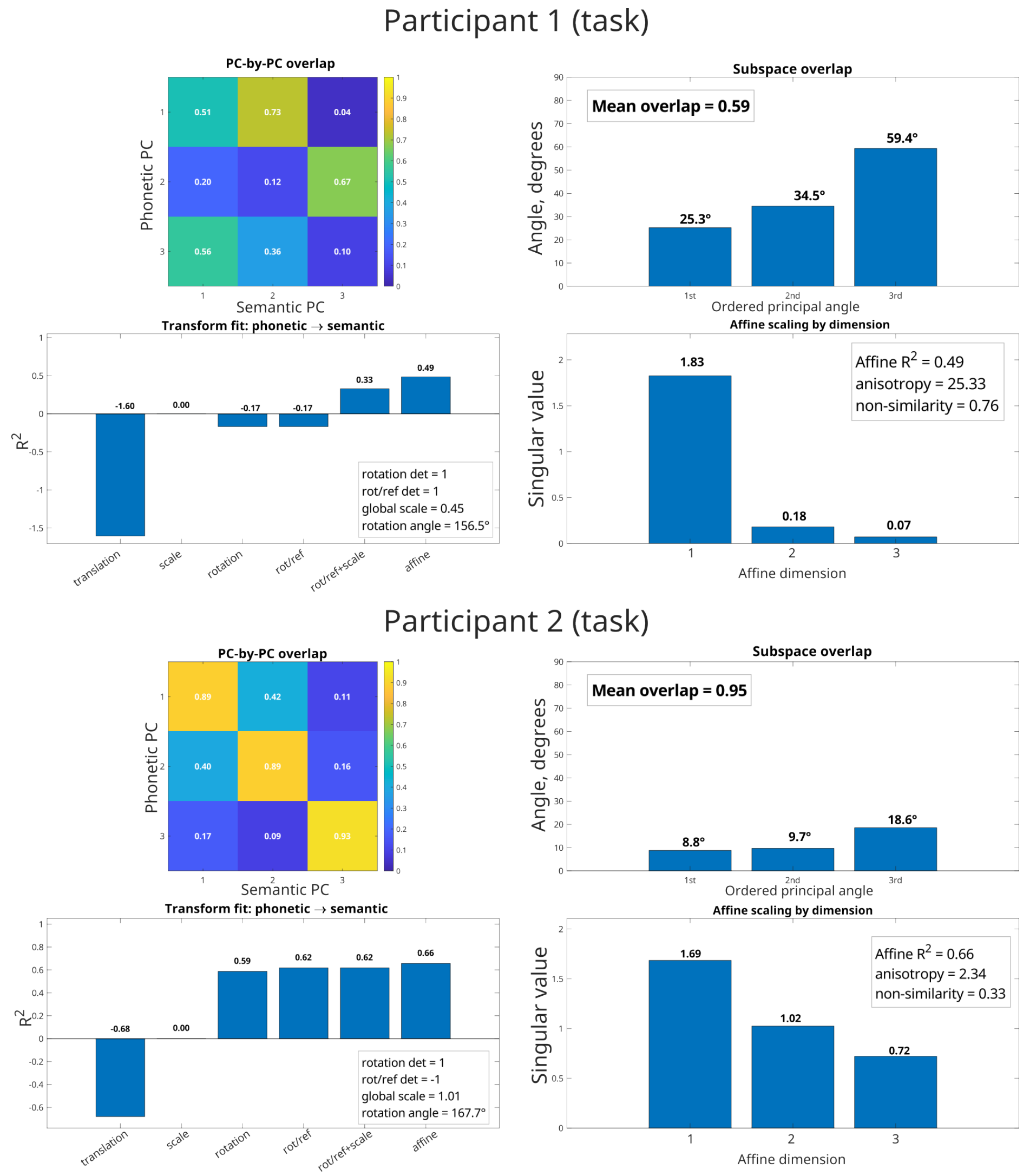

Figure R5: **Geometry of phonetic and semantic manifolds during the semantic categorization task.** For both task participants, the first panel shows the absolute PC-by-PC overlap between the first three phonetic and semantic PC eigenvectors. Brighter values indicate stronger alignment between individual population axes. The second panel shows the principal angles between the three-dimensional phonetic and semantic subspaces. Smaller angles indicate stronger subspace overlap. The third panel shows how well the phonetic point cloud could be transformed to predict the semantic point cloud using increasingly flexible transformations: translation, uniform scaling, rotation, rotation/reflection, rotation/reflection plus global scaling, and full affine mapping. The fourth panel shows the singular values of the full affine transformation, indicating dimension-specific scaling or compression. Participant 1 showed partial phonetic– semantic overlap with mixed PC axes and a better fit for the affine transform than for simpler transformations. Participant 2 showed the strongest phonetic–semantic overlap, with small principal angles and a transformation profile consistent with a largely orthogonal mapping between spaces. Participant 2's result should be interpreted cautiously because they had sparser task responses than Participant 1.

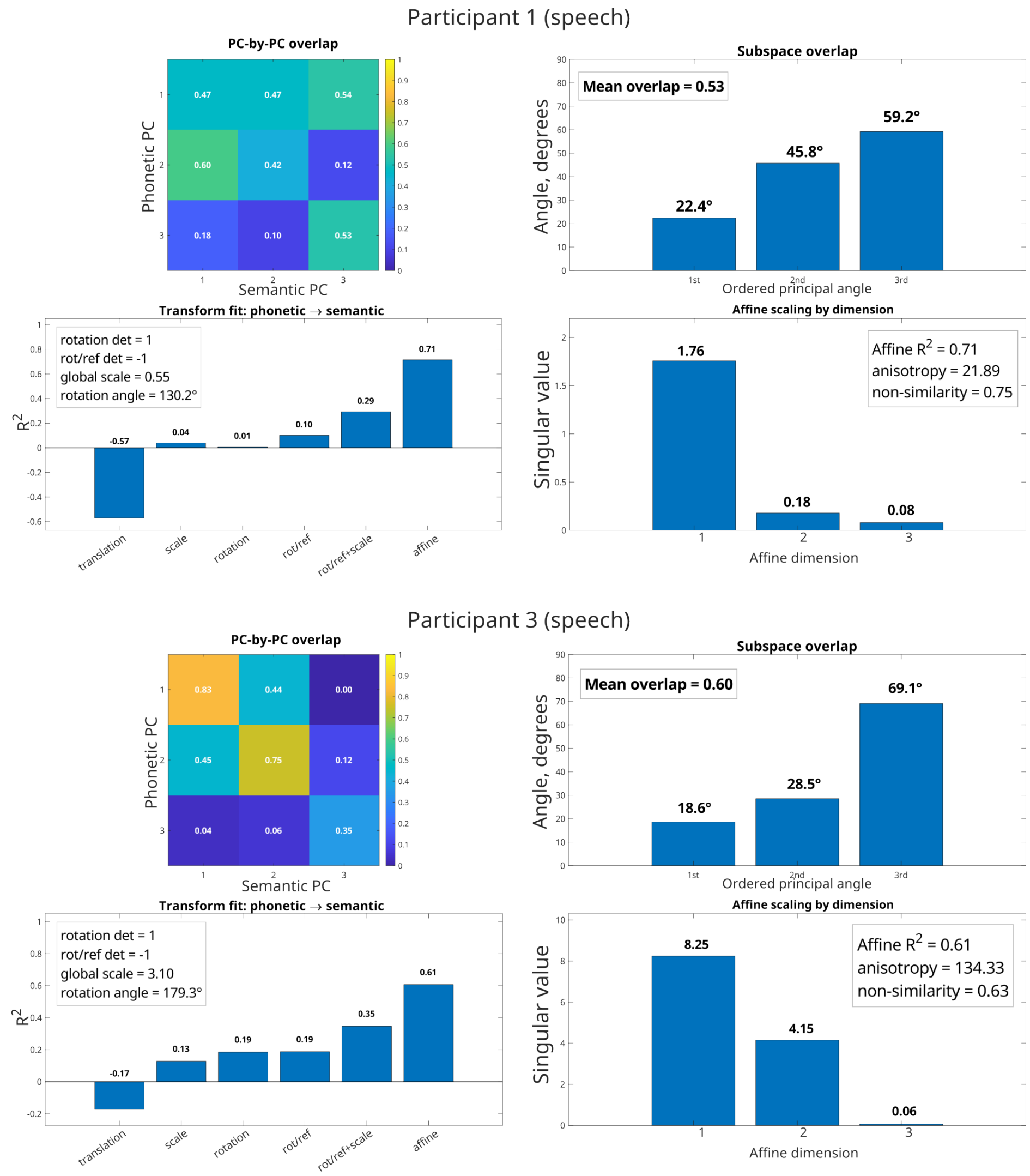

Figure R6: **Geometry of phonetic and semantic spaces during natural speech perception** The same geometry analysis was applied to the speech-listening data. Participant 1 showed partial overlap between phonetic and semantic spaces, with one relatively shared dimension and two more distinct dimensions. Participant 3 also showed partial overlap, with stronger alignment in the first two dimensions and a more separate third dimension. In both speech participants, full affine transformations fit the point-cloud relationship better than simple rotations, indicating that phonetic and semantic spaces shared some population structure but differed in dimension-specific scaling

Participant 1 showed a similar degree of phonetic–semantic overlap in the task and speech conditions. The mean overlap was 0.59 in task and 0.53 in speech, and the angle structure was similar across the two settings: both had one clearly shared population dimension, one intermediate dimension, and one more separated dimension. This suggests that, within Participant 1, phonetic and semantic representations showed a comparable degree of partial overlap across task and speech-listening contexts.

The two ”speech participants” also showed a similar overall pattern. Participant 1 speech and Participant 3 speech both showed partial phonetic–semantic overlap, with mean overlaps of 0.53

and 0.60, respectively. The precise dimensional structure differed: Participant 1 speech showed one strongly shared dimension and two more distinct dimensions, whereas Participant 3 speech showed two shared dimensions and one more clearly separate dimension. Nevertheless, both speech participants showed measurable phonetic–semantic subspace overlap, indicating that this pattern was not unique to one participant.

Participant 2 task was the main outlier, showing much stronger overlap than the other datasets. Because Participant 2 had sparse task responses, this result may partly reflect the dominance of a small number of responsive units in both phonetic and semantic PCA spaces. Therefore, we interpret Participant 2 as showing strong overlap of the dominant low-dimensional axes, while avoiding the stronger claim that this reflects a broadly distributed shared manifold.

Overall, the geometry analysis indicates that phonetic and semantic representations are not implemented in fully separate neural subspaces. Instead, they occupy partially overlapping population spaces. At the same time, except for Participant 2 in the task condition, the relationship between phonetic and semantic point clouds was not well captured by a simple rotation. In most datasets, affine transformations provided better fits and showed unequal singular values, indicating dimension-specific stretching or compression. Thus, the geometry of phonetic and semantic coding is best described as partial overlap with participant- and condition-specific transformations, rather than complete separation or complete identity of the two representational spaces.

We have now added a paragraph to the Results briefly reporting the main findings, as well as a new, more exhaustive section in the Supplementary material:

Main text: Geometry of phonetic and semantic low-dimensional spaces

To further address the nature of the relationship between phonetic and lexico-semantic representations, we quantified the geometric alignment between the phonetic and semantic neural manifolds. For each participant and condition, we quantified the principal angles between the three-dimensional PC phonetic and lexico-semantic spaces. Our analysis revealed that phonetic and semantic manifolds are not orthogonal; instead, they exhibit significant partial overlap, sharing at least one dominant population axis (Supplementary Fig. R5, R6). The principal angles were 25.3°, 34.5°, and 59.4° for Participant 1 in the task condition; 8.8°, 9.7°, and 18.6° for Participant 2 in the task condition; 22.4°, 45.8°, and 59.2° for Participant 1 during natural speech; and 18.6°, 28.5°, and 69.1° for Participant 3 during natural speech.

We also summarized the overall subspace overlap as the mean squared cosine of the three principal angles, where values near 1 indicate strong overlap and values near 0 indicate near-orthogonal subspaces. Mean subspace overlap was 0.59 in Participant 1 task, 0.95 in Participant 2 task, 0.53 in Participant 1 speech, and 0.60 in Participant 3 speech. Thus, across participants and paradigms, phonetic and lexico-semantic representations shared at least one dominant population axis while also retaining separable dimensions. This partial geometrical alignment suggests that phonetic and lexico-semantic dynamics are functionally distinguishable but embedded within partially shared population space. The high degree of overlap in Participant 2 likely reflects sparse coding, i.e. that both phonetic and semantic PCA spaces were dominated by the same small set of responsive units, rather than indicating a broadly distributed shared phonetic–semantic manifold (Supplementary Figs. 1a and 4).

Finally, the relationship between phonetic and semantic manifolds was not well captured by a simple rotation. In most datasets, other affine transformations provided better fits and showed unequal singular values, indicating dimension-specific stretching or compression. Thus, the geometry of phonetic and semantic coding is best described as partial overlap with participant- and condition-specific transformations, rather than complete separation of the two representational spaces (Supplementary material).

Supplementary material: Geometry of phonetic and semantic low-dimensional spaces

To further characterize the relationship between phonetic and semantic representations, we quantified the geometry of the low-dimensional population spaces obtained from the phonetic and semantic mTRF models. The goal of this analysis was to test whether phonetic and semantic responses occupied independent neural subspaces, shared the same low-dimensional space, or showed partial overlap.

For each participant and condition, we extracted the first three principal components from the phonetic and semantic response spaces. Each principal component corresponds to a PCA eigenvector, or population axis, defined by one weight per unit. Thus, the first three phonetic eigenvectors defined a three-dimensional phonetic subspace in unit space, and the first three semantic eigenvectors defined a three-dimensional semantic subspace in the same unit space.

We first quantified alignment between individual phonetic and semantic eigenvectors. Specifically, we computed the absolute dot product between each of the first three phonetic PC eigenvectors and each of the first three semantic PC eigenvectors. This produced a 3 x 3 PC-by-PC overlap matrix. Values close to 1 indicate that the corresponding phonetic and semantic population axes are strongly aligned, whereas values close to 0 indicate near-orthogonality. Because the sign of each PCA axis is arbitrary, we used the absolute value of the dot product.

We then quantified the overlap between the full three-dimensional phonetic and semantic subspaces using principal angles. To compute these angles, we formed a 3 x 3 matrix containing all pairwise dot products between the first three phonetic eigenvectors and the first three semantic eigenvectors. We then applied singular value decomposition to this matrix. The singular values are the cosines of the principal angles between the two subspaces, so the angles were obtained by taking the inverse cosine of the singular values. The first principal angle corresponds to the best-aligned population axis shared between the two subspaces, the second angle corresponds to the next-best independent alignment, and the third angle corresponds to the final independent alignment. Small principal angles indicate strong subspace overlap, whereas angles near 90 degrees indicate near-orthogonality. We summarized the overall subspace overlap as the mean squared cosine of the three principal angles, where values near 1 indicate strong overlap and values near 0 indicate near-orthogonal subspaces.

Finally, we performed a descriptive analysis of point-cloud geometry. This analysis used the PCA score matrices, i.e. the coordinates of observations in the first three PCs. The phonetic and semantic spaces were each represented as observation-by-PC matrices. We centered both point clouds by subtracting their column means and then tested how well increasingly flexible transformations could map the phonetic coordinates onto the semantic coordinates. For translation only,

we shifted the phonetic point cloud to the semantic mean. For uniform scaling, we estimated the single scalar multiplier that minimized the squared error between the scaled phonetic coordinates and the semantic coordinates. For rotation only, we estimated the orthogonal matrix that minimized the squared error between the phonetic and semantic point clouds, using singular value decomposition of the cross-covariance matrix, while constraining the determinant to be +1. For rotation/reflection, we used the same orthogonal Procrustes solution but allowed the determinant to be either +1 or -1. For rotation/reflection plus global scaling, we first estimated the best orthogonal transform and then estimated a single global scale factor. For the full affine model, we solved a linear regression mapping the phonetic coordinates to the semantic coordinates, allowing translation, rotation, reflection, non-uniform scaling, compression, expansion, and shear-like transformations.

For each transformation, we computed an R-squared value comparing the predicted semantic coordinates to the observed semantic coordinates. Negative R-squared values indicate that the transformation predicted the semantic coordinates worse than simply predicting their mean. For the full affine transform, we also computed the singular values of the affine matrix. These singular values quantify scaling along the main axes of the affine transformation: similar singular values indicate approximately uniform scaling, whereas strongly unequal singular values indicate dimension-specific stretching or compression. We also computed the anisotropy ratio, defined as the largest singular value divided by the smallest singular value, and a non-similarity index quantifying how far the affine transform was from a simple rotation/reflection plus uniform scaling.

Across the four datasets, the most consistent result was that phonetic and semantic spaces were not orthogonal. Each participant and condition showed at least one relatively small principal angle, indicating at least one shared population dimension between phonetic and semantic spaces. Mean subspace overlap was 0.59 in Participant 1 task, 0.95 in Participant 2 task, 0.53 in Participant 1 speech, and 0.60 in Participant 3 speech.

Participant 1 showed a similar degree of phonetic–semantic overlap in the task and speech conditions. The mean overlap was 0.59 in task and 0.53 in speech, and the angle structure was similar across the two settings: both had one clearly shared population dimension, one intermediate dimension, and one more separated dimension. This suggests that, within Participant 1, phonetic and semantic representations showed a comparable degree of partial overlap across task and speech-listening contexts.

The two speech participants also showed a similar overall pattern. Participant 1 speech and Participant 3 speech both showed partial phonetic–semantic overlap, with mean overlaps of 0.53 and 0.60, respectively. The precise dimensional structure differed: Speech Participant 1 showed one strongly shared dimension and two more distinct dimensions, whereas speech Participant 3 showed two shared dimensions and one more clearly separate dimension. Nevertheless, both speech participants showed measurable phonetic–semantic subspace overlap, indicating that this pattern was not unique to one participant.

Participant 2 task was the main outlier, showing much stronger overlap than the other datasets. Because Participant 2 had sparse task responses, this result may partly reflect the dominance of a small number of responsive units in both phonetic and semantic PCA spaces. Therefore, we interpret Participant 2 as showing strong overlap of the dominant low-dimensional axes, while avoiding the stronger claim that this reflects a broadly distributed shared manifold.

Overall, the geometry analyses indicate that phonetic and semantic representations are not implemented in fully separate neural subspaces. Instead, they occupy partially overlapping population spaces. At the same time, except for Participant 2 in the task condition, the relationship between phonetic and semantic point clouds was not well captured by a simple rotation. In most datasets, affine transformations provided better fits and showed unequal singular values, indicating dimension-specific stretching or compression. Thus, the geometry of phonetic and semantic coding is best described as partial overlap with participant- and condition-specific transformations, rather than complete separation or complete identity of the two representational spaces.

Comment R3.7 *The failure to detect the effect in adjacent ECoG recordings is compelling and important, and worrisome for people who can't get spike data! It reinforces the idea that fine-grained population structure at the spiking level contains information that may not survive spatial averaging. A slightly more explicit discussion of what this implies about measurement scale and representational granularity would further strengthen the translational relevance of the findings. I would love to hear more of what the authors think this means and its implications for future research.*

Answer: We appreciate the reviewer's help to strengthen the translational relevance of our results.

Our results suggest that for higher-level cognitive processes, such as transitions between levels of language processing (e.g., from phonetic to semantic), sparse and precise neuronal activity patterns are crucial. These processes may not leave robust traces in coarser measures like subdural ECoG field potential, which integrate signals over larger populations and areas. This highlights the unique value of ensemble recordings in studying complex cognitive functions, where the granularity of neural representations is key. Conversely, for lower-level auditory processes, broader neuronal activations might be sufficient to drive detectable signals in ECoG.

We also acknowledge that while spiking activity is central to our findings, other factors such as oscillatory dynamics could complement our understanding of neural representations. These dynamics are reflected at the (intracortical) LFP level, and are likely to be involved in important aspects of speech processing, for instance by gating neuronal predictions according to distant (e.g. frontal) representations [8]. The detailed exploration of the interactions between LFP and neuronal spiking activity will be described more extensively in further work from our labs.

For future research, we believe that multiscale and distributed nature of the mechanisms involved in the neural processing of speech will be better elucidated with multiscale (single-unit and LFP) level and spatially extended recordings in humans. We have extended the Discussion section to include these points.

Comment R3.8 *Overall this is a beautifully executed study leveraging rare human intracortical data to address a foundational question about hierarchical speech and language processing. The population-level manifold approach is a major strength and provides a valuable demonstration of how neural geometry can reveal structure in encoding schemes that might otherwise remain hidden. By more clearly positioning the work relative to the extensive literature on top-down lexical and semantic influences—and by sharpening the theoretical claims regarding predictive coding—the*

manuscript will make a significant and conceptually precise contribution to the study of speech perception and neural population dynamics.

Answer: Thank you, and we hope the revised version appropriately addressed your comments.

References

[1] Lionel Barnett and Anil K. Seth. "Detectability of Granger Causality for Subsampled Continuous-Time Neurophysiological Processes". In: *Journal of Neuroscience Methods* 275 (Jan. 2017), pp. 93–121. ISSN: 01650270. DOI: 10.1016/j.jneumeth.2016.10.016. (Visited on 03/31/2026).

[2] Thomas G. Bever and David Poeppel. "Analysis by Synthesis: A (Re-)Emerging Program of Research for Language and Vision". In: *Biolinguistics* 4.2-3 (Sept. 2010), pp. 174–200. ISSN: 1450-3417. DOI: 10.5964/bioling.8783. (Visited on 10/20/2023).

[3] Gavin M. Bidelman, Claire Pearson, and Ashleigh Harrison. "Lexical Influences on Categorical Speech Perception Are Driven by a Temporoparietal Circuit". In: *Journal of Cognitive Neuroscience* 33.5 (Apr. 2021), pp. 840–852. ISSN: 0898-929X, 1530-8898. DOI: 10.1162/jocn_a_01678. (Visited on 03/31/2026).

[4] Michael P. Broderick, Andrew J. Anderson, and Edmund C. Lalor. "Semantic Context Enhances the Early Auditory Encoding of Natural Speech". In: *Journal of Neuroscience* 39.38 (Sept. 2019), pp. 7564–7575. ISSN: 0270-6474, 1529-2401. DOI: 10.1523/JNEUROSCI.0584-19.2019. (Visited on 04/09/2026).

[5] Julio I. Chapeton et al. "Micro-Scale Functional Modules in the Human Temporal Lobe". In: *Nature Communications* 13.1 (Oct. 2022), p. 6263. ISSN: 2041-1723. DOI: 10.1038/s41467-022-34018-w. (Visited on 04/07/2026).

[6] Donna Coch and Priya Mitra. "Word and Pseudoword Superiority Effects Reflected in the ERP Waveform". In: *Brain research* 1329 (May 2010), pp. 159–174. ISSN: 0006-8993. DOI: 10.1016/j.brainres.2010.02.084. (Visited on 03/31/2026).

[7] W. F. Ganong. "Phonetic Categorization in Auditory Word Perception". In: *Journal of Experimental Psychology. Human Perception and Performance* 6.1 (Feb. 1980), pp. 110–125. ISSN: 0096-1523. DOI: 10.1037//0096-1523.6.1.110.

[8] Anne-Lise Giraud and Luc H. Arnal. "Hierarchical Predictive Information Is Channeled by Asymmetric Oscillatory Activity". In: *Neuron* 100.5 (Dec. 2018), pp. 1022–1024. ISSN: 08966273. DOI: 10.1016/j.neuron.2018.11.020. (Visited on 01/22/2020).

[9] Nick V. Hahn et al. "Long-Term Performance of Intracortical Microelectrode Arrays in 14 BrainGate Clinical Trial Participants". In: *medRxiv: The Preprint Server for Health Sciences* (July 2025), p. 2025.07.02.25330310. DOI: 10.1101/2025.07.02.25330310.

[10] Morris Halle and Kenneth Stevens. "Analysis by Synthesis". In: *Proceeding of the Seminar on Speech Compression and Processing*. Paper D7 II (1959), p. 1. DOI: sdf.

[11] Stefan Haufe et al. "On the Interpretation of Weight Vectors of Linear Models in Multivariate Neuroimaging". In: *NeuroImage* 87 (Feb. 2014), pp. 96–110. ISSN: 10538119. DOI: 10.1016/j.neuroimage.2013.10.067. (Visited on 08/05/2021).

[12] Gregory Hickok. *Wired for Words: The Neural Architecture of Language*. Cambridge, MA, USA: MIT Press, Nov. 2025. ISBN: 978-0-262-55341-4.

[13] Marjolijn Hoekert et al. "Time Course of the Involvement of the Right Anterior Superior Temporal Gyrus and the Right Fronto-Parietal Operculum in Emotional Prosody Perception". In: *PLoS ONE* 3.5 (May 2008), e2244. ISSN: 1932-6203. DOI: 10.1371/journal.pone.0002244. (Visited on 04/07/2026).

[14] International Brain Laboratory et al. "A Brain-Wide Map of Neural Activity during Complex Behaviour". In: *Nature* 645.8079 (Sept. 2025), pp. 177–191. ISSN: 1476-4687. DOI: 10.1038/s41586-025-09235-0. (Visited on 04/02/2026).

[15] Anthony I. Jang et al. "Human Cortical Neurons in the Anterior Temporal Lobe Reinstate Spiking Activity during Verbal Memory Retrieval". In: *Current Biology* 27.11 (June 2017), 1700–1705.e5. ISSN: 09609822. DOI: 10.1016/j.cub.2017.05.014. (Visited on 03/31/2026).

[16] Karim Jerbi et al. "Saccade Related Gamma-Band Activity in Intracerebral EEG: Dissociating Neural from Ocular Muscle Activity". In: *Brain Topography* 22.1 (June 2009), pp. 18–23. ISSN: 1573-6792. DOI: 10.1007/s10548-009-0078-5. (Visited on 04/08/2026).

[17] Arjun R. Khanna et al. "Single-Neuronal Elements of Speech Production in Humans". In: *Nature* (Jan. 2024). ISSN: 0028-0836, 1476-4687. DOI: 10.1038/s41586-023-06982-w. (Visited on 02/08/2024).

[18] Stefan Koelsch. "Brain Correlates of Music-Evoked Emotions". In: *Nature Reviews Neuroscience* 15.3 (Mar. 2014), pp. 170–180. ISSN: 1471-003X, 1471-0048. DOI: 10.1038/nrn3666. (Visited on 04/07/2026).

[19] Christopher K. Kovach et al. "Manifestation of Ocular-Muscle EMG Contamination in Human Intracranial Recordings". In: *NeuroImage* 54.1 (Jan. 2011), pp. 213–233. ISSN: 1053-8119. DOI: 10.1016/j.neuroimage.2010.08.002. (Visited on 04/08/2026).

[20] Erin M. Kunz et al. "Inner Speech in Motor Cortex and Implications for Speech Neuroprostheses". In: *Cell* 188.17 (Aug. 2025), 4658–4673.e17. ISSN: 0092-8674, 1097-4172. DOI: 10.1016/j.cell.2025.06.015. (Visited on 04/07/2026).

[21] Marta Kutas and Kara D. Federmeier. "Thirty Years and Counting: Finding Meaning in the N400 Component of the Event-Related Brain Potential (ERP)". In: *Annual Review of Psychology* 62.1 (Jan. 2011), pp. 621–647. ISSN: 0066-4308, 1545-2085. DOI: 10.1146/annurev.psych.093008.131123. (Visited on 07/31/2023).

[22] Katarína Labancová and Nina Kazanina. "Dynamical Motifs for Computations in Language". In: *Cognitive Neuroscience* 16.1-4 (Oct. 2025), pp. 83–84. ISSN: 1758-8928. DOI: 10.1080/17588928.2025.2581573. (Visited on 04/07/2026).

[23] Arianna N. LaCroix, Alvaro F. Diaz, and Corianne Rogalsky. "The Relationship between the Neural Computations for Speech and Music Perception Is Context-Dependent: An Activation Likelihood Estimate Study". In: *Frontiers in Psychology* 6 (Aug. 2015), p. 1138. ISSN: 1664-1078. DOI: 10.3389/fpsyg.2015.01138. (Visited on 04/07/2026).

[24] Yair Lakretz et al. "Single-Cell Activity in Human STG during Perception of Phonemes Is Organized According to Manner of Articulation". In: *NeuroImage* 226 (Feb. 2021), p. 117499. ISSN: 1053-8119. DOI: 10.1016/j.neuroimage.2020.117499. (Visited on 10/30/2023).

[25] Mikhail A. Lebedev et al. "Analysis of Neuronal Ensemble Activity Reveals the Pitfalls and Shortcomings of Rotation Dynamics". In: *Scientific Reports* 9.1 (Dec. 2019). ISSN: 2045-2322. DOI: 10.1038/s41598-019-54760-4. (Visited on 12/04/2020).

[26] Matthew K. Leonard et al. "Large-Scale Single-Neuron Speech Sound Encoding across the Depth of Human Cortex". In: *Nature* (Dec. 2023), pp. 1–10. ISSN: 1476-4687. DOI: 10.1038/s41586-023-06839-2. (Visited on 12/18/2023).

[27] Christophe Leys et al. "Detecting Outliers: Do Not Use Standard Deviation around the Mean, Use Absolute Deviation around the Median". In: *Journal of Experimental Social Psychology* 49.4 (July 2013), pp. 764–766. ISSN: 0022-1031. DOI: 10.1016/j.jesp.2013.03.013. (Visited on 05/31/2026).

[28] Yi Liu et al. "Early Top-Down Modulation in Visual Word Form Processing: Evidence From an Intracranial SEEG Study". In: *Journal of Neuroscience* 41.28 (July 2021), pp. 6102–6115. ISSN: 0270-6474, 1529-2401. DOI: 10.1523/JNEUROSCI.2288-20.2021. (Visited on 03/31/2026).

[29] Sahil Luthra. "The Role of the Right Hemisphere in Processing Phonetic Variability Between Talkers". In: *Neurobiology of Language* 2.1 (Feb. 2021), pp. 138–151. ISSN: 2641-4368. DOI: 10.1162/nol_a_00028. (Visited on 04/09/2026).

[30] Dermot Lynott et al. *The Lancaster Sensorimotor Norms: Multidimensional Measures of Perceptual and Action Strength for 40,000 English Words*. May 2019. DOI: 10.31234/osf.io/ktjwp. (Visited on 09/22/2023).

[31] Donald M. Mackay. "Towards an Information-Flow Model of Human Behavior". In: *British Journal of Psychology* 47 (1956), pp. 30–43. ISSN: 2044-8295. DOI: 10.1111/j.2044-8295.1956.tb00559.x.

[32] Monika S. Mellem et al. "Sentence Processing in Anterior Superior Temporal Cortex Shows a Social-Emotional Bias". In: *Neuropsychologia* 89 (Aug. 2016), pp. 217–224. ISSN: 00283932. DOI: 10.1016/j.neuropsychologia.2016.06.019. (Visited on 07/31/2023).

[33] Joanne L. Miller and Alvin M. Liberman. "Some Effects of Later-Occurring Information on the Perception of Stop Consonant and Semivowel". In: *Perception & Psychophysics* 25.6 (Nov. 1979), pp. 457–465. ISSN: 1532-5962. DOI: 10.3758/BF03213823. (Visited on 03/31/2026).

[34] Charlotte Murphy et al. "Fractionating the Anterior Temporal Lobe: MVPA Reveals Differential Responses to Input and Conceptual Modality". In: *Neuroimage* 147 (Feb. 2017), pp. 19–31. ISSN: 1053-8119. DOI: 10.1016/j.neuroimage.2016.11.067. (Visited on 04/07/2026).

[35] Elliot Murphy. "ROSE: A Universal Neural Grammar". In: *Cognitive Neuroscience* 16.1-4 (Oct. 2025), pp. 49–80. ISSN: 1758-8928, 1758-8936. DOI: 10.1080/17588928.2025.2523875. (Visited on 04/07/2026).

[36] Elliot Murphy et al. "The Spatiotemporal Dynamics of Semantic Integration in the Human Brain". In: *Nature Communications* 14.1 (Oct. 2023), p. 6336. ISSN: 2041-1723. DOI: 10.1038/s41467-023-42087-8. (Visited on 12/17/2023).

[37] E. B. Myers and S. E. Blumstein. "The Neural Bases of the Lexical Effect: An fMRI Investigation". In: *Cerebral Cortex* 18.2 (Feb. 2008), pp. 278–288. ISSN: 1047-3211, 1460-2199. DOI: 10.1093/cercor/bhm053. (Visited on 03/31/2026).

[38] Dionysios Perdikis, Raoul Huys, and Viktor K. Jirsa. “Time Scale Hierarchies in the Functional Organization of Complex Behaviors”. In: *PLoS Computational Biology* 7.9 (Sept. 2011). Ed. by Danielle S. Bassett, e1002198. ISSN: 1553-7358. DOI: 10.1371/journal.pcbi.1002198. (Visited on 07/30/2021).

[39] Camille R. C. Pescatore et al. *Decoding Semantics from Natural Speech Using Human Intracranial EEG*. Feb. 2025. DOI: 10.1101/2025.02.10.637051. (Visited on 04/02/2026).

[40] Ajay S. Pillai and Viktor K. Jirsa. “Symmetry Breaking in Space-Time Hierarchies Shapes Brain Dynamics and Behavior”. In: *Neuron* 94.5 (June 2017), pp. 1010–1026. ISSN: 08966273. DOI: 10.1016/j.neuron.2017.05.013. (Visited on 07/29/2021).

[41] Timothée Proix, Matthew G. Perich, and Tomislav Milekovic. *Misinterpreting the Horseshoe Effect in Neuroscience*. Apr. 2023. DOI: 10.1101/2022.03.04.482986. (Visited on 04/08/2026).

[42] Matthew A. Lambon Ralph et al. “The Neural and Computational Bases of Semantic Cognition”. In: *Nature Reviews Neuroscience* 18.1 (Jan. 2017), pp. 42–55. ISSN: 1471-003X, 1471-0048. DOI: 10.1038/nrn.2016.150. (Visited on 07/31/2023).

[43] Gerald M. Reicher. “Perceptual Recognition as a Function of Meaningfulness of Stimulus Material”. In: *Journal of Experimental Psychology* 81.2 (1969), pp. 275–280. ISSN: 0022-1015. DOI: 10.1037/h0027768.

[44] Peter J. Rousseeuw and Christophe Croux. “Alternatives to the Median Absolute Deviation”. In: *Journal of the American Statistical Association* 88.424 (1993), pp. 1273–1283. ISSN: 0162-1459. DOI: 10.2307/2291267. JSTOR: 2291267. (Visited on 05/31/2026).

[45] Maxwell Shinn. “Phantom Oscillations in Principal Component Analysis”. In: *Proceedings of the National Academy of Sciences* 120.48 (Nov. 2023), e2311420120. ISSN: 0027-8424, 1091-6490. DOI: 10.1073/pnas.2311420120. (Visited on 04/08/2026).

[46] Ali Shojaie and Emily B. Fox. “Granger Causality: A Review and Recent Advances”. In: *Annual Review of Statistics and Its Application* 9.1 (Mar. 2022), pp. 289–319. ISSN: 2326-8298, 2326-831X. DOI: 10.1146/annurev-statistics-040120-010930. (Visited on 09/19/2025).

[47] Shan H. Siddiqi et al. “Causal Mapping of Human Brain Function”. In: *Nature Reviews Neuroscience* 23.6 (June 2022), pp. 361–375. ISSN: 1471-003X, 1471-0048. DOI: 10.1038/s41583-022-00583-8. (Visited on 11/24/2022).

[48] Ediz Sohoglu et al. “Predictive Top-Down Integration of Prior Knowledge during Speech Perception”. In: *Journal of Neuroscience* 32.25 (June 2012), pp. 8443–8453. ISSN: 0270-6474, 1529-2401. DOI: 10.1523/JNEUROSCI.5069-11.2012. (Visited on 04/09/2026).

[49] Yaqing Su et al. “A Deep Hierarchy of Predictions Enables Online Meaning Extraction in a Computational Model of Human Speech Comprehension”. In: *PLOS Biology* 21.3 (Mar. 2023), e3002046. ISSN: 1545-7885. DOI: 10.1371/journal.pbio.3002046. (Visited on 09/29/2025).

[50] Melissa Thye et al. “Intracranial EEG Evidence of Functional Specialization for Taxonomic and Thematic Relations”. In: *Cortex* 140 (July 2021), pp. 40–50. ISSN: 00109452. DOI: 10.1016/j.cortex.2021.03.018. (Visited on 04/02/2026).

[51] Alex P. Vaz et al. “Replay of Cortical Spiking Sequences during Human Memory Retrieval”. In: *Science* 367.6482 (Mar. 2020), pp. 1131–1134. DOI: 10.1126/science.aba0672. (Visited on 04/07/2026).

[52] Weizhen Xie et al. "Neuronal Sequences in Population Bursts Encode Information in Human Cortex". In: *Nature* 635.8040 (Nov. 2024), pp. 935–942. ISSN: 1476-4687. DOI: `10.1038/s41586-024-08075-8`. (Visited on 10/14/2025).

[53] Roland Zahn et al. "Social Concepts Are Represented in the Superior Anterior Temporal Cortex". In: *Proceedings of the National Academy of Sciences* 104.15 (Apr. 2007), pp. 6430–6435. DOI: `10.1073/pnas.0607061104`. (Visited on 04/07/2026).
